# Mitigating protocatechuic acid toxicity in *Vibrio natriegens* enables poly-3-hydroxybutyrate production from an aromatic carbon source

**DOI:** 10.1007/s00253-026-13831-z

**Published:** 2026-04-27

**Authors:** Anna Faber, Roland Politan, Angus Nicol, Simona Della Valle, Benjamin Jenkins, Gavin Flematti, Georg Fritz

**Affiliations:** 1 School of Molecular Sciences, The University of Western Australia, Perth, Australia; 2 Oceans Institute, The University of Western Australia, Perth, Australia; 3 Forrest Research Foundation, Perth, Australia

**Keywords:** Aromatic acids, *Vibrio natriegens*, Bioplastics, Oxidative stress

## Abstract

3,4-Dihydroxybenzoic acid (protocatechuic acid, PCA) is a central intermediate in bacterial aromatic catabolism - a key node for processing aromatic waste streams such as lignin and polyethylene terephthalate (PET) and a compound of growing biotechnological interest for valorising complex aromatic feedstocks. Here, we present a proof-of-concept for upcycling of PCA as a sole carbon source into a non-aromatic, higher-value bioproduct. We show that *Vibrio natriegens*, an emerging microbial chassis, can use PCA to produce poly-3-hydroxybutyrate (PHB), with no additional carbon source such as yeast extract or peptone. Efficient growth on PCA required careful optimisation of substrate concentration, cultivation conditions, and medium composition, e.g. replacing Fe^2^⁺ with Fe^3^⁺. These strategies reduced PCA autoxidation and limited reactive oxygen species (ROS) toxicity. Adaptive laboratory evolution over ~ 1000 generations identified convergent mutations that reveal insights into the architecture of the oxidative stress response in *V. natriegens*, including catalytic enzymes such as a Dyp-type peroxidase, and multiple regulatory proteins including OxyR, MarR, RpoS, ToxS, and RseB. Further mutations implicate cell wall repair and protein quality control in the PCA stress response. Functionally, the adapted strains produced 2.5-fold more PHB in 80% of the time required by the parental strain. Overall, we demonstrate the potential and current limitations of *V.* *natriegens* for the sustainable upcycling of aromatic, waste-derived carbon into value-added bioproducts.

**Graphical abstract:**

**Figure:**
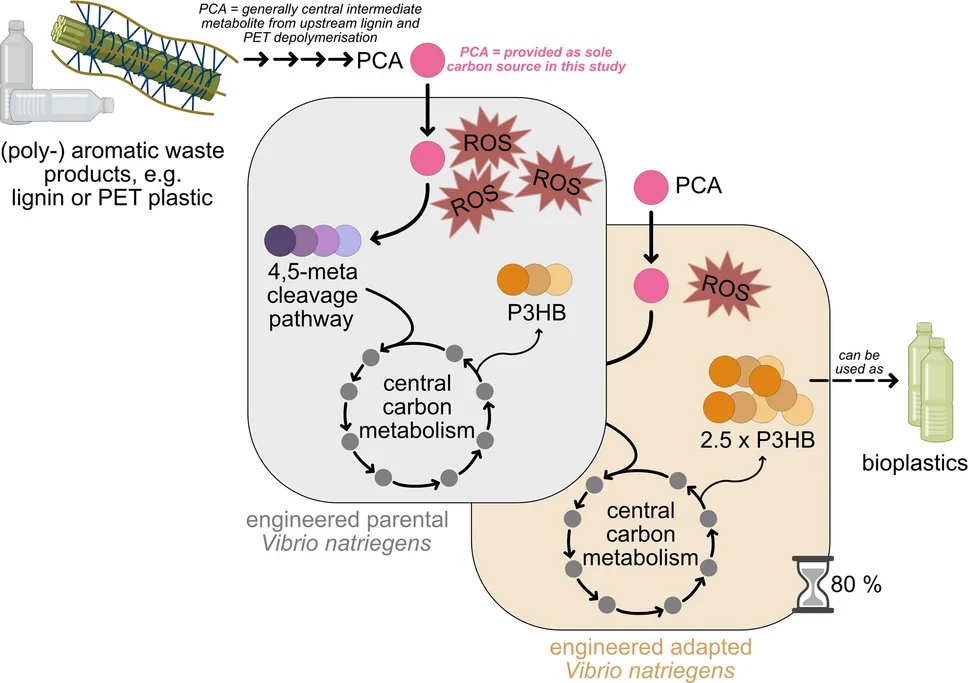

**Supplementary Information:**

The online version contains supplementary material available at 10.1007/s00253-026-13831-z.

## Introduction

Aromatic acids represent a major and largely underutilised carbon reservoir for microbial bioprocessing. They are generated at large scale through the depolymerisation of lignin-rich plant biomass in terrestrial ecosystems and industrial processing as well as through the degradation of synthetic aromatic polymers (Danso et al. 2019; Grevesse et al. 2022; Wu et al. 2016; Zangrando et al. 2019). These compounds enter natural and engineered environments via riverine transport and coastal runoffs, wastewater streams, and plastic waste (Grevesse et al. 2022; Juarez et al. 2005; Opsahl And Benner 1997; Wu et al. 2016). In coastal and marine systems, the aromatic pool is supplemented by compounds released from phytoplankton, seagrasses, and macroalgae (Zangrando et al. 2019). As a result, microorganisms in these environments are chronically exposed to diverse aromatic acids and have evolved specialised pathways for their uptake and catabolism (Grevesse et al. 2022; Juarez et al. 2005; McDonald et al. 2019). These native metabolic capabilities have motivated extensive metabolic engineering efforts to exploit aromatic substrates as alternative carbon sources for lignin valorisation and plastic upcycling.

**Fig. 1:**
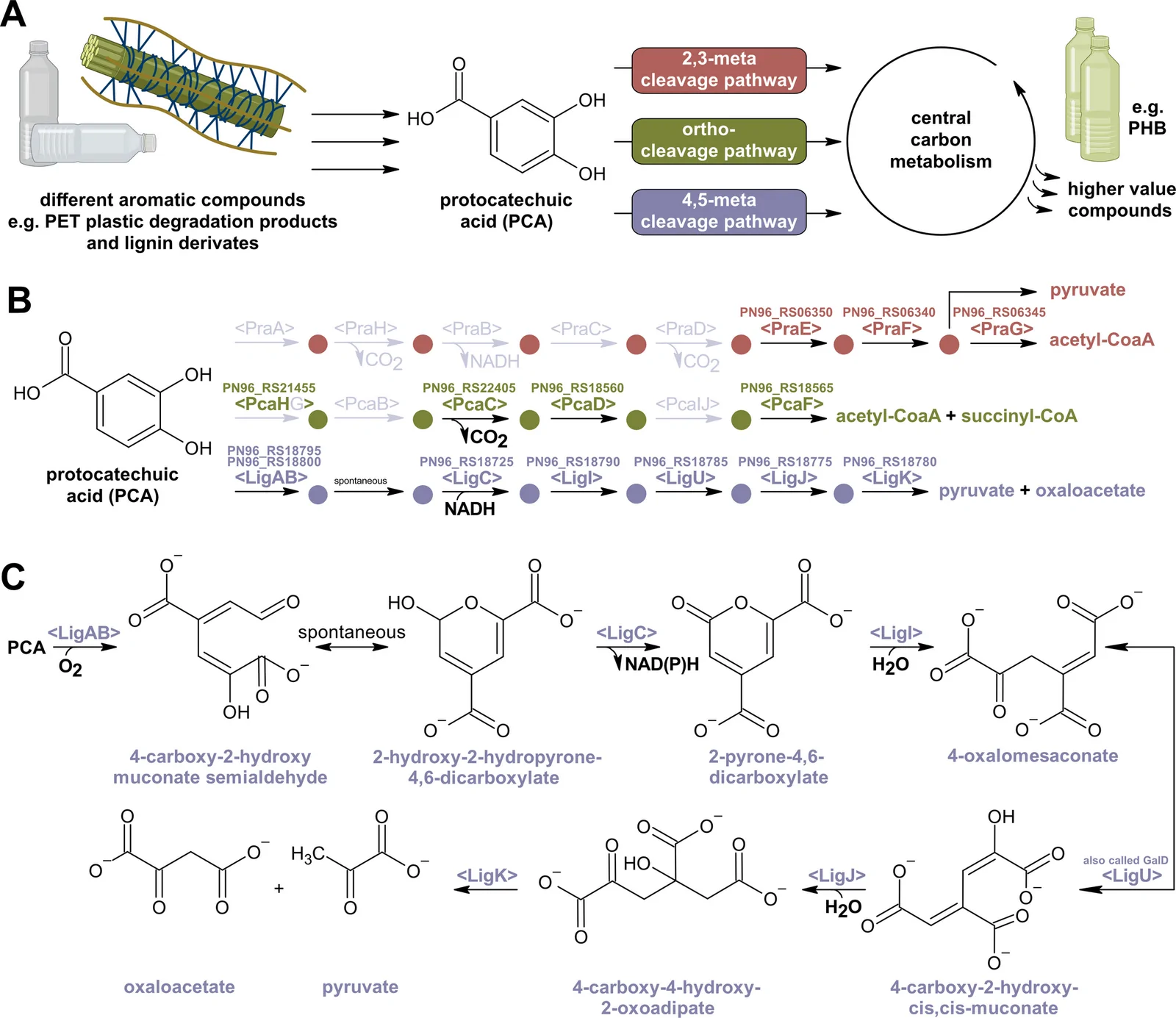
PCA catabolic pathways in *V. natriegens* and other microorganisms. **A** Three distinct microbial pathways channel PCA into central carbon metabolism. **B** PCA degradation pathways across organisms (inspired by Wilkes et al. 2023): the 2,3-meta cleavage pathway in *Bacillus* sp. and *Paenibacillus* sp., the ortho-pathway in *Pseudomonas putida*, *Pseudomonas umsongensis*, and *Rhodococcus jostii*, and the 4,5-meta cleavage pathway in *Sphingomonas paucimobilis*, *Comamonas testosteroni*, and *Vibrio natriegens*. Faint enzymes are absent in *V. natriegens*; bold, coloured enzymes are genome-annotated. **C** Annotated 4,5-meta cleavage pathway for PCA catabolism in *V. natriegens*. Enzyme presence and reaction directionality are based on KEGG annotations

Protocatechuic acid (PCA; 3,4-dihydroxybenzoic acid) is a central intermediate in both natural and engineered aromatic catabolism (Fig. 1A). Diverse aromatic substrates derived from lignin (Kim et al. 2025), synthetic polymers (Feng et al. 2025; Yoshida et al. 2016), and plant phenolics (Tan et al. 2019) are funnelled into PCA prior to aromatic ring cleavage (Fuchs et al. 2011; Wilkes et al. 2023). PCA is metabolised via three alternative pathways: the 2,3-meta cleavage pathway (Crawford 1975; Kasai et al. 2009), the ortho-cleavage (β-ketoadipate) pathway (Harwood et al. 1994; Harwood And Parales 1996; Jiménez et al. 2002; Spence et al. 2020), or the 4,5-meta cleavage pathway (Hädrich et al. 2025; Hara et al. 2003; Ni et al. 2013) (Fig. 1B). These routes directly connect aromatic depolymerisation to the central carbon metabolism while supplying precursors for redox cofactors, siderophores, and specialised metabolites (Pfleger et al. 2008; Pierrel 2017). Accordingly, PCA has been widely exploited as a platform intermediate in engineered systems for lignin (Kim et al. 2025) and PET (Feng et al. 2025; Yoshida et al. 2016) deconstruction and for the biosynthesis of both non-aromatic and aromatic products such as catechol, cis,cis-muconic acid (Johnson et al. 2016; Song et al. 2022; Sonoki et al. 2014; Zhang et al. 2015), vanillin (Sadler And Wallace 2021; Santos et al. 2024; Zhang et al. 2015), alanine (Rosini et al. 2025), lycopene (Diao et al. 2024, 2023), and nylon-polymer precursors (Werner et al. 2021). Substantial metabolic engineering efforts have focused on PCA itself as a production target (Jin et al. 2024; Lubbers And de Vries 2021; Örn et al. 2021), highlighting its value for the chemical and pharmaceutical industries and as a growth-promoting additive in microbial bioprocesses (Tan et al. 2020).

From an engineering perspective, low-molecular weight aromatic acids such as PCA present intrinsic physiological challenges. At cultivation pH, PCA is predominantly dissociated (pKa 4.48), limiting passive diffusion and rendering active uptake via dedicated transport systems necessary (Chae And Zylstra 2006; Fujita et al. 2019; Hosaka et al. 2013; Mori et al. 2018; Nichols and Harwood 1997; Noinaj et al. 2010; Pernstich et al. 2014). In addition, elevated PCA concentrations promote autoxidation and the formation of reactive oxygen species (ROS), which impair growth and impose strong selection pressure on cellular stress-response networks (Ajiboye et al. 2017; Nakai et al. 2001; Tan et al. 2020). Thus, PCA functions both as a metabolic entry point and as a redox challenge that must be managed in PCA-based bioprocesses.

Over the past decades, polyhydroxyalkanoates (PHAs) have attracted attention as biodegradable plastic alternatives. PHA production from aromatic substrates such as ferulate, coumarate, and benzoate has been established in organisms including *Pseudomonas putida*, *Burkholderia* spp., *Halomonas hydrothermalis*, and *Ralstonia eutropha* (Morya et al. 2021; Ramírez-Morales et al. 2021; Salvachúa et al. 2020; Tomizawa et al. 2014; Wang et al. 2025), where PCA functions as a metabolic intermediate (see Supplementary Table 1 for a comprehensive list). However, PCA has been used as a direct carbon source for PHA biosynthesis only with limited success in *Pseudomonas putida* (Tomizawa et al. 2014) or at titres of 0.507 g/L in *Halomonas* sp. Y3 (Tang et al. 2022, p. 202). Overall, the biosynthesis of non-aromatic value-added products such as PHAs from PCA represents a largely unexplored design space in metabolic engineering, particularly given the scale of lignin and PET production (~ 100 Mt and ~ 28 Mt yr⁻^1^, respectively) and the limited recovery of these aromatic resources (Bajwa et al. 2019; Han et al. 2025; Jovanović et al. 2024). Although pure PCA is unlikely to become an industrial feedstock in the near term due to cost and supply limitations (Jin et al. 2024; Lubbers And de Vries 2021; Zheng et al. 2024), establishing PCA-fed bioproduction provides a proof-of-concept framework for future integration into aromatic waste-stream valorisation pipelines.

Here, we address this gap by demonstrating that PCA alone can serve as a carbon source for the biosynthesis of a non-aromatic PHA biopolymer. We engineered *Vibrio natriegens*, the fastest-growing non-pathogenic bacterium reported to date (Hoff et al. 2020) to convert PCA into poly-3-hydroxybutyrate (PHB). *V. natriegens* has emerged as a promising bioproduction chassis due to its ultrafast doubling time, high substrate uptake rates, growth to high cell densities, and halotolerance (Della Valle et al. 2025; Hoff et al. 2020). Originally isolated from a salt marsh environment (Payne et al. 1961), it naturally inhabits coastal systems enriched in terrestrially derived aromatic compounds (Opsahl And Benner 1997) and catabolises several aromatic acids (Hädrich et al. 2025b). In *V.* *natriegens*, PCA is metabolised exclusively via the 4,5-meta cleavage pathway (Hädrich et al. 2025a) (Fig. 1C), directly coupling aromatic degradation to the central metabolism. Using genetic tools for *V. natriegens*, including NT-CRISPR-based genome editing (Stukenberg et al. 2022) and the modular Vnat Collection (Faber et al. 2025), we systematically investigate growth on PCA as a sole carbon source. By integrating pathway engineering with adaptive laboratory evolution (ALE) and targeted media optimisation, we identify key physiological bottlenecks and establish PCA-fed PHB production as a proof of concept for expanding aromatic feedstock utilisation beyond aromatic end products.

## Materials and methods

### Bacterial strains and media

All *Vibrio* *natriegens* strains in this study are listed in Supplementary Table 2. As the reference background, we used a *V.* *natriegens* strain derived from DSM 759 (ATCC 14048) that harbours ΔVNP1 + 2 prophage and Δ*dns* exonuclease deletions for higher transformation efficiency, osmotolerance, and pyruvate production (Pfeifer et al. 2019). Throughout this study, we refer to this strain as the parental strain (PS). Routine cultivation of *V.* *natriegens* was carried out in LBv2 medium on plates or in shake flasks. Liquid cultures were initiated from glycerol stocks of independent biological replicates, prepared by mixing equal volumes of 50% glycerol and stationary phase LBv2 cultures (~ 5 h at 37 °C, 220 rpm) and stored at −80 °C. When required, kanamycin (200 µg/mL) or chloramphenicol (2 µg/mL) was added to LBv2 or MOPS2 minimal medium. *V. natriegens* plates were stored at room temperature to avoid the viable-but-nonculturable (VBNC) state (Weinstock et al. 2016).

*Escherichia coli* DH5α and DH10β were used for propagation of Golden Gate Assembly products, allowing high transformation efficiencies with plasmids up to 5 kb and 5–12 kb, respectively. *E. coli* strains were grown in LBI medium containing appropriate antibiotics (50 µg/mL kanamycin or 25 µg/mL chloramphenicol). Glycerol stocks were generated from overnight cultures (~ 16 h at 37 °C, 220 rpm) using the same procedure as for *V. natriegens*. LBI medium contained tryptone (10 g/L), yeast extract (5 g/L), and NaCl (10 g/L). LBv2 medium was supplemented with an additional V2 salt mixture (NaCl 11.9 g/L, KCl 0.3 g/L, MgCl₂ 2.2 g/L) according to Weinstock et al. 2016. LBI and LBv2 media were sterilised by autoclaving at 121 °C for 15 min. Solid LBI and LBv2 medium was prepared by adding 1.5% (w/v) agar before autoclaving. Antibiotic stock solutions (50 mg/mL kanamycin in water, 25 mg/mL chloramphenicol in 70% ethanol) were added once autoclaved media had cooled below ~50 °C.

For cultivation of *V.* *natriegens* on PCA or glucose as a sole carbon sources, MOPS2 minimal medium was chosen. MOPS2, derived from Neidhardt medium (Neidhardt et al. 1974), was supplemented with 2% (w/v) (342.2 mM) NaCl to mimic ocean salinity. The final 1 × MOPS2 medium (pH 7.2) contained MOPS2 (42 mM), Tricine (4 mM), K_2_SO_4_ (0.276 mM), CaCl_2_ (0.5 μM), MgCl_2_ (0.525 mM), NaCl (total 392.2 mM = 2.29% (w/v)), FeSO_4_ (10 μM), K_2_HPO_4_ (1.32 mM), NH_4_CL (9.5 mM), and the micronutrients, (NH_4_)_6_Mo_7_O_24_ (3 nM), H_3_BO_3_ (0.2 μM), CoCl_2_ (1.5 nM), CuSO_4_ (0.48 nM), MnCl_2_ (4 nM), and ZnSO_4_ (0.48 nM) (for micronutrients stock preparation, see Neidhardt et al. 1974).

MOPS2 minimal medium was modified to improve growth on aromatic acids by replacing Fe^2^⁺ with Fe^3^⁺ and supplementing citrate to enhance iron solubility. Magnesium concentration was increased to support optimal growth of *V.* *natriegens*. To prepare the optimised 10 × MOPS2 stock, 16.744 g MOPS and 1.434 g Tricine were dissolved in 60 mL water, the pH was adjusted to 7.4 with 10 M KOH, and the volume was brought to 88 mL. Subsequently, 0.4 mL of 50 mM FeCl₃ prepared in 100 mM citric acid was added, followed by 10 mL NH₄Cl (1.9 M), 2 mL K₂SO₄ (0.276 M), 0.05 mL CaCl₂ (0.02 M), 11.8 mL MgCl₂ (2.5 M), 20 mL NaCl (5 M), and 0.04 mL micronutrient stock solution.

Carbon sources were dissolved as solids in MOPS2 minimal media before filter sterilisation (pore size 0.22 µm). Carbon sources were either 1 g/L (5.56 mM) glucose (chem-supply GA018-500G) and/or up to 4.5 g/L (29.22 mM) PCA (Sigma-Aldrich 37580-25G-F). We calculate that 99.8% of PCA (pK_a_ 4.48) is dissociated at pH 7.2 (Eq. 1). Culture conditions and media composition for each experiment can be found in Supplementary Table 3.

1 $$pH={pK}_{a}+\mathrm{log}\left(\frac{{A}^{-}}{HA}\right) \underset{\phantom{0}}{\Rightarrow } 7.2=4.48+ \mathrm{log}\left(\frac{{A}^{-}}{HA}\right) {\Rightarrow }\;\frac{{A}^{-}}{HA}=525$$

$$\%\mathrm{dissociated}=\frac{{A}^{-}}{{A}^{-}+\mathit{HA}}\times 100\;{ \% }\underset{\phantom{0}}{\Rightarrow } \frac{525}{525+1}\times 100\;{ \%}=99.8\;{ \%}$$

### Adaptive laboratory evolution of *V. natriegens*

For adaptive laboratory evolution (ALE), eight independent *V.* *natriegens* parental lineages were evolved for 61 days in MOPS2 minimal medium (2.29% NaCl) containing 0.2% (13 mM) PCA as sole carbon source. The parental strain was streaked from cryostocks on LBv2 agar plates. Precultures were independently prepared from eight colonies as described in Supplementary Table 3. Cultures were grown in 0.4 mL PCA MOPS2 medium in 2.2-mL deep-well plates in a shaking incubator at 37 °C and 300 rpm. Every day, cultures were diluted 1:1000 following overnight growth (15 h) and 1:100 after 9 h of daytime growth, corresponding to an overall 1:100,000 dilution every 24 h. This regimen resulted in approximately 1,013 generations (Eq. 2). After 61 days, each line was streaked on LBv2 agar plates. Two individual colonies per evolved lineage were grown to late exponential phase in LBv2 liquid culture and stored at −80 °C as cryostocks. Genomic DNA was isolated from each evolved replicate as well as two replicates of the ancestral parental strain for whole genome sequencing with BGI (library type ≤ 800bp, sequencing platform DNBseq™).

Whole genome sequencing data were imported into Geneious Prime® 2025.2.2 as FASTQ files. Raw reads were aligned to the *V. natriegens* ATCC 14048 reference genome (NCBI accession CP009977/CP009978, modified to include the parental strain deletions Δdns, ΔVNP1 + 2), using the Geneious mapping tool configured with medium–low sensitivity. Mutations were then detected using the Geneious ‘Find Variations/SNPs’ feature, retaining variants present at frequencies greater than 50%. Variants present in the parental strain were classified as pre-existing background polymorphisms and excluded from downstream analysis of adaptive mutations.

2 $$\text{number of generations}={{\mathrm{log}}_{2}\left(\text{daily dilution factor}\right)*\text{ days}=\text{ log}}_{2}\left(\mathrm{100,000}\right)* 61=\mathrm{1,013}$$

### Extraction of genomic DNA

For genomic DNA extraction from *V.* *natriegens*, 4–5 mL of stationary phase liquid culture was centrifuged at maximum speed for 10 min, and the supernatant discarded. Cell pellets were resuspended in 300 µL PL2 buffer (10 mM Tris-HCl, 10 mM EDTA, 1% SDS), supplemented with 10 µL Monarch® RNase A (20 mg/mL, NEB T3018L). Samples were incubated at 65 °C at 400 rpm for 60 min for DNAse inactivation, followed by 37 °C at 400 rpm for 30 min for RNA digestion. Subsequently, samples were cooled to room temperature, before adding 300 µL PL3 buffer (2.8 M potassium acetate) and mixing by inversion. DNA was extracted with 300 µL chloroform:isoamyl alcohol (24:1), and the aqueous (top) phase was recovered after centrifugation for 10 min at 4 °C at maximum speed. DNA was precipitated by adding 350 µL isopropanol, followed by incubation at room temperature for 10 min. Samples were then centrifuged at maximum speed for 10 min, and the supernatant discarded. Precipitated genomic DNA was washed with 400 µL of 70% ethanol, which was removed after 1 min of centrifugation at maximum speed. Finally, genomic DNA was air-dried at 60 °C for 20–30 min, resuspended in 100 µL TE buffer, and stored at 4 °C. DNA quality and integrity were confirmed with agarose gel electrophoresis and the Qubit 4 fluorometer (Invitrogen; Thermo Fisher Scientific Q32850—Qubit™ dsDNA Quantification Assay Kit).

### Testing cell viability after extended exposure to oxidative stress

To compare revival from the viable-but-nonculturable (VBNC) state, the *V.* *natriegens* parental strain and all eight ALE lineages were streaked on LBv2 agar plates and incubated overnight at 37 °C, before storage at 4 °C. At days 1, 4, 8, 14, and 30 of cold storage, cells were re-streaked onto fresh LBv2 agar and incubated overnight at 37 °C to assess culturability (plates shown in Supplementary Fig. 3G).

The number of colony-forming units (CFU) of parental *V.* *natriegens* and ALE lineage 5, grown in MOPS2 minimal medium supplemented with 0.2% PCA, was quantified at 8, 12, 24, 36, and 48 h after inoculation (culture conditions in Supplementary Table 3). At each time point, cultures were diluted 1:10^5^ and 1:10⁶ in LBv2, and 100 µL of each dilution was plated on LBv2 agar using glass beads. Plates were incubated overnight at 37 °C, and CFUs were counted for each strain, time point, and dilution.

### Plasmid assembly, competent cells, and transformation

Plasmids generated in this study include level 1 constructs for PCA biosensing (pAF1_72_lux-operon_pPcaU, Fig. 3B) and NT-CRISPR plasmids used for deletion of PCA transporter candidates in *V. natriegens*. The level 1 plasmid for PHB production (P_Tet_-*phaBAPC*, Fig. 6A) was kindly provided by Politan et al. (2025). All plasmids and their assembly schemes are listed in Supplementary Table 4 (excluding NT-CRISPR plasmids). Oligonucleotides used to build NT-CRISPR and homology flank plasmids are provided in Supplementary Table 5–6. Newly constructed vectors were designed to remain compatible with the Vnat Collection (Faber et al. 2025), while employing the original Marburg Collection overhang architecture (Stukenberg et al. 2021). Plasmids were assembled using a Golden Gate Assembly (GGA) workflow adapted from Pryor et al. 2020. Standard reactions (15 μL) contained 15 fmol of each plasmid backbone, 45 fmol of PCR-derived fragments and/or 1 μL of 100 µM annealed oligonucleotides, 1.5 μL T4 ligase buffer (NEB B0202S), 0.75 μL Hi-T4 DNA ligase (400 U/μL, NEB M2622L), and either 0.5 μL BsaI-HFv2 (20 U/µL, NEB R3733L) for level 1 assemblies or 0.5 μL BsmBI-v2 (10 U/µL, NEB R0739L) for NT-CRISPR plasmid assemblies. BsaI-based reactions were cycled 30 times between 37 °C (1.5 min) and 16 °C (3 min). BsmBI-based reactions were cycled 60 times between 42 and 16 °C (5 min each). All reactions were finished with a 20-min digestion at 37 °C or 42 °C, respectively, followed by enzyme inactivation at 80 °C. Assembled plasmids were stored at 4 °C prior to transformation.

Chemically competent *V.* *natriegens* Δ*dns* and ΔVNP1 + 2 (parental strain), *E. coli* DH5α, and DH10β were generated following previously established protocols (Faber et al. 2025), inspired by Stukenberg et al. 2021 and Li 2011, with modifications. Heat-shock transformations were carried out using 5 μL of Golden Gate Assembly reaction for *E. coli* strains, whereas *V.* *natriegens* cells were transformed with 50–150 ng of purified plasmid DNA, as described previously (Faber et al. 2025). For plasmid extraction, we used the E.Z.N.A. Plasmid DNA Mini Kit (Omega Bio-tek, CustomScience D6943-03) as per manufacturer’s instructions.

### Genome engineering with NT-CRISPR

Genome edits in *V.* *natriegens* were introduced for the deletion of transporter candidates using the NT-CRISPR system following Stukenberg et al. 2022 with minor adaptations described in Faber et al. 2025. Guide RNAs (gRNAs) for NT-CRISPR plasmids (Supplementary Table 5) were designed in Geneious Prime 2025 .2.2 using the ‘Find CRISPR Sites’ function with algorithms by Doench et al. 2016. Complementary gRNA oligonucleotides (100 μM) were annealed by combining 1.5 μL of each oligonucleotide with 5 μL T4 DNA ligase buffer (NEB B0202S) in a 50 μL reaction, heating to 95 °C for 15 min, and cooling gradually to room temperature over 1 h. One microlitre of the annealed product was then inserted into the NT-CRISPR backbone plasmid (pST_116 from Stukenberg et al. 2022) via Golden Gate Assembly.

Linear transfer DNA (tDNA) was generated by splicing-by-overlap extension (SOE) PCR. Individual fragments were PCR amplified from the 5′ and 3′ homology arms of *V.* *natriegens* genomic DNA using Q5 High-Fidelity DNA polymerase (NEB M0491L) and primer sets listed in Supplementary Table 6. The composition of Q5 PCR master mix and PCR conditions are provided in Supplementary Tables 7–8. Amplified fragments were purified using the E.Z.N.A.® Cycle Pure Kit (Omega Bio-tek, CustomScience D6492-02) and fused by SOE PCR in a 48 µL reaction (Q5 reaction mix without primers), following the thermal cycler protocol in Supplementary Table 9. One microlitre of each primer (10 µM) was added to the SOE PCR product prior to final amplification using a standard Q5 PCR protocol. The resulting linear tDNA product was purified and 100 ng were used in NT-CRISPR genome editing. The NT-CRISPR workflow, plasmid curation, and verification of genetic modifications were performed as described in Faber et al. 2025, without modifications. Regions with genetic modifications were amplified and verified via Sanger sequencing, using the oligonucleotides listed in Supplementary Table 10.

### Quantification of cell growth

In plate reader assays, growth of *V.* *natriegens* was quantified in black, flat-bottom 96-well plates (Greiner 655096) using a BioTek Synergy HTX microplate reader. Pre-culture handling and inoculation procedures are listed in Supplementary Table 3. Each well contained 100 µL of culture and was measured without a lid. To reduce evaporation, peripheral wells and inter-well spaces were filled with water. Plates were incubated at 37 °C with continuous orbital shaking at 807 cpm (conditions in Supplementary Table 11). OD_600_ values were measured at 600 nm and luminescence was recorded across the full emission spectrum. OD_600_ and luminescence readings were blank corrected by subtracting the mean of ≥ 3 sterile medium wells matching the carbon source concentration at each time point. Growth rates were calculated from the exponential phase using linear regression in R.

Biomass accumulation in shake flasks was monitored in real time using a Cell Growth Quantifier (DOTS platform by Scientific Bioprocessing SBI). Cultivations were carried out in non-baffled 250-mL conical flasks containing either 20 or 100 mL of MOPS2 minimal medium supplemented with the respective carbon source. Biomass measurements showed substantial variability between flasks, likely caused by differences in glass surface texture and optical properties. This variability made cross-calibration between CGQ biomass signals and plate-reader OD₆₀₀ impractical. Therefore, CGQ biomass trajectories were aligned in R to an experiment internal reference point at 1–2 h after inoculation. This enabled comparison of relative growth dynamics between flasks rather than absolute OD_600_ values.

### Quantitative analysis of carbon source consumption and PHB production

An inducible PHB production module (P_Tet_–*phaBAPC*) was employed to uncouple PHB synthesis from growth phase and nutrient limitation. This construct, originally developed by Politan et al. (2025), encodes the core PHB biosynthetic enzymes PhaA, PhaB, and PhaC, enabling conversion of acetyl-CoA to PHB. The plasmid also carries the phasin PhaP, which facilitates intracellular PHB granule formation. The *V.* *natriegens* parental strain and ALE lineages were transformed with the P_Tet_–*phaBAPC* plasmid for PHB production from 0.1% PCA in optimised MOPS2 minimal medium (2.29% NaCl, pH 7.2). To ensure plasmid stability, all cultures were supplemented with kanamycin. Precultures were initiated from cryostocks and grown in 1 mL volumes using 2.2 mL 96-well deep-well plates (Sarstedt, 82.1972.002). Seed and production cultures were cultivated in 20 mL volumes in non-baffled 250-mL shake flasks (detailed in Supplementary Fig. 4 A). PHB production was induced by adding anhydrotetracycline (aTc) to a final concentration of 200 ng/mL. Aliquots of 1 mL were collected at 0, 4, 6, 8, and 12 h post-induction for quantification of PHB and residual PCA.

The primary goal of this proof-of-concept study was to demonstrate that *V.* *natriegens* can channel PCA-derived carbon into PHB. We therefore used BODIPY staining combined with flow cytometry and gravimetric cell dry weight (CDW) measurements—standard methodology for PHB detection and quantification of intracellular polyhydroxyalkanoate accumulation. For flow cytometry, 100 µL of culture were fixed by adding 12.1 μL ice-cold formaldehyde (35%) to a final concentration of 4%. Samples were incubated for 1 h at 4 °C, pelleted (10,000 × *g*, 10 min), the supernatant removed, resuspended in 100 µL fresh MOPS2 medium, and stored at 4 °C for up to 7 days. Prior to measurement, PHB granules were stained with BODIPY (Sigma-Aldrich 790389) and cells were counterstained with SYTO62 (Thermo Scientific S11344) according to the manufacturers’ instructions. A linear correlation between BODIPY signal and PHB content per cell dry weight for *V.* *natriegens* had previously been established by Politan et al. (2025). Stained samples were diluted in 0.5 × PBS containing 5 mM EDTA to obtain suitable cell densities. Flow cytometry was performed on a BD FACS Canto using standard laser/filter sets (BODIPY: 488 nm excitation, 530/30 nm emission; SYTO62: 633 nm excitation, 660/20 nm emission). Detector voltages for FSC, SSC, BODIPY, and SYTO62 were set to 450, 430, 420, and 603, respectively. Thresholds for FSC, SSC, and SYTO62 were set to 200, 200, and 5000, respectively. The flow rate was 12 µL/min and a minimum of 10,000 events were acquired per sample. Data processing and gating were performed in R using a custom workflow built on the flowCore package (v2.16.0).

Volumetric PHB titres were determined by converting PHB to crotonic acid via sulphuric acid digestion followed by HPLC analysis, as established by Politan et al. (2025). For each time point, 15–20 mL of culture was harvested (7200 × *g*, 5 min), washed once with optimised MOPS2 medium, and transferred to pre-weighed glass tubes. Cell dry weight (CDW) was obtained after drying pellets overnight at 60 °C to constant mass. Pellets were subjected to acidolysis by adding 1 mL of concentrated H₂SO₄ (98%) and incubating in boiling water for 1 h. After cooling to room temperature, reactions were diluted fourfold with ice-cold water and subsequently diluted 1:10 for analysis. Crotonic acid was quantified on an Agilent 1100 HPLC system with diode-array detection (DAD G1315B), using a REZEX ROA-Organic Acid H⁺ (8%) column (300 × 7.8 mm) and 3 mM H₂SO₄ as the mobile phase at 0.5 mL/min. Injections of 20 µL were run at 60 °C, and absorbance at 210 nm (bandwidth 4 nm) was used for quantification. Peak areas were processed in ChromatographR against crotonic acid standards (Sigma-Aldrich 113018-500G) prepared in water (Supplementary Fig. 4B). PHB concentrations were calculated from crotonic acid using a 0.91 molar conversion factor (Politan et al. 2025).

Extracellular PCA concentrations were quantified from culture supernatant to determine PCA consumption during PHB production (above) and PCA uptake rates (Supplementary Table 3 and Supplementary Table 13). For each time point, 900 µL of culture was centrifuged (10,000 × *g*, 1 min), and clarified supernatant was used directly or diluted 1:10 for the 0 h samples. PCA concentrations were measured on an Agilent 1260 Infinity II HPLC system equipped with a diode-array detector (DAD G7117C). Chromatographic separation was achieved on a ReproSil XR 120 C18 column (250 × 4.6 mm, 5 µm, part number rx15.9e.s2546) using a gradient elution of acetonitrile and 0.1% trifluoroacetic acid (gradient profile in Supplementary Table 12). The method operated at 1 mL/min with a 10 µL injection volume at room temperature. Absorbance at 240 nm (8 nm bandwidth) was used for quantification, and peak areas were analysed in ChromatographR (Bass, E., 2023, version 0.7.1) using standard calibration curves obtained from PCA (Sigma-Aldrich 37,580-25G-F) standards prepared in water (Supplementary Fig. 4 C).

### Metabolic modelling of PCA metabolism in *V. natriegens*

The genome-scale metabolic model (GSMM) by Coppens et al. 2023 (iLC858) was used for in silico analysis of PCA metabolism in *V. natriegens*. A recently reported, updated version of the model (Politan et al. 2025) was used as the starting point. Manual curation was required to simulate growth on PCA as sole carbon source. Genes and metabolic pathways related to PCA uptake and metabolism were identified based on KEGG annotations and NCBI genome records for *V. natriegens* (NZ_CP009977, NZ_CP009978). Model curation included the addition of (i) exchange and transport reactions for PCA, (ii) all reactions in the 4,5-meta cleavage pathway, and (iii) the reaction catalysed by 3-dehydroshikimate dehydratase. Where needed, missing reaction-associated metabolites were also added. The curated GSMM (iLC858_FritzLab_v2) can be accessed at https://github.com/FritzLabUWA/Vnat_GSMM.

Following model curation, flux balance analysis (FBA) was conducted to predict growth rate, biomass yield, and carbon flux distribution during growth on PCA as sole carbon source. The PCA uptake rate was constrained using experimentally derived values (Supplementary Table 13). Biomass formation was set as the objective, and uptake rates for all other carbon substrates were set to 0 mmol/g_CDW_/h. All GSMM curation and analyses were performed using the COBRApy package in Python.

### Computational structure prediction for OxyR

The amino acid sequence of *V.* *natriegens* OxyR (Accession: WP_014233154) was retrieved from NCBI and a 3D-model was constructed using Phyre2.2, I-TASSER, and AlphaFold 3 (Abramson et al. 2024). All three tools produced highly similar models, indicating strong agreement in the predicted protein conformation. AlphaFold 3 was selected for subsequent structural analyses, given its capability to model multi-protein complexes and protein-DNA interactions. Mutation locations were visualised in PyMOL.

To construct a consensus binding motif for *V.* *natriegens*’ OxyR, we first compiled predicted OxyR binding sites from *Vibrio cholerae* (from Wei et al. 2012) to generate a clade-level consensus motif. A list of genes putatively regulated by OxyR was assembled and used to search for homologs in the *V. natriegens* genome. Seven homologs were identified and upstream regions were scanned for the consensus OxyR binding motif using FIMO (Grant et al. 2011). All seven regions yielded significant matches (*p* < 0.01), with *q*-values varying across sites. The best match had a *q*-value of 0.00705 and was found upstream of *oxyR* itself (PN96_RS15030).

## Results

### Endogenous PCA metabolism in *V. natriegens*

**Fig. 2:**
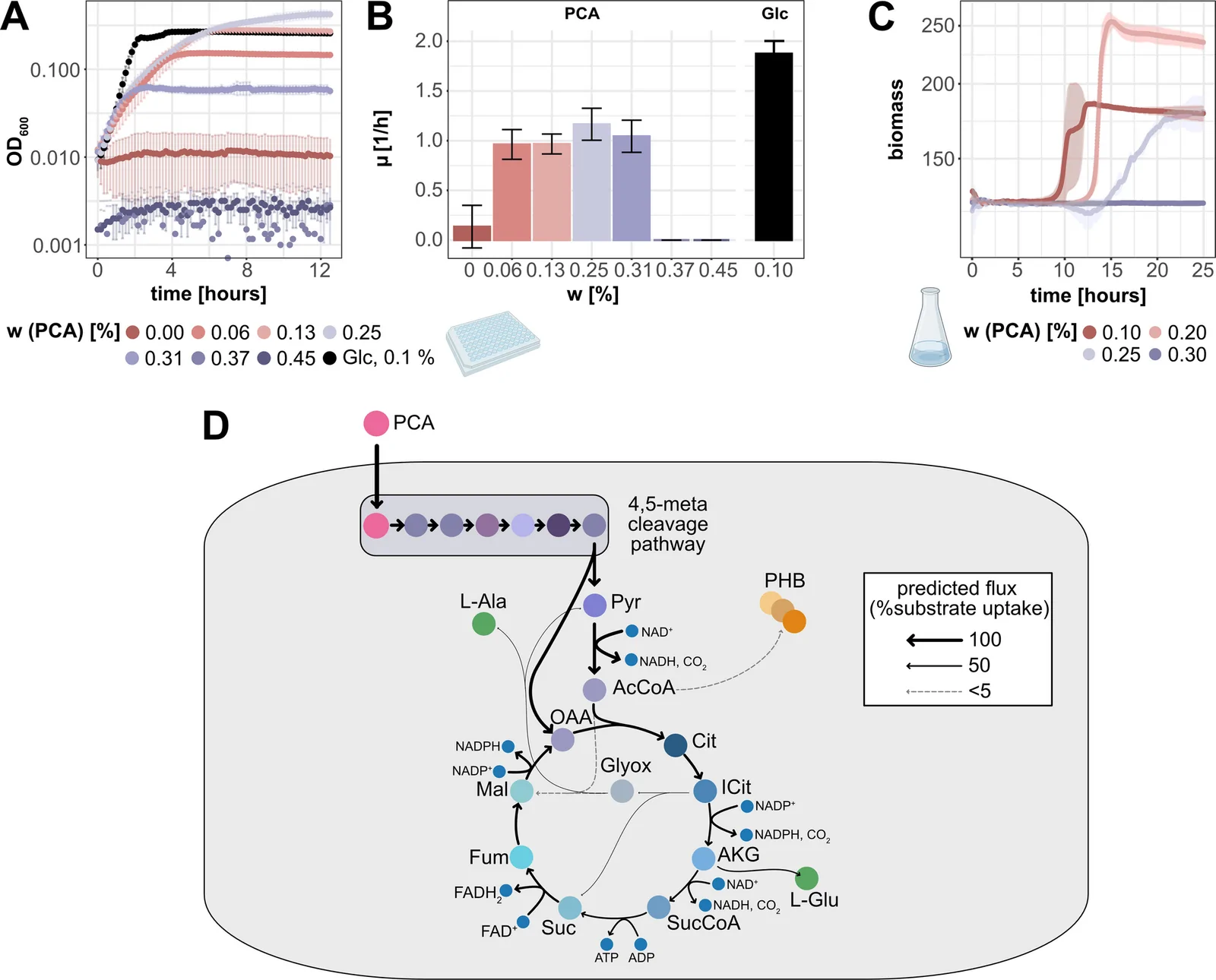
Characterisation of PCA catabolism in *V. natriegens*. **A** Growth curves and **B** growth rates μ during growth on PCA as the sole carbon source in MOPS2 minimal medium in 96-well plate cultivation. Data represents the mean of three biological replicates in two independent experiments. **C** Growth curves of the parental strain on different PCA concentrations in 20 mL MOPS2 minimal media in 250-mL-non-baffled flasks. **D** Schematic of fluxes through central carbon metabolism during growth on PCA as sole carbon source. For each reaction, the predicted flux relative to the specific substrate uptake rate is indicated by the line width of the corresponding reaction arrow. **D**, **E** Based on data in Supplementary Table 13

*V. natriegens* utilises protocatechuic acid (PCA) as the sole carbon source in defined minimal medium, achieving a maximum specific growth rate of ~ 1.15 h^−1^ at 0.25% (w/v) PCA in plate reader experiments (Fig. 2A, B). Increasing the PCA concentration up to 0.25% improved both growth rate and final cell density. However, higher PCA levels proved toxic: growth was inhibited above ~ 0.3% PCA and ceased completely at ~ 0.37% in microplate cultures. Similarly, in shake flasks, even 0.25% PCA reduced the final biomass and lengthened the lag phase, and 0.30% PCA completely prevented growth (Fig. 2C). We attribute the longer lag phase and higher PCA toxicity to the greater oxygen transfer rates in shake flask cultures, as discussed further below. Notably, *V.* *natriegens* grew more slowly on PCA (a 7-carbon aromatic) than on an equimolar amount of glucose (6-carbons), indicating lower PCA utilisation efficiency despite its greater carbon content.

**Fig. 3:**
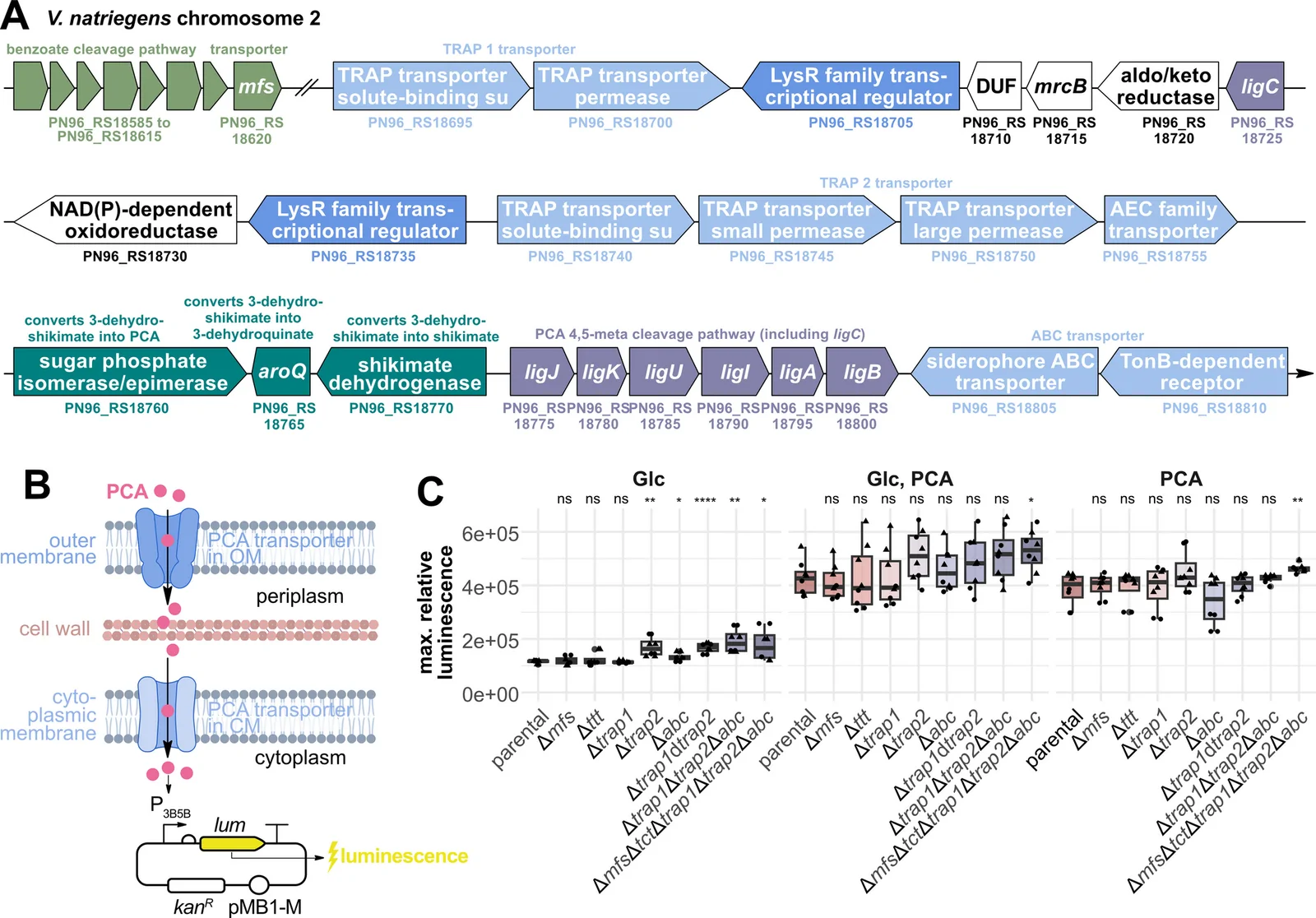
Endogenous PCA transporters in *V.* *natriegens*. **A** 4,5-Meta cleavage pathway and PCA transporter candidates are encoded in a gene cluster on chromosome 2 (details on PCA transporter candidates in Supplementary Table 14). su – subunit. **B** Proposed uptake of PCA over the outer and inner membrane. Intracellular PCA was detected using a plasmid-based PCA-biosensor comprising the P_3B5B_ PCA-inducible promoter upstream of a luminescence reporter cassette. **C** Relative luminescence signal from strains with knockouts of PCA transporter candidates, carrying the plasmid-based biosensor. OD_600_ and luminescence were measured in 96-well plates. Shapes represent the two biological replicates, measured on two independent days. Statistical analysis was performed with an unpaired two-way *t*-test (*p < 0.05, **p < 0.01, ***p < 0.001, ****p < 0.0001)

Genomic analysis revealed that *V. natriegens* degrades PCA via the 4,5-meta cleavage pathway. A contiguous cluster of *ligJ*, *ligK*, *ligU*, *ligA*, and *ligB* genes (locus tags PN96_RS18775–PN96_RS18800) on chromosome II encodes the enzymes of this pathway, with a separate *ligC* gene (PN96_RS18725) located just upstream (Fig. 3A). Deletion of *ligK* abolished growth on PCA (Hädrich et al. 2025b). An adjacent gene (PN96_RS18760) encodes a putative 3-dehydroshikimate dehydratase (also annotated as sugar phosphate isomerase/epimerase) that converts the shikimate-pathway intermediate 3-dehydroshikimate into PCA (Huccetogullari et al. 2019; Weaver And Herrmann 1997). Additionally, *V.* *natriegens* harbours a *pobA* gene whose product likely interconverts PCA and 4-hydroxybenzoate (a precursor for ubiquinone and other cofactors; Supplementary Fig. 1 A). In contrast, only isolated genes from the alternative 2,3-meta and ortho PCA degradation routes are present in the genome (e.g. *praE*, *praF*, *praG*, *pcaH*; *pcaC*, *pcaD*, *pcaF*), and these pathways are incomplete (Fig. 1B). Taken together, this reveals that *V.* *natriegens* exclusively relies on the complete 4,5-meta cleavage pathway.

To validate this pathway’s function, we incorporated the 4,5-meta cleavage pathway reactions into the *V. natriegens* genome-scale metabolic model *i*LC858 (Coppens et al. 2023) and simulated growth on PCA in silico. Shake-flask experiments yielded a specific PCA uptake rate of 12.2 ± 0.9 mmolPCA gCDW^−1^ h^−1^ (Supplementary Fig. 1B, Supplementary Table 13). This value was used to constrain the model, which predicted a growth rate of ~ 1.16 h^−1^ and a biomass yield of 0.095 gCDW per mmol PCA. These values closely match the experimental data (1.19 ± 0.04 h^−1^ and 0.10 ± 0.01 gCDW/mmol; Supplementary Fig. 1C-D). The in silico flux distribution confirmed that PCA is optimally catabolised through the 4,5-meta cleavage pathway, yielding pyruvate and oxaloacetate that enter central metabolism via the TCA cycle (Fig. 2E). Consistent with the model’s objective function (maximal biomass formation), no flux was predicted through *V. natriegens’* native PHB biosynthetic pathway.

### PCA uptake in *V. natriegens* by an unidentified transporter

At neutral pH, aromatic acids such as benzoate, terephthalate, and PCA (pKa 4.48) are largely dissociated and cannot freely diffuse across the cell membrane (Collier et al. 1997; Harwood et al. 1994). Depending on concentration, dedicated transporters are therefore necessary for their uptake. In many bacteria, the deletion of such transporter genes severely impairs or abolishes growth on the corresponding aromatic substrates (Collier et al. 1997; Harwood et al. 1994; Hosaka et al. 2013; Sasoh et al. 2006). We therefore hypothesised that *V.* *natriegens* encodes one or more specific transport systems for PCA uptake under our growth conditions (MOPS2 minimal medium at pH 7.2). To identify the responsible transporter, we constructed eight *V. natriegens* strains carrying deletions in candidate transporter genes (individual knockouts and combinations) and measured intracellular PCA levels using a plasmid-based luminescent PCA biosensor (P_3B5B_ promoter driving a luciferase reporter; Fig. 3B). Five candidate transport loci were selected for knockout due to homology to known aromatic acid transporters or proximity to the PCA 4,5-cleavage pathway genes on chromosome 2 (Supplementary Table 14, Fig. 3A). These included an MFS-type aromatic acid symporter (PN96_RS18620), a tripartite tricarboxylate transporter (TTT, PN96_RS05400–PN96_RS05410), two TRAP transporters (PN96_RS18695–18,700 and PN96_RS18740–18755), and a TonB-dependent siderophore-type ABC transporter (PN96_RS18805–18810).

None of the deletions, however, produced a detectable defect in PCA uptake or growth in MOPS minimal medium with 0.1% PCA at pH 7.2. All knockout strains exhibited luminescence signals (intracellular PCA levels) comparable to the parental strain (Fig. 3C). This held for each single-gene knockout as well as the strain lacking all five candidate transporters. No significant differences were observed in growth rate, final cell density, or PCA consumption among the mutants (Supplementary Fig. 2, Supplementary Table 13). Minimal variability across biological replicates (likely due to plate position effects), underscored the robustness of these results. These findings indicate that the five targeted transporters are not essential for PCA uptake in *V. natriegens* at the tested PCA concentrations, neither individually nor in combination. PCA may instead enter through an as-yet unidentified redundant transporter, or at 0.1% PCA passive diffusion of the protonated acid may provide sufficient intracellular carbon for growth. Either scenario points to a robust PCA uptake capability in *V. natriegens*.

### Adaptive laboratory evolution of *V. natriegens* on PCA

**Fig. 4:**
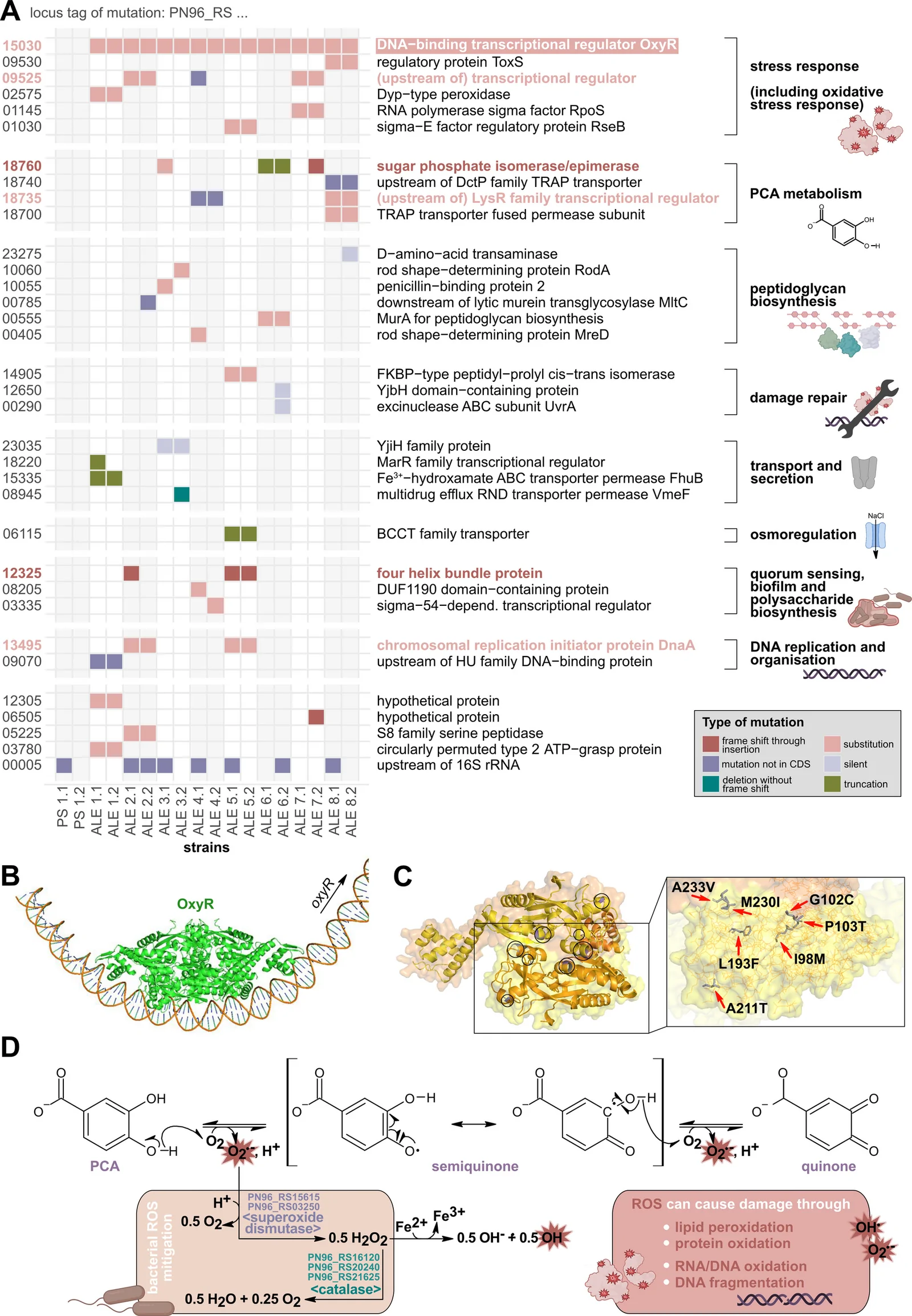
Adaptive laboratory evolution (ALE) of *V.* *natriegens* on PCA. **A** Mutations accumulated in eight independent lineages after exposure to 0.2% PCA as sole carbon source in MOPS2 minimal medium (2.29% NaCl, pH 7.2) over 61 days compared with the parental strain (PS). Mutation types are indicated by colours, with coloured text highlighting mutations in more than one lineage, see Supplementary Table 15 for details. CM – cytoplasmic membrane. **B** Model of OxyR in its reduced state binding to the OxyR-recognition site (repetitions of the consensus sequence ATAACACTAAACGAT) upstream of *oxyR*. **C** OxyR structure with locations of the ALE mutations highlighted. Mutations localise near functionally important residues, involved in the binding of hydrogen peroxide (near, e.g. T100, G197, H198) or the structural change after oxidation that modifies OxyR’s DNA-binding affinity (e.g. C199, C208). The predicted structure was created using AlphaFold 3 (Abramson et al. 2024) and rendered using PyMOL. **D** Proposed reaction of PCA (a quinol) with oxygen under physiological conditions to form a quinone and superoxide (O_2_·^−^)—a reactive oxygen species (ROS). *V.* *natriegens* mitigates the production of ROS, including the conversion of O_2_·^−^ into hydrogen peroxide (H_2_O_2_) by the superoxide dismutase (SOD) and the breakdown of H_2_O_2_ by the catalase-peroxidase (KatG). However, Fenton chemistry reduces H_2_O_2_ to form hydroxyl radicals (**·**OH) in the presence of Fe^2+^, another highly reactive ROS that causes cell damage. Inspired by (Ajiboye et al. 2017; Galano and Pérez-González 2012; Imlay et al. 1988; Li et al. 2011; Lushchak 2001; Seixas et al. 2022; Tan et al. 2019)

To optimise growth on PCA as a sole carbon source, we subjected eight independent parental *V.* *natriegens* lineages to adaptive laboratory evolution (ALE) on 0.2% PCA in minimal medium for 61 days (~ 1013 generations). Whole-genome sequencing of two colonies per lineage revealed the accumulation of a distinct set of mutations (5–7 genomic loci per lineage), affecting a total of 30 coding sequences and 6 intergenic regions (Fig. 4A). The number of affected genomic loci was comparable to that observed in *V.* *natriegens* strains evolved on acetate over a comparable evolutionary timescale (Politan et al. 2025). The mutations spanned eleven functional categories, with many targeting oxidative stress responses, PCA catabolism, cell envelope biosynthesis/repair, DNA maintenance, and other stress-related functions (Fig. 4A; see Supplementary Table 15 for full mutation list). Mutations were classified as convergent (present in two or more independent lineages) or lineage-specific (present in a single lineage). Lineage-specific mutations may reflect neutral fixation events during ~ 1013 generations of evolution rather than adaptive variants, and are interpreted with appropriate caution throughout.

### OxyR: a universal target of PCA-induced oxidative stress

The most common convergent target was *oxyR* (PN96_RS15030), mutated in all eight evolved lineages, with six distinct non-synonymous substitutions (Fig. 4A, C). OxyR is a conserved redox-sensitive transcriptional regulator in bacteria (Chiang and Schellhorn 2012) and a known hotspot for adaptive mutations under oxidative stress in *V.* *natriegens* (Anand et al. 2020). In its reduced form (absence of reactive oxygen species (ROS)), OxyR binds to its target promoters and represses expression of antioxidant genes (Toledano et al. 1994). Upon oxidation by peroxides or superoxide, OxyR undergoes a conformational change that activates the expression of ROS-detoxifying enzymes (Anand et al. 2020; Chiang and Schellhorn 2012; Storz et al. 1990).

We generated a structural model of *V.* *natriegens* OxyR, which formed a plausible tetrameric structure similar to OxyR from *Pseudomonas aeruginosa* and *Vibrio vulnificus* (ipTM = 0.57, pTM = 0.63; Supplementary Fig. 3 A) (Jo et al. 2015a, b; Pedre et al. 2018). The model bound a predicted OxyR consensus DNA site upstream of *oxyR* (5′-ATAACACTAAACGAT-3′), consistent with its autoregulatory dual function (ipTM = 0.55, pTM = 0.61, Fig. 4B). Introducing the six ALE mutations into the model reduced predicted structural stability (ipTM = 0.35, pTM = 0.45, Supplementary Fig. 3B-C), and the substitutions clustered near residues important for H_2_O_2_ sensing (e.g. Thr100, His198) and redox-dependent DNA-binding (e.g. Cys208) (Supplementary Fig. 3D–E). Similar adaptive mutations in *oxyR* have been reported under oxidative stress in *V.* *natriegens* (Anand et al. 2020), suggesting that the ALE mutations render OxyR more readily activated (or constitutively active). This enhanced OxyR-driven response probably mitigates PCA-induced ROS damage more effectively.

Consistent with earlier reports (Ajiboye et al. 2017), our results confirm that PCA imposes oxidative stress as the primary selection pressure, driving *oxyR* as a convergent adaptive target in every evolved lineage. Convergent mutation of the same locus across all eight independent populations constitutes strong evidence for functional relevance. Re-introduction of individual ALE mutations into the parental strain background would provide mechanistic validation, and we propose this as a priority for future work.

### Other mutations in the intra- and extracellular stress responses

Beyond OxyR, several additional (oxidative) stress regulators were mutated in the ALE lineages. One lineage (ALE7) acquired an amino acid substitution in the alternative *σ* factor *σ*^S^ (RpoS) (I123N in PN96_RS01145; Fig. 4A). RpoS controls hundreds of stress-response genes (including stationary-phase catalase-peroxidase enzymes) in bacteria (Battesti et al. 2011; Lange And Hengge-Aronis 1991; Lévi-Meyrueis et al. 2015). Notably, the I123N variant in ALE7 is identical to an *rpoS* polymorphism found in certain laboratory strains of *V.* *natriegens* that is known to reduce catalase activity and H_2_O_2_ tolerance. Our sequencing data show that the parental progenitor strain DSM 759 carries the ancestral (fully functional) RpoS sequence (E71, I123) (Glasgo 2024). The evolved I123N mutation therefore likely impaired RpoS regulatory function in ALE7, potentially leading to reduced catalase-peroxidase activity and H₂O₂ degradation capacity (Glasgo 2024).

Another recurrent adaptation occurred in a dye-decolorizing peroxidase (Dyp) (PN96_RS02575) that decomposes H_2_O_2_ (Glasgo 2024). Both ALE1 colonies carry the same S167F substitution, which may enhance enzyme activity or stability under elevated H_2_O_2_ levels. We also observed a truncation of a MarR family transcriptional regulator (PN96_RS18220) due to a premature stop codon in ALE1.1 (Fig. 4A). MarR homologs typically repress genes involved in multidrug/xenobiotic resistance and oxidative stress; this repressor function is relieved upon oxidation of key cysteine residues or ligand binding (Alves et al. 2021; Grove 2013).Together, the RpoS, Dyp, and MarR mutations suggest that ALE lineages might reinforce intracellular oxidative stress defences through multiple pathways beyond OxyR.

In addition to these intracellular targets, ALE revealed adaptive mutations in extracytoplasmic stress-sensing pathways. Half of the evolved lineages (4 out of 8) acquired mutations in the *toxS* locus (PN96_RS09530) or its vicinity, making this the second most frequent adaptive target after *oxyR*. ALE lineage 8 harboured a missense mutation in the ToxS protein itself (G164D), while lineages 2, 4.1, and 7 carried mutations in a regulatory gene immediately upstream of *toxS* (e.g. T91A, S281F in PN96_RS09525). In *Vibrio* species, the ToxRS two-component system is known not only for virulence regulation (DiRita And Mekalanos 1991) but also for sensing environmental stresses (such as bile salts, low pH, and detergents) and triggering survival responses (Gubensäk et al. 2023; Whitaker et al. 2012) via the extracytoplasmic function σ factor σ^E^ (RpoE) (De Las Peñas et al. 1997; Dou et al. 2018; Mathur et al. 2007; Mecsas et al. 1993; Whitaker et al. 2012). Additionally, one lineage (ALE5) acquired a mutation in *rseB* (PN96_RS01030; A70P), a negative regulator of the σ^E^ pathway (Fig. 4A) (De Las Peñas et al. 1997). Further work is needed to confirm the specific signals and regulon of the ToxRS system in *V.* *natriegens*.

Overall, these evolutionary adaptations to PCA involved a network of regulators and stress-mitigation mechanisms. Beyond OxyR-, RpoS-, MarR-, ToxRS-, and σ^E^-mediated regulatory changes, the ALE lineages accrued mutations in cellular repair and maintenance pathways. Different lineages had mutations in genes related to peptidoglycan biosynthesis, protein quality control, and DNA repair (see Supplementary Information). These likely contribute to tolerance of ROS-induced cellular damage. However, most of these occurred in single clones within a lineage, suggesting they have not yet reached fixation and represent secondary or strain-specific adaptations. In summary, *V.* *natriegens* adapted to growth on PCA by strengthening rapid, early-phase oxidative stress responses and bolstering damage repair systems, thereby countering the multi-faceted toxicity of PCA.

### Media optimization: replacing Fe^2+^ to reduce ROS stress

**Fig. 5:**
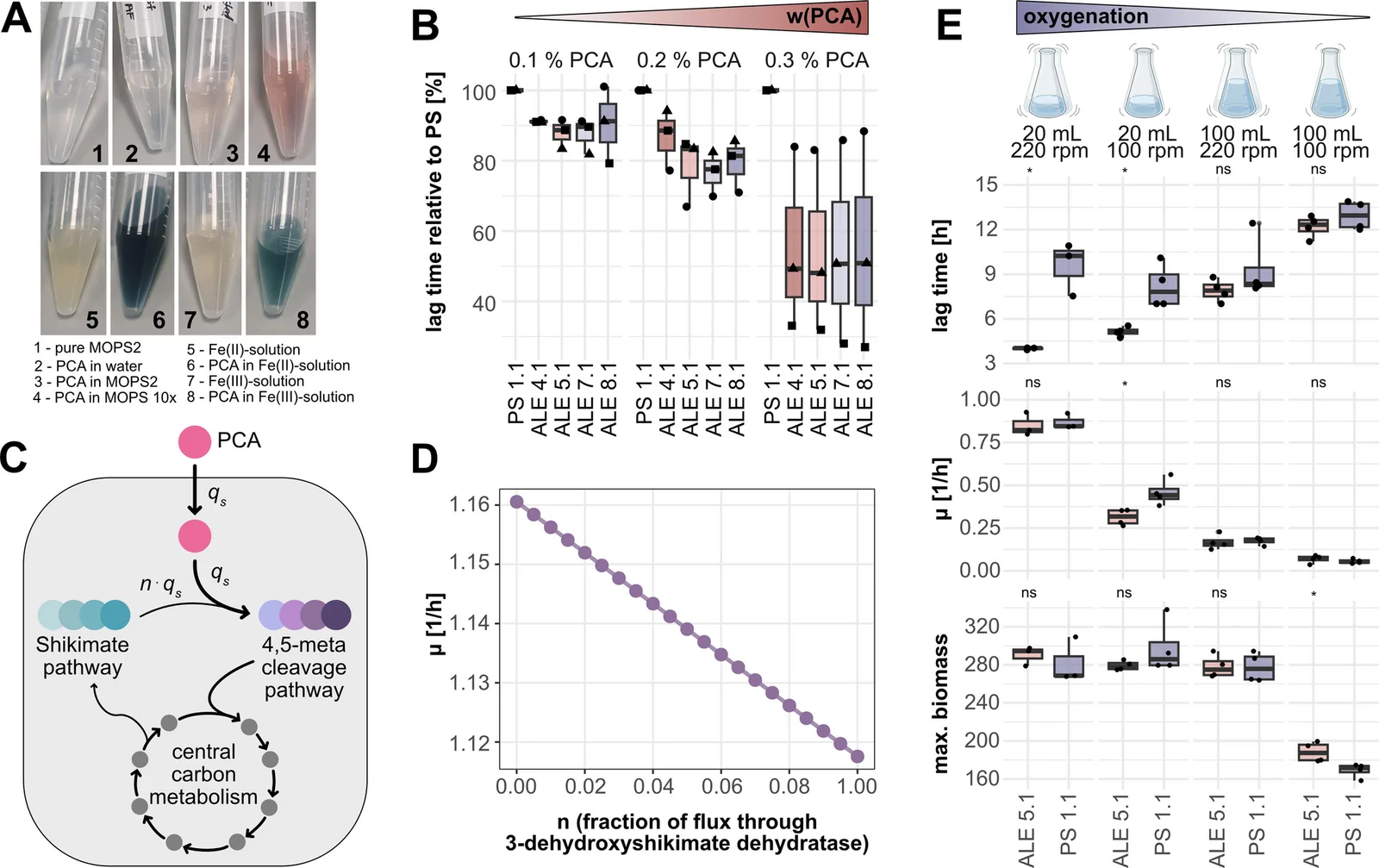
Phenotypic characterisation of ALE lineages. **A** Colour comparison for PCA dissolved in water, MOPS2 minimal medium, MOPS2 10 × stock solution, Fe^2+^- and Fe^3+^-solutions in contrast to pure MOPS2 and iron solutions. **B** Lag time (defined as time to 0.5 × maximum OD_600_) of ALE lineages relative to the parental strain (given in %) during growth on various concentrations of PCA in optimised MOPS2 minimal medium in shake-flask culture (20 mL in 250-mL non-baffled flasks). Data was obtained from three independent technical replicates. **C** Schematic of PCA flux modelling. PCA is transported into the cell and enters the 4,5-meta cleavage pathway at a rate equivalent to *q*_*s*_ (specific substrate uptake rate). To assess the effects of routing carbon flux through 3-dehydroshikimate dehydratase, a minimal flux equivalent to *n*·*q*_*s*_ is imposed on this reaction, where *n*·*q*_*s*_ ≤ *q*_*s*_. **D** Growth rates obtained via iterative FBA with increasing values of *n*, i.e. increasing the minimal flux routed through 3-dehydroshikimate dehydratase. **E** Lag time (time to reach 0.5 × maximum OD_600_), growth rate, and maximum OD_600_ for parental strain (PS) and best-performing ALE lineage (ALE5) growing in MOPS2 minimal medium with 0.2% PCA in 250-mL-non-baffled flasks with different oxygenation levels, dependent on shaking speed [rpm] and culture volume [mL]. Data were obtained from four independent technical replicates. Statistical comparisons: unpaired two-tailed *t*-test (*p < 0.05, **p < 0.01, ***p < 0.001, ****p < 0.0001). **B**, **E** show independent experiments conducted weeks apart under identical conditions (20 mL, 220 rpm); differences reflect expected variability between repetitions

Our understanding of PCA-induced ROS toxicity enabled a targeted improvement of the growth medium. We observed that PCA solutions underwent a dramatic colour change in the presence of ferrous (Fe^2+^) iron: in MOPS medium (which contains FeSO_4_), dissolved PCA turned from colourless to pink, and in concentrated Fe^2+^ solutions PCA even turned dark blue (Fig. 5A), indicative of rapid PCA autoxidation. No such colour change occurred with other media components (Supplementary Fig. 3 F). We therefore hypothesised that Fe^2+^ was catalysing Fenton reactions with PCA, generating excess ROS (Fig. 4D) (Imlay et al. 1988). To test this, we modified the medium by substituting Fe^2+^ with Fe^3+^ (ferric iron). Iron speciation in aqueous media is dynamic: Fe^2^⁺ and Fe^3^⁺ interconvert depending on redox potential, pH, and the presence of chelating agents. However, free Fe^2^⁺ is not simply interchangeable with Fe^3^⁺ at pH 7.2 in an aerated medium. Ferrous iron is rapidly oxidised by dissolved oxygen at circumneutral pH, while Fe^3^⁺ often forms poorly soluble ferric precipitates unless it is stabilised by chelators (Pham et al. 2006; Stumm And Lee 1961). We argue that replacing Fe^2^⁺ with Fe^3^⁺ might circumvent a transient period of more reactive ferrous iron, while directly supplying Fe^3^⁺ in a neutral medium allows ferric ions to be bound more strongly by chelators such as citrate and PCA (Li et al. 2011). However, PCA itself can reduce Fe^3^⁺ back to Fe^2^⁺, regenerating Fenton-reactive iron. Thus, Fe^3^⁺ substitution would not eliminate iron-mediated ROS production, but would shift its kinetics, predicting a partial but consistent improvement in growth.

Indeed, *V.* *natriegens* grew with Fe^3+^ as the sole iron source, and in the Fe^3+^-medium the parental strain tolerated PCA concentrations up to 0.30% in shake flasks—higher than the ~ 0.25% limit in the original Fe^2+^-containing medium (Fig. 5B vs. Fig. 2C). Thus, minimising Fe^2+^ availability in the medium mitigated ROS generation from PCA and extended the usable PCA concentration range. Interestingly, one ALE lineage (ALE1) had independently evolved a truncation in a Fe^3+^-hydroxamate uptake protein (FhuB, an ABC siderophore transporter; Fig. 4A), which could reduce iron import into the cell (Al Shaer et al. 2020). This would likewise diminish intracellular Fe^2+^ and limit Fenton-mediated hydroxyl radical formation. The convergence of this spontaneous adaptation with our media optimization underscores the importance of iron-mediated ROS in PCA toxicity. In practical terms, supplying Fe^3+^ instead of Fe^2+^ is an effective means of reducing oxidative stress when using PCA (or other ROS-generating compounds) in culture.

### Adaptive mutations affect PCA catabolism and transport

Several mutations affect genes involved in PCA metabolism and transport. Three independent ALE lineages acquired loss-of-function mutations in the 3-dehydroshikimate dehydratase enzyme (PN96_RS18760) that converts 3-dehydroshikimate into PCA (Bentley And Haslam 1990) (Supplementary Fig. 1 A). These adaptive changes include missense mutations (e.g. G68W, P70L) and null mutations (a stop codon and a frameshift) (Fig. 4A). Disabling this enzyme could conserve 3-dehydroshikimate, an intermediate required for aromatic amino acid and folate biosynthesis, rather than diverting it to PCA already supplied exogenously. The evolved strains may have decoupled carbon flux from the shikimate pathway to the PCA 4,5-meta cleavage pathway. We tested this hypothesis in silico using a curated genome-scale metabolic model. When a minimal flux was imposed on the 3-dehydroshikimate dehydratase step (simulating partial enzyme activity), the model predicted a lower growth rate; conversely, the predicted growth rate was maximised when flux through this step was zero (Fig. 5C, D). This is consistent with the observation that ALE strains with PN96_RS18760 outcompeted the parental *V.* *natriegens* strain during serial passaging on PCA by eliminating this futile flux.

In ALE lineage 8, we also found mutations in two TRAP transporters located adjacent to the PCA degradation operon (Fig. 3A). One is a nucleotide substitution in the promoter region of a TRAP transporter gene (PN96_RS18740); the other is a coding mutation in the permease subunit of a second TRAP transporter (PN96_RS18700). TRAP transporters are common in marine bacteria (Rosa et al. 2018) and are known to import aromatic acids in other species (Chae And Zylstra 2006; Salmon et al. 2013). A third mutation hotspot involves a LysR-family regulator gene near the PCA catabolic cluster, which might be involved in its regulation. ALE lineages 4 and 8 each accumulated a mutation in the *lysR* coding sequence or its upstream region. In sum, the ALE process selected for mutations in metabolic enzymes, transporters, and regulators that together might optimise flux through the PCA 4,5-meta-cleavage pathway, increasing growth efficiency on PCA.

### Phenotypic differences between parental and ALE lineages

Four colonies of four different ALE lineages (ALE 4.1, 5.1, 7.1, and 8.1) were selected from the panel of sixteen sequenced colonies for phenotype analysis. Selection was based on three criteria: (i) superior growth performance on high concentrations of PCA compared to the parental strain (Fig. 5B); (ii) representation of distinct mutational profiles (Fig. 4A)—with mutations of genes related to PCA metabolism in ALE 4.1 and 8.1 and mutations in genes associated with cellular stress response including oxidative stress like *rseB*, *rpoS*, and *toxS* in ALE 5.1, 7.1, and 8.1, respectively; (iii) experimental capacity constraints of the CGQ measurement setup, which limited the number of strains that could be analysed simultaneously.

We examined how the accumulated genetic adaptations translated into improved growth phenotypes under PCA stress. At low PCA concentrations (0.1% w/v), all ALE lineages grew similarly to the parental strain (Fig. 5B). However, at higher PCA levels (0.2–0.3%, which impose greater ROS stress), the ALE lineages reached mid-exponential phase faster than the parental strain. This advantage reflected a shorter lag phase before exponential growth, rather than a higher exponential growth rate. The best-performing strain (ALE 5.1) initiated growth after a much shorter lag, whereas the parental strain experienced a prolonged delay on 0.3% PCA. We attribute the high variation in lag phase between technical replicates to the threshold-like toxicity of PCA at 0.3%: small differences in inoculum density, initial OD, or the age of PCA-containing medium, and thus PCA toxicity, can strongly affect growth kinetics at this concentration. A similar pattern emerged under increased aeration (which accelerates PCA oxidation and ROS generation): with more intense oxidative stress, ALE 5.1 began growing sooner than the parental strain, even though maximum growth rates and biomass yields remained comparable (Fig. 5E). These results indicate that the evolved strains overcome the initial inhibitory effects of PCA more rapidly, likely through their enhanced oxidative stress responses, thereby entering exponential growth sooner.

By contrast, we found little difference in long-term survival or stationary phase tolerance between the parental strain and ALE lineages. All retained similar viability after extended cold exposure (4 °C storage leading to a viable but non-culturable (VBNC) state) and during 48 h growth in the presence of PCA (no significant divergence in CFU over time; see Supplementary Fig. 3G–H). Thus, the primary benefit of adaptation was improved growth initiation under PCA stress, rather than increased persistence or survival under long-term stress. This is consistent with the mutations observed: the ALE lineages strengthened immediate-early oxidative stress defences (e.g. OxyR, ToxRS, σ^E^) rather than mechanisms of stationary-phase stress tolerance (e.g. RpoS). In summary, adaptive evolution on PCA yielded *V.* *natriegens* lineages that grow faster in the face of PCA’s oxidative challenge, while their overall growth capacity and survival at steady-state remain similar to the parental strain.

### PHB production from PCA with adaptively evolved *V. natriegens*

**Fig. 6:**
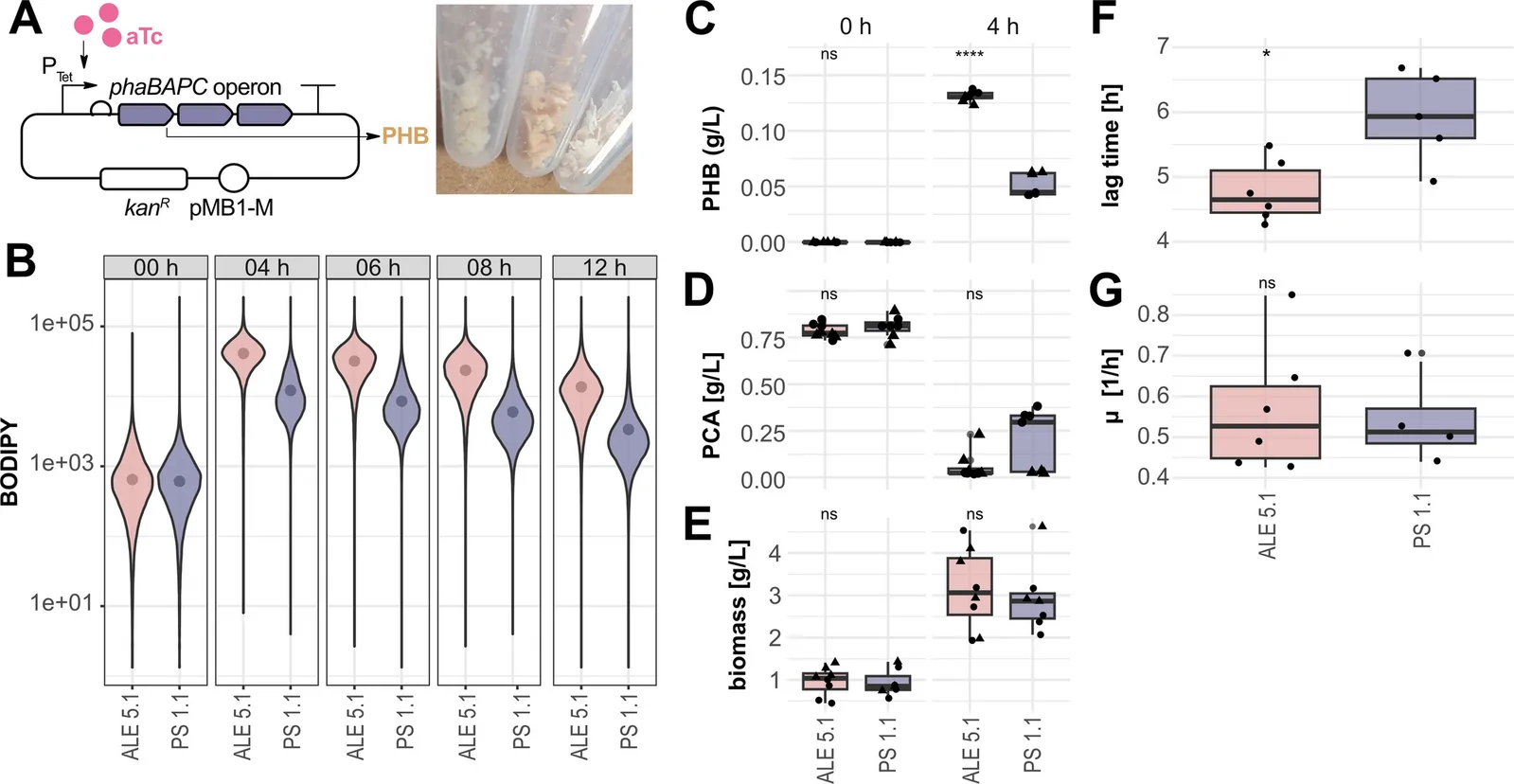
Production of PHB from PCA in *V.* *natriegens*. **A** Plasmid design for PHB production. The native *V.* *natriegens phaBAPC* operon is expressed under the aTc-inducible P_Tet_ promoter. Acetyl-CoA is condensed by PhaA, reduced by PhaB, and polymerised by PhaC to produce PHB (Politan et al. 2025), which accumulates in intracellular granules stabilised by the phasin PhaP and recovered by organic solvent extraction. **B** BODIPY fluorescence at different time points after induction of PHB production in the parental strain (PS) and ALE 5.1 carrying the aTc-inducible *phaBAPC* plasmid. BODIPY specifically stains intracellular PHB (Politan et al. 2025) and was used here for qualitative single-cell comparison of PHB accumulation by flow cytometry. One of three technical replicates is shown (see Supplementary Fig. 4E for remaining replicates). **C** PHB titre [g/L] after 4 h in the PHB production medium for parental strain and ALE 5.1. **D** Extracellular PCA concentration and **E** biomass accumulation during the first four hours of PHB production. **F** Lag time (time to reach 0.5 × maximum OD_600_) and **G** growth rate in the seed culture prior to induction. For the experimental setup, see Supplementary Fig. 4A. Both seed and production cultures contained 0.1% PCA in optimised MOPS2 minimal medium. Data in **C**–**E** represent two biological replicates per strain, each measured in four technical replicates; data in **F** and **G** represent two biological replicates per strain, each measured in three technical replicates. The high variation in lag time (**F**) and growth rate (**G**) between replicates reflects the substantial physiological burden of maintaining kanamycin resistance and a PHB production plasmid while growing on 0.1% PCA; under these near-threshold conditions, small differences in inoculum density, initial OD, or the age of PCA-containing medium can lead to pronounced differences in growth kinetics. Statistical comparisons in panels **C**–**G**: unpaired two-tailed *t*-test (*p < 0.05, **p < 0.01, ***p < 0.001, ****p < 0.0001)

Finally, we evaluated whether *V. natriegens* can upcycle PCA into the biopolymer poly-3-hydroxybutyrate (PHB). *V. natriegens* was engineered to produce PHB from PCA using a plasmid-based expression system in which the native *phaBAPC* operon was placed under the control of an anhydrotetracycline (aTc)–inducible P_Tet_ promoter (Fig. 6A) (Politan et al. 2025). PHB accumulation per cell was monitored over a 12-h time course in the parental strain and four ALE lineages (ALE 4.1, 5.1, 7.1, and 8.1; Supplementary Fig. 4 A,E). In all strains, PHB content peaked after approximately 4 h and declined thereafter (Supplementary Fig. 4E; Fig. 6B). Among the tested strains, ALE 5.1 showed the fastest and highest PHB accumulation per cell (Supplementary Fig. 4D,E).

Based on this performance, ALE 5.1 was selected for a detailed comparison with the parental strain at the 4-h time point (Supplementary Fig. 4 F). Under these conditions, ALE 5.1 produced 0.131 g/L PHB, compared to 0.051 g/L in the parental strain (Fig. 6C), corresponding to 4.5% and 1.8% of cell dry weight, respectively. Notably, ALE 5.1 reached mid-exponential phase in the seed culture 1.24-fold faster than the parental strain, enabling higher PHB titres within a shorter cultivation time (Fig. 6F**; **Supplementary Fig. 4D). In contrast, PCA consumption (Fig. 6D; Supplementary Fig. 4 C), maximum biomass formation (Fig. 6E), and overall growth rate during PHB production (Fig. 6G) did not differ significantly between the two strains.

In summary, these experiments establish that *V. natriegens* can convert PCA into the non-aromatic biopolymer PHB. Adaptive laboratory evolution improved PHB titres primarily by shortening the lag phase before productive growth, rather than by enhancing substrate uptake, growth rate, or final biomass. Although PHB titres from PCA remain below those achieved with conventional substrates, these results demonstrate the feasibility of PCA-fed PHB production and highlight ALE as an effective strategy to improve the temporal performance of aromatic-based bioprocesses.

## Discussion

We demonstrate that *Vibrio natriegens* can metabolise protocatechuic acid (PCA) as a sole carbon source and convert it into the biodegradable polymer poly-3-hydroxybutyrate (PHB). By combining physiological characterisation, adaptive laboratory evolution (ALE), and metabolic engineering, this work establishes PCA as a viable aromatic feedstock for *V. natriegens* and identifies oxidative stress as the dominant constraint limiting growth and productivity. Adaptive evolution on PCA selected for convergent mutations in oxidative stress regulators and defence systems, enabling faster growth, improved robustness, and enhanced PHB production. Together, these findings expand the metabolic repertoire of *V. natriegens* and provide a conceptual framework for valorising aromatic substrates derived from lignin or plastic waste.

*V. natriegens* naturally catabolises PCA via the 4,5-meta cleavage pathway, consistent with earlier reports for this species and other marine bacteria (Hädrich et al. 2025b). Adaptive mutations optimise PCA catabolism efficiency. Two ALE lineages acquired adaptive mutations in a LysR-family regulator located upstream of the 4,5-meta cleavage pathway genes. LysR-type regulators are often divergently transcribed and frequently control nearby catabolic operons (Maddocks And Oyston 2008). The observed mutations may therefore have adjusted the regulator’s binding or activity to enhance expression of the 4,5-meta cleavage pathway genes. This would mean that in our evolved lineages, the *lysR* mutations likely help fine-tune the expression of PCA catabolic enzymes or transporters, further improving PCA consumption. However, LysR regulators are not always adjacent to their targets (Maddocks And Oyston 2008), and DNA-binding assays are required to confirm this in follow-up studies.

Our experimental data and genome-scale metabolic modelling confirm that channelling carbon exclusively through this pathway, while preventing flux from the shikimate pathway, supports efficient growth on PCA. Under standard MOPS2 minimal medium conditions, growth was sustained at up to 0.25% (w/v) PCA in shake flasks, and optimization of iron speciation (Fe^3^⁺ instead of Fe^2^⁺) enabled growth at 0.30% PCA. This places *V. natriegens* among other PCA-tolerant microorganisms described to date, comparable to *Azotobacter chroococcum* (Juarez et al. 2005) and exceeding many fungal and bacterial systems (Kuswandi And Roberts 1992; Spence et al. 2020; Tan et al. 2020) (Supplementary Table 16). However, industrially established aromatic-catabolising chassis such as *Pseudomonas putida* and *Corynebacterium glutamicum* exhibit higher PCA tolerance under optimised conditions (Kogure et al. 2021; Li And Ye 2021) (Supplementary Table 16). Nevertheless, the tolerance levels achieved here are notable for *V.* *natriegens*, which is not a canonical aromatic-catabolising chassis, and the ALE-mediated improvements represent a substantial gain relative to the parental strain.

We investigated five candidate transporters representing major aromatic uptake families, including MFS (Mori et al. 2018; Nichols and Harwood 1997; Pernstich et al. 2014), TTT (Hosaka et al. 2013), TRAP (Chae And Zylstra 2006), and TonB-dependent systems (Fujita et al. 2019; Noinaj et al. 2010). Surprisingly, deletion of individual transporters (or even all five simultaneously) did not impair intracellular PCA accumulation or growth in media with 0.1% PCA. This result could indicate (i) extensive redundancy in PCA uptake through as-yet unidentified transporters or (ii) sufficient passive diffusion at 0.1% PCA to sustain unimpaired growth. On the one hand, the hypothesis of transporter redundancy mirrors observations in other bacteria, where multiple aromatic acid transporters operate in parallel (Harwood et al. 1994; Salmon et al. 2013). Against this background, the enrichment of mutations in both TRAP transporters occurring in ALE lineage 8 is best interpreted as fine-tuning intracellular PCA concentrations rather reflecting essential uptake contributors. Alternatively, passive diffusion of the undissociated acid at neutral pH may dominate over transporter-mediated uptake at high PCA concentrations, driven by a steep PCA concentration gradient across the cytoplasmic membrane. In that case, PCA transporters would be less important for uptake at high aromatic acid concentrations and might even mediate efflux of toxic PCA. The mutations accumulated in ALE lineage 8 could then (i) reduce import activity or (ii) enhance efflux activity, thereby limiting intracellular PCA accumulation and alleviating toxicity. Together, the failure of transporter deletions to block uptake at high PCA concentrations, and the ALE-derived mutations suggest that further work is needed to determine whether PCA import in *V.* *natriegens* is mediated by redundant, currently unidentified transport systems or whether passive diffusion alone can sustain growth. Future studies searching for transporter homologues such as GalT from *Pseudomonas putida* (Nogales et al. 2011), growth experiments at lower PCA concentrations, and direct transport assays may resolve this question.

### Adaptive laboratory evolution reveals potential candidates for stress regulators in *V. natriegens*

Reduced growth at elevated PCA concentrations results primarily from cellular toxicity associated with PCA-mediated ROS production. PCA undergoes single-electron transfer reactions with oxygen, generating reactive oxygen species (ROS) that damage cellular components (Galano and Pérez-González 2012; Li et al. 2011). Increased PCA concentration, higher oxygenation (smaller culture volumes, faster shaking), and the presence of Fe^2^⁺ all caused growth inhibition, consistent with ROS formation and Fenton chemistry (Imlay et al. 1988). Bacteria counteract such stress by limiting intracellular Fe^2^⁺: RpoS-dependent induction of the iron storage protein Dps lowers dissolved cytoplasmic Fe^2+^ and manganese import through the OxyR/RpoS-regulated MntH allows Mn^2^⁺ to substitute for Fe^2^⁺ in enzymes (Bouillet et al. 2024). Our results show that these endogenous strategies can be reinforced through medium design. Replacing Fe^2^⁺ with Fe^3^⁺ reduced redox-active iron availability, improved PCA tolerance, and enhanced bioproduction performance. Thus, PCA can serve as a sole carbon source for *V. natriegens*—provided substrate concentration, oxygenation, and metal chemistry are carefully controlled.

**Fig. 7:**
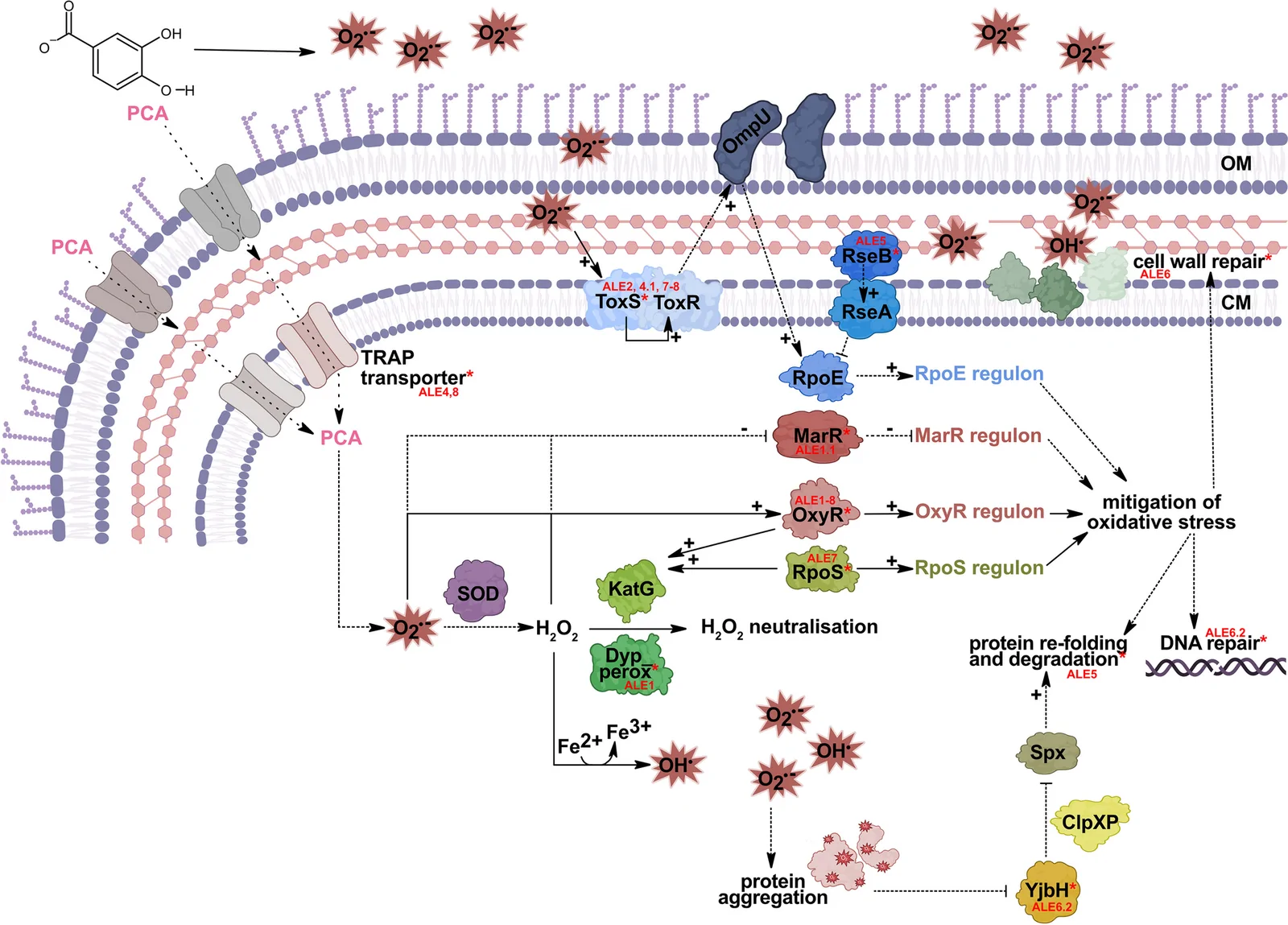
Components of the oxidative stress response in *V.* *natriegens* targeted during adaptive laboratory evolution on PCA. Adaptive mutations accumulated during ALE revealed proteins involved in the oxidative stress response, comprising both catalytic enzymes, such as the Dyp-type peroxidase, and regulatory proteins, including OxyR, MarR, RpoS, ToxS, and RseB. Adaptive mutations were also identified in proteins involved in repair of ROS-induced cell wall damage, detection and repair of damaged DNA, and refolding or degradation of damaged proteins. Proteins marked with a red asterisk were mutated in both colonies of an ALE lineage (unless indicated otherwise); text in red names the ALE lineages in which the mutations were found. Regulatory interactions are annotated by evidence level: solid arrows indicate connections experimentally validated in *V.* *natriegens* or closely related *Vibrio* species; dashed arrows indicate interactions inferred from well-characterised orthologues in *E. coli* or other proteobacteria; and dotted arrows indicate hypothetical connections based on gene synteny or domain architecture only. The TRAP transporter is shown as a candidate PCA uptake route based on co-localisation with the PCA catabolic operon and ALE mutation data; direct transport assays are required to confirm substrate specificity

Adaptive laboratory evolution on PCA revealed oxidative stress mitigation as the central adaptive target. ALE has previously improved PHB production (Politan et al. 2025), oxidative stress tolerance (Anand et al. 2020), and utilisation of challenging substrates in *V. natriegens* (Tian et al. 2023)—proving similarly effective in this study. The oxidative stress response network revealed by ALE is summarised in Fig. 7, where regulatory interactions (arrows) are annotated by evidence level (see figure legend).

*Vibrio* species are naturally exposed to oxidative stress in coastal environments (Morris et al. 2022; Thompson and Polz 2006). The presence of PCA generates additional ROS both intracellularly and extracellularly (Galano and Pérez-González 2012; Li et al. 2011). When ROS levels exceed detoxification capacity, they damage DNA, proteins, lipids, and membranes (Cumming et al. 2004). The redox-sensitive regulators OxyR and MarR are directly oxidised by ROS, rapidly activating their regulons (Alves et al. 2021; Cumming et al. 2004; Grove 2013). The universal occurrence of *oxyR* mutations across ALE lineages is therefore consistent with selection for faster or more constitutive activation of antioxidant defences. The truncation of MarR in ALE1.1 likely derepresses the MarR regulon, increasing expression of efflux pumps and other stress-protective genes. In this context, the efflux transporter gene *PN96_RS08945* mutated in ALE3.2 suggests that enhanced export of toxic compounds may be a recurring strategy for tolerance to aromatic stressors such as PCA (Su et al. 2025). In contrast to OxyR and MarR, RpoS is activated indirectly and primarily during stationary phase (Bouillet et al. 2024; Sazykin And Sazykina 2023). The persistence of a loss-of-function *rpoS* mutation in ALE 7—alongside enhanced OxyR activity in all lineages—implies that the RpoS-mediated stress response is less critical under PCA stress. Beyond this, there are additional mechanistic reasons why loss-of-function mutations in *rpoS* may be selectively advantageous in a continuous ALE regime favouring rapid growth. RpoS competes directly with the housekeeping sigma factor σ^70^ for core RNA polymerase binding. Reducing RpoS activity can therefore (i) decrease the metabolic burden of RpoS-regulated stress proteins not essential under PCA growth conditions; (ii) redirect RNA polymerase towards growth-promoting genes controlled by σ^70^; and (iii) derepress nutrient uptake and catabolic genes negatively regulated by RpoS. Analogous *rpoS* loss-of-function mutations have been frequently observed in *E. coli* ALE experiments on defined minimal media (Chen et al. 2004), providing evolutionary precedent for this trade-off. In summary, the ALE lineages seem to rely primarily on the fast, OxyR-driven ROS response, while the slower stationary-phase σ^S^ response appears less advantageous, allowing RpoS loss-of-function mutations to persist. Phenotypically, the adaptive mutations manifested as shortened lag phases and improve early survival under PCA stress, without changes in long-term viability under sustained oxidative challenge. Thus, adaptation favoured early-phase oxidative stress responses rather than stationary-phase tolerance.

At the enzymatic level, intracellular superoxide is converted to hydrogen peroxide by superoxide dismutase (Chiang and Schellhorn 2012; Gregory And Fridovich 1973; Scott et al. 1987), followed by detoxification via catalase–peroxidase KatG and a Dyp-type peroxidase (Glasgo 2024). Notably, *V. natriegens* is the only *Vibrio* species known to date encoding three *katG* paralogs (Glasgo 2024). However, previous work has reported poor native *katG* expression under standard laboratory conditions (Hädrich et al. 2025b; Weinstock et al. 2016), indicating that the mere presence of *katG* paralogs does not guarantee high peroxidase activity. Overexpression of KatG from a synthetic promoter, in combination with the PHB production plasmid, is therefore proposed as a priority for future rational engineering. Beyond ROS scavenging, Dyp-type peroxidases can degrade a wide range of aromatic compounds, including lignin-derived substrates (Gu et al. 2024; Salvachïa et al. 2013; Sugano and Yoshida 2021), though their direct role in PCA degradation remains unresolved (Ahmad et al. 2011). In *V.* *natriegens,* Dyp-type peroxidases are predicted to operate independently of OxyR regulation (Glasgo 2024). Thus, the mutated Dyp enzyme represents a parallel, OxyR-independent mechanism to bolster H_2_O_2_ degradation and relieve oxidative stress in the presence of PCA (Glasgo 2024; Sugano and Yoshida 2021).

Periplasmic ROS are sensed by the ToxRS system (Gubensäk et al. 2023), which activates outer-membrane porin OmpU (Mathur et al. 2007) and subsequently the extracytoplasmic function σ factor σ^E^ (RpoE) pathway through relief of RseA/RseB repression (De Las Peñas et al. 1997; Dou et al. 2018; Mathur et al. 2007; Mecsas et al. 1993; Whitaker et al. 2012). This cascade induces envelope stress responses that mitigate oxidative damage (Dou et al. 2018). The recurring *toxS*-related mutations in our ALE populations, as well as the mutation in *rseB,* suggest that *V.* *natriegens* likewise relies on this system to detect and respond to external oxidative stress (e.g. ROS generated extracellularly by PCA autoxidation). By fine-tuning this extracytoplasmic stress response, the ALE lineages likely improved their ability to withstand oxidative damage originating from outside the cytoplasm. Further work is needed to confirm the specific signals and regulon of the ToxRS system in *V.* *natriegens*.

Mutations affecting protein quality control complete the oxidative stress network revealed by ALE. These affect the redox-sensitive adaptor YjbH and its downstream regulator Spx (Engman et al. 2012; Engman And von Wachenfeldt 2015; Paudel et al. 2021), pointing to enhanced refolding or removal of oxidised proteins. Collectively, these adaptive changes provide a first map of a coordinated oxidative stress mitigation network in *V. natriegens* (summarised in Fig. 7) that had not previously been described for this organism.

### PHB production with parental strain and adaptive lineages

Building on this adapted physiology, we demonstrate PHB production from PCA as a sole carbon source. *V. natriegens* has previously been engineered to produce PHB from glucose (Dalia et al. 2017), glycerol (Li et al. 2023), and acetate (Politan et al. 2025), achieving up to 4%, 35%, and 46% of dry cell weight, respectively. Using a plasmid-based PHB pathway, we achieved 1.8% PHB per CDW (0.051 g/L) in the parental strain and 4.5% per CDW (0.131 g/L PHB) in the best PCA-adapted lineages, without supplementing additional carbon sources such as yeast extract or peptone. PHA production from aromatic acids has reached titres of up to 1.95 g/L, 1.04 g/L, and 0.95 g/L in *Halomonas hydrothermalis* LL1, *Ralstonia eutropha* H16, and *Pseudomonas putida* AG2162, respectively (Salvachúa et al. 2020; Tomizawa et al. 2014; Wang et al. 2025) (see Supplementary Table 1 for a comprehensive list). While modest in absolute terms, three insights emerge from our results. First, PHB accumulated rapidly, reaching maximum levels within 4 h, compared to ~ 24 h on acetate using the same plasmid-based production system (Politan et al. 2025). This indicates that substrates with higher carbon content such as PCA can support accelerated production. Second, ALE substantially improved PHB productivity, consistent with previous acetate-based studies (Politan et al. 2025). Third, lower PHB titres on PCA and glucose compared to acetate or glycerol (Dalia et al. 2017; Li et al. 2023; Politan et al. 2025) likely reflect suboptimal production conditions, in which significant carbon was diverted to biomass. Decoupling growth from production, improving PCA uptake, and optimising oxygenation to balance growth and ROS formation represent clear avenues for further improvement. We regard this study as a proof-of-concept; a full time-course optimisation is proposed for future process development work.

The use of PCA as a carbon source imposes distinct cofactor and redox constraints relevant to understanding and improving PHB yields. PCA is a highly oxidised substrate (degree of reduction per carbon ≈ 2.67, compared to ≈ 4.0 for glucose), limiting the generation of reduced cofactors (NADH, NADPH) per mole of carbon metabolised and constraining the maximum theoretical PHB yield. Additionally, the acetyl-CoA produced by 4,5-meta cleavage of PCA must be partitioned between PHB biosynthesis and the TCA cycle for biomass generation. A further complication is the elevated NADPH demand imposed by the oxidative stress response under PCA conditions—glutathione reductase and thioredoxin reductase consume NADPH to regenerate reduced antioxidant pools—which competes directly with the NADPH-requiring acetoacetyl-CoA reductase (PhaB) step in PHB biosynthesis. Future metabolic engineering strategies aimed at improving PHB yield from PCA should address these cofactor constraints explicitly, for example through (i) co-feeding an additional substrate as a source of reducing power or (ii) balancing flux between the TCA cycle and PHB biosynthesis, for instance via two-stage fermentation.

## Conclusion

This study establishes PCA as a viable sole carbon source for *V. natriegens* and provides the first proof-of-concept for PHB production from PCA in a *Vibrio* species. Although pure PCA is unlikely to serve as an industrial feedstock in the near-term (Jin et al. 2024; Lubbers And de Vries 2021; Zheng et al. 2024), its use here enabled systematic dissection of PCA toxicity, ROS defence, and oxidative stress adaptation in an emerging chassis organism. The convergent mutations uncovered by ALE identify key engineering targets for improving aromatic bioprocesses. More broadly, these findings deepen the understanding of redox biology in *V.* *natriegens* and support its continued development as a next-generation platform for sustainable bioproduction from aromatic waste streams.

## Supplementary Information

Below is the link to the electronic supplementary material.

ESM 1 (PDF 1.59 MB)

## Data Availability

Sequences of genetic parts, primer lists, plasmid maps, and assembly workflows are included in the Supplementary Information. All analyses and figure generation were performed using custom R scripts (R version 2021b). Datasets underlying the figures and all associated computational code can be obtained from the corresponding author upon request.

## References

[CR1] Abramson J, Adler J, Dunger J, Evans R, Green T, Pritzel A, Ronneberger O, Willmore L, Ballard AJ, Bambrick J, Bodenstein SW, Evans DA, Hung C-C, O’Neill M, Reiman D, Tunyasuvunakool K, Wu Z, Žemgulytė A, Arvaniti E, Beattie C, Bertolli O, Bridgland A, Cherepanov A, Congreve M, Cowen-Rivers AI, Cowie A, Figurnov M, Fuchs FB, Gladman H, Jain R, Khan YA, Low CMR, Perlin K, Potapenko A, Savy P, Singh S, Stecula A, Thillaisundaram A, Tong C, Yakneen S, Zhong ED, Zielinski M, Žídek A, Bapst V, Kohli P, Jaderberg M, Hassabis D, Jumper JM (2024) Accurate structure prediction of biomolecular interactions with AlphaFold 3. Nature 630:493–500. 10.1038/s41586-024-07487-w 38718835 10.1038/s41586-024-07487-w PMC11168924

[CR2] Ahmad M, Roberts JN, Hardiman EM, Singh R, Eltis LD, Bugg TDH (2011) Identification of DypB from *Rhodococcus jostii* RHA1 as a lignin peroxidase. Biochemistry 50:5096–5107. 10.1021/bi101892z 21534568 10.1021/bi101892z

[CR3] Ajiboye TO, Habibu RS, Saidu K, Haliru FZ, Ajiboye HO, Aliyu NO, Ibitoye OB, Uwazie JN, Muritala HF, Bello SA, Yusuf II, Mohammed AO (2017) Involvement of oxidative stress in protocatechuic acid-mediated bacterial lethality. Microbiol Open 6:e00472. 10.1002/mbo3.472 10.1002/mbo3.472 PMC5552917 28349673

[CR4] Al Shaer D, Al Musaimi O, de la Torre BG, Albericio F (2020) Hydroxamate siderophores: natural occurrence, chemical synthesis, iron binding affinity and use as Trojan horses against pathogens. Eur J Med Chem 208:112791. 10.1016/j.ejmech.2020.112791 32947228 10.1016/j.ejmech.2020.112791

[CR5] Alves JA, Previato-Mello M, Barroso KCM, Koide T, da Silva Neto JF (2021) The MarR family regulator OsbR controls oxidative stress response, anaerobic nitrate respiration, and biofilm formation in *Chromobacterium violaceum*. BMC Microbiol. 10.1186/s12866-021-02369-x 34736409 10.1186/s12866-021-02369-x PMC8567585

[CR6] Anand A, Chen K, Catoiu E, Sastry AV, Olson CA, Sandberg TE, Seif Y, Xu S, Szubin R, Yang L, Feist AM, Palsson BO (2020) OxyR is a convergent target for mutations acquired during adaptation to oxidative stress-prone metabolic states. Mol Biol Evol 37:660–667. 10.1093/molbev/msz251 31651953 10.1093/molbev/msz251 PMC7038661

[CR7] Bajwa DS, Pourhashem G, Ullah AH, Bajwa SG (2019) A concise review of current lignin production, applications, products and their environmental impact. Ind Crops Prod 139:111526. 10.1016/j.indcrop.2019.111526

[CR8] Battesti A, Majdalani N, Gottesman S (2011) The RpoS-mediated general stress response in *Escherichia coli*. Annu Rev Microbiol 65:189–213. 10.1146/annurev-micro-090110-102946 10.1146/annurev-micro-090110-102946 PMC7356644 21639793

[CR9] Bentley R, Haslam E (1990) The shikimate pathway — a metabolic tree with many branche. Crit Rev Biochem Mol Biol 25:307–384. 10.3109/10409239009090615 2279393 10.3109/10409239009090615

[CR10] Bouillet S, Bauer TS, Gottesman S (2024) RpoS and the bacterial general stress response. Microbiol Mol Biol Rev 88:e00151-22. 10.1128/mmbr.00151-22 38411096 10.1128/mmbr.00151-22 PMC10966952

[CR11] Chae J-C, Zylstra GJ (2006) 4-chlorobenzoate uptake in *Comamonas* sp. strain DJ-12 is mediated by a tripartite ATP-independent periplasmic transporter. J Bacteriol 188:8407–8412. 10.1128/JB.00880-06 17041053 10.1128/JB.00880-06 PMC1698221

[CR12] Chen G, Patten CL, Schellhorn HE (2004) Positive selection for loss of RpoS function in *Escherichia coli*. Mutat Res-Fundam Mol Mech Mutagen 554:193–203. 10.1016/j.mrfmmm.2004.04.013 10.1016/j.mrfmmm.2004.04.013 15450418

[CR13] Chiang SM, Schellhorn HE (2012) Regulators of oxidative stress response genes in *Escherichia coli* and their functional conservation in bacteria. Arch Biochem Biophys 525:161–169. 10.1016/j.abb.2012.02.007 10.1016/j.abb.2012.02.007 22381957

[CR14] Collier LS, Nichols NN, Neidle EL (1997) BenK encodes a hydrophobic permease-like protein involved in benzoate degradation by Acinetobacter sp. strain ADP1. J Bacteriol 179:5943–5946. 10.1128/jb.179.18.5943-5946.1997 9294456 10.1128/jb.179.18.5943-5946.1997 PMC179488

[CR15] Coppens L, Tschirhart T, Leary DH, Colston SM, Compton JR, Hervey WJ IV, Dana KL, Vora GJ, Bordel S, Ledesma-Amaro R (2023) *Vibrio natriegens* genome-scale modeling reveals insights into halophilic adaptations and resource allocation. Mol Syst Biol 19:e10523. 10.15252/msb.202110523 36847213 10.15252/msb.202110523 PMC10090949

[CR16] Crawford RL (1975) Novel pathway for degradation of protocatechuic acid in *Bacillus* species. J Bacteriol 121:531–536. 10.1128/jb.121.2.531-536.1975 163224 10.1128/jb.121.2.531-536.1975 PMC245963

[CR17] Cumming RC, andon NL, Haynes PA, Park M, Fischer WH, Schubert D (2004) Protein disulfide bond formation in the cytoplasm during oxidative stress. J Biol Chem 279:21749–21758. 10.1074/jbc.M312267200 15031298 10.1074/jbc.M312267200

[CR18] Dalia TN, Hayes CA, Stolyar S, Marx CJ, McKinlay JB, Dalia AB (2017) Multiplex genome editing by natural transformation (MuGENT) for synthetic biology in *Vibrio natriegens*. ACS Synth Biol 6:1650–1655. 10.1021/acssynbio.7b00116 28571309 10.1021/acssynbio.7b00116 PMC6519440

[CR19] Danso D, Chow J, Streit WR (2019) Plastics: environmental and biotechnological perspectives on microbial degradation. Appl Environ Microbiol. 10.1128/AEM.01095-19 31324632 10.1128/AEM.01095-19 PMC6752018

[CR20] De Las Peñas A, Connolly L, Gross CA (1997) The σ^E^-mediated response to extracytoplasmic stress in *Escherichia coli* is transduced by RseA and RseB, two negative regulators of σ^E^. Mol Microbiol 24:373–385. 10.1046/j.1365-2958.1997.3611718.x 9159523 10.1046/j.1365-2958.1997.3611718.x

[CR21] Della Valle S, Faber A, Politan RJ, Lama S, Fritz G (2025) From marsh to market: taming *Vibrio natriegens* for sustainable bioproduction. Curr Opin Biotechnol 96:103353. 10.1016/j.copbio.2025.103353 40945411 10.1016/j.copbio.2025.103353

[CR22] Diao J, Hu Y, Tian Y, Carr R, Moon TS (2023) Upcycling of poly(ethylene terephthalate) to produce high-value bio-products. Cell Rep 42:111908. 10.1016/j.celrep.2022.111908 36640302 10.1016/j.celrep.2022.111908

[CR23] Diao J, Tian Y, Hu Y, Moon TS (2024) Producing multiple chemicals through biological upcycling of waste poly(ethylene terephthalate). Trends Biotechnol. 10.1016/j.tibtech.2024.10.018 39581772 10.1016/j.tibtech.2024.10.018

[CR24] DiRita VJ, Mekalanos JJ (1991) Periplasmic interaction between two membrane regulatory proteins, ToxR and ToxS, results in signal transduction and transcriptional activation. Cell 64:29–37. 10.1016/0092-8674(91)90206-E 1898871 10.1016/0092-8674(91)90206-e

[CR25] Doench JG, Fusi N, Sullender M, Hegde M, Vaimberg EW, Donovan KF, Smith I, Tothova Z, Wilen C, Orchard R, Virgin HW, Listgarten J, Root DE (2016) Optimized sgRNA design to maximize activity and minimize off-target effects of CRISPR-Cas9. Nat Biotechnol 34:184–191. 10.1038/nbt.3437 26780180 10.1038/nbt.3437 PMC4744125

[CR26] Dou Y, Rutanhira H, Chen X, Mishra A, Wang C, Fletcher HM (2018) Role of extracytoplasmic function sigma factor PG1660 (RpoE) in the oxidative stress resistance regulatory network of *Porphyromonas gingivalis*. Mol Oral Microbiol 33:89–104. 10.1111/omi.12204 10.1111/omi.12204 PMC5823243 29059500

[CR27] Engman J, von Wachenfeldt C (2015) Regulated protein aggregation: a mechanism to control the activity of the ClpXP adaptor protein YjbH. Mol Microbiol 95:51–63. 10.1111/mmi.12842 25353645 10.1111/mmi.12842

[CR28] Engman J, Rogstam A, Frees D, Ingmer H, von Wachenfeldt C (2012) The YjbH adaptor protein enhances proteolysis of the transcriptional regulator Spx in *Staphylococcus aureus*. J Bacteriol 194:1186–1194. 10.1128/jb.06414-11 22194450 10.1128/JB.06414-11 PMC3294810

[CR29] Faber A, Politan RJ, Stukenberg D, Morris KM, Kim R, Jeon E, Inckemann R, Becker A, Thuronyi B, Fritz G (2025) Expanding genetic engineering capabilities in *Vibrio natriegens* with the Vnat Collection. Nucleic Acids Res 53:gkaf580. 10.1093/nar/gkaf580 40650977 10.1093/nar/gkaf580 PMC12255299

[CR30] Feng C-Q, Chen X-Q, Huang Q-S, Zhao X-M, Chen S, Xu K-W, Wu J, Yan Z-F (2025) Screening and engineering of lycopene-producing strain *Rhodococcus jostii* for bio-upcycling of poly(ethylene terephthalate) waste. Sci Total Environ 958:178168. 10.1016/j.scitotenv.2024.178168 39708736 10.1016/j.scitotenv.2024.178168

[CR31] Fuchs G, Boll M, Heider J (2011) Microbial degradation of aromatic compounds — from one strategy to four. Nat Rev Microbiol 9:803–816. 10.1038/nrmicro2652 21963803 10.1038/nrmicro2652

[CR32] Fujita M, Mori K, Hara H, Hishiyama S, Kamimura N, Masai E (2019) A tonB-dependent receptor constitutes the outer membrane transport system for a lignin-derived aromatic compound. Commun Biol. 10.1038/s42003-019-0676-z 31799434 10.1038/s42003-019-0676-z PMC6874591

[CR33] Galano A, Pérez-González A (2012) On the free radical scavenging mechanism of protocatechuic acid, regeneration of the catechol group in aqueous solution. Theor Chem Acc. 10.1007/s00214-012-1265-0

[CR34] Glasgo E (2024) Investigating oxidative stress and large-scale genomic reduction in *Vibrio natriegens* through the development and application of new genetic techniques (Doctorial Dissertation). University of Tennessee, Knoxville, Tennessee, Knoxville

[CR35] Grant CE, Bailey TL, Noble WS (2011) FIMO: scanning for occurrences of a given motif. Bioinformatics 27:1017–1018. 10.1093/bioinformatics/btr064 21330290 10.1093/bioinformatics/btr064 PMC3065696

[CR36] Gregory EM, Fridovich I (1973) Oxygen toxicity and the superoxide dismutase. J Bacteriol 114:1193–1197. 10.1128/jb.114.3.1193-1197.1973 4197269 10.1128/jb.114.3.1193-1197.1973 PMC285381

[CR37] Grevesse T, Guéguen C, Onana VE, Walsh DA (2022) Degradation pathways for organic matter of terrestrial origin are widespread and expressed in Arctic Ocean microbiomes. Microbiome. 10.1186/s40168-022-01417-6 36566218 10.1186/s40168-022-01417-6 PMC9789639

[CR38] Grove A (2013) MarR family transcription factors. Curr Biol 23:R142–R143. 10.1016/j.cub.2013.01.013 23428319 10.1016/j.cub.2013.01.013

[CR39] Gu J, Qiu Q, Yu Y, Sun X, Tian K, Chang M, Wang Y, Zhang F, Huo H (2024) Bacterial transformation of lignin: key enzymes and high-value products. Biotechnol Biofuels. 10.1186/s13068-023-02447-4 10.1186/s13068-023-02447-4 PMC10765951 38172947

[CR40] Gubensäk N, Sagmeister T, Buhlheller C, Geronimo BD, Wagner GE, Petrowitsch L, Gräwert MA, Rotzinger M, Berger TMI, Schäfer J, Usón I, Reidl J, Sánchez-Murcia PA, Zangger K, Pavkov-Keller T (2023) *Vibrio cholerae*’s ToxRS bile sensing system. eLife 12:e88721. 10.7554/eLife.88721 10.7554/eLife.88721 PMC10624426 37768326

[CR41] Hädrich M, Schulze C, Hoff J, Blombach B (2025b) *Vibrio natriegens*: Application of a fast-growing halophilic bacterium. Adv Biochem Eng Biotechnol 192:85–116. 10.1007/10_2024_271 10.1007/10_2024_271 39527262

[CR42] Hädrich M, Scheuchenegger C, Vital S-T, Gunkel C, Müller S, Hoff J, Borger J, Glawischnig E, Thoma F, Blombach B (2025a) Low-biomass pyruvate production with engineered *Vibrio natriegens* is accompanied by parapyruvate formation. Microb Cell Fact 24:73. 10.1186/s12934-025-02693-1 10.1186/s12934-025-02693-1 PMC11951559 40148976

[CR43] Han J, Zuo J, Zillante G, Chang R, Du L (2025) A systematic review of PET circularity technologies and management strategies: challenges and future directions. Resour Conserv Recycl 219:108280. 10.1016/j.resconrec.2025.108280

[CR44] Hara H, Masai E, Miyauchi K, Katayama Y, Fukuda M (2003) Characterization of the 4-carboxy-4-hydroxy-2-oxoadipate aldolase gene and operon structure of the protocatechuate 4,5-cleavage pathway genes in Sphingomonas paucimobilis SYK-6. J Bacteriol 185:41–50. 10.1128/jb.185.1.41-50.2003 12486039 10.1128/JB.185.1.41-50.2003 PMC141877

[CR45] Harwood CS, Parales RE (1996) The β-ketoadipate pathway and the biology of self-identity. Annu Rev Microbiol 50:553–590. 10.1146/annurev.micro.50.1.553 8905091 10.1146/annurev.micro.50.1.553

[CR46] Harwood CS, Nichols NN, Kim MK, Ditty JL, Parales RE (1994) Identification of the pcaRKF gene cluster from Pseudomonas putida: involvement in chemotaxis, biodegradation, and transport of 4-hydroxybenzoate. J Bacteriol 176:6479–6488. 10.1128/jb.176.21.6479-6488.1994 7961399 10.1128/jb.176.21.6479-6488.1994 PMC197001

[CR47] Hoff J, Daniel B, Stukenberg D, Thuronyi BW, Waldminghaus T, Fritz G (2020) *Vibrio natriegens*: an ultrafast-growing marine bacterium as emerging synthetic biology chassis. Environ Microbiol 22:4394–4408. 10.1111/1462-2920.15128 10.1111/1462-2920.15128 32537803

[CR48] Hosaka M, Kamimura N, Toribami S, Mori K, Kasai D, Fukuda M, Masai E (2013) Novel tripartite aromatic acid transporter essential for terephthalate uptake in *Comamonas* sp. strain E6. Appl Environ Microbiol 79:6148–6155. 10.1128/AEM.01600-13 10.1128/AEM.01600-13 PMC3811363 23913423

[CR49] Huccetogullari D, Luo ZW, Lee SY (2019) Metabolic engineering of microorganisms for production of aromatic compounds. Microb Cell Fact. 10.1186/s12934-019-1090-4 30808357 10.1186/s12934-019-1090-4 PMC6390333

[CR50] Imlay JA, Chin SM, Linn S (1988) Toxic DNA damage by hydrogen peroxide through the Fenton Reaction in vivo and in vitro. Science 240:640–642. 10.1126/science.2834821 2834821 10.1126/science.2834821

[CR51] Jiménez JI, Miñambres B, García JL, Díaz E (2002) Genomic analysis of the aromatic catabolic pathways from *Pseudomonas putida* KT2440. Environ Microbiol 4:824–841. 10.1046/j.1462-2920.2002.00370.x 10.1046/j.1462-2920.2002.00370.x 12534466

[CR52] Jin X, Li X, Zou Z, Zheng Z, Ouyang J (2024) Biological valorization of lignin-derived aromatics in hydrolysate to protocatechuic acid by engineered *Pseudomonas putida* KT2440. Molecules. 10.3390/molecules29071555 10.3390/molecules29071555 PMC11013400 38611834

[CR53] Jo G-A, Lee JM, No G, Kang DS, Kim S-H, Ahn S-H, Kong I-S (2015a) Isolation and characterization of a 17-kDa FKBP-type peptidyl-prolyl cis/trans isomerase from *Vibrio anguillarum*. Protein Expr Purif 110:130–137. 10.1016/j.pep.2015.02.019 10.1016/j.pep.2015.02.019 25747528

[CR54] Jo I, Chung I-Y, Bae H-W, Kim J-S, Song S, Cho Y-H, Ha N-C (2015b) Structural details of the OxyR peroxide-sensing mechanism. Proc Natl Acad Sci U S A 112:6443–6448. 10.1073/pnas.1424495112 25931525 10.1073/pnas.1424495112 PMC4443364

[CR55] Johnson CW, Salvachúa D, Khanna P, Smith H, Peterson DJ, Beckham GT (2016) Enhancing muconic acid production from glucose and lignin-derived aromatic compounds via increased protocatechuate decarboxylase activity. Metab Eng Commun 3:111–119. 10.1016/j.meteno.2016.04.002 29468118 10.1016/j.meteno.2016.04.002 PMC5779730

[CR56] Jovanović A, Bugarčić M, Simić M, Pejić J, Dimitrijević J (2024) The global market of PET production: from origins to recycling. Metall Mater Data 2:113–118. 10.30544/MMD46

[CR57] Juarez B, Martinez-Toledo MV, Gonzalez-Lopez J (2005) Growth of *Azotobacter chroococcum* in chemically defined media containing p-hydroxybenzoic acid and protocatechuic acid. Chemosphere 59:1361–1365. 10.1016/j.chemosphere.2004.11.037 15857648 10.1016/j.chemosphere.2004.11.037

[CR58] Kasai D, Fujinami T, Abe T, Mase K, Katayama Y, Fukuda M, Masai E (2009) Uncovering the protocatechuate 2,3-cleavage pathway genes. J Bacteriol 191:6758–6768. 10.1128/JB.00840-09 19717587 10.1128/JB.00840-09 PMC2795304

[CR59] Kim D, Kim M, Kim H-W, Kim E, Lee H (2025) Kraft lignin decomposition by lignin-derived aromatic compound degrader *Rhodococcus* sp. DK17. World J Microbiol Biotechnol. 10.1007/s11274-025-04350-6 40189716 10.1007/s11274-025-04350-6

[CR60] Kogure T, Suda M, Hiraga K, Inui M (2021) Protocatechuate overproduction by *Corynebacterium glutamicum* via simultaneous engineering of native and heterologous biosynthetic pathways. Metab Eng 65:232–242. 10.1016/j.ymben.2020.11.007 33238211 10.1016/j.ymben.2020.11.007

[CR61] Kuswandi K, Roberts CF (1992) Genetic control of the protocatechuic acid pathway in *Aspergillus nidulans*. Microbiology 138:817–823. 10.1099/00221287-138-4-817

[CR62] Lange R, Hengge-Aronis R (1991) Identification of a central regulator of stationary-phase gene expression in *Escherichia coli*. Mol Microbiol 5:49–59. 10.1111/j.1365-2958.1991.tb01825.x 1849609 10.1111/j.1365-2958.1991.tb01825.x

[CR63] Lévi-Meyrueis C, Monteil V, Sismeiro O, Dillies M-A, Kolb A, Monot M, Dupuy B, Duarte SS, Jagla B, Coppée J-Y, Beraud M, Norel F (2015) Repressor activity of the RpoS/σ^S^-dependent RNA polymerase requires DNA binding. Nucleic Acids Res 43:1456–1468. 10.1093/nar/gku1379 25578965 10.1093/nar/gku1379 PMC4330354

[CR64] Li X (2011) Preparation of RbCl super competent cells. Bio-Protoc 1:e76. 10.21769/BioProtoc.76

[CR65] Li J, Ye B-C (2021) Metabolic engineering of *Pseudomonas putida* KT2440 for high-yield production of protocatechuic acid. Bioresour Technol 319:124239. 10.1016/j.biortech.2020.124239 33254462 10.1016/j.biortech.2020.124239

[CR66] Li X, Wang X, Chen D, Chen S (2011) Antioxidant activity and mechanism of protocatechuic acid in vitro. Funct Foods Health Dis. 10.31989/ffhd.v1i7.127

[CR67] Li H-H, Wu J, Liu J-Q, Wu Q-Z, He RL, Cheng Z-H, Lv J-L, Lin W-Q, Wu J, Liu D-F, Li W-W (2023) Nonsterilized fermentation of crude glycerol for polyhydroxybutyrate production by metabolically engineered *Vibrio natriegens*. ACS Synth Biol 12:3454–3462. 10.1021/acssynbio.3c00498 37856147 10.1021/acssynbio.3c00498

[CR68] Lubbers RJM, de Vries RP (2021) Production of protocatechuic acid from p-hydroxyphenyl (H) units and related aromatic compounds using an *Aspergillus niger* cell factory. mBio. 10.1128/mbio.00391-21 34154420 10.1128/mBio.00391-21 PMC8262893

[CR69] Lushchak VI (2001) Oxidative stress and mechanisms of protection against it in bacteria. Biochem Mosc 66:476–489. 10.1023/A:1010294415625 10.1023/a:1010294415625 11405881

[CR70] Maddocks SE, Oyston PCF (2008) Structure and function of the LysR-type transcriptional regulator (LTTR) family proteins. Microbiology 154:3609–3623. 10.1099/mic.0.2008/022772-0 19047729 10.1099/mic.0.2008/022772-0

[CR71] Mathur J, Davis BM, Waldor MK (2007) Antimicrobial peptides activate the *Vibrio cholerae* σE regulon through an OmpU-dependent signalling pathway. Mol Microbiol 63:848–858. 10.1111/j.1365-2958.2006.05544.x 17181782 10.1111/j.1365-2958.2006.05544.x

[CR72] McDonald N, Achterberg EP, Carlson CA, Gledhill M, Liu S, Matheson-Barker JR, Nelson NB, Parsons RJ (2019) The role of heterotrophic bacteria and archaea in the transformation of lignin in the open ocean. Front Mar Sci. 10.3389/fmars.2019.00743

[CR73] Mecsas J, Rouviere PE, Erickson JW, Donohue TJ, Gross CA (1993) The activity of sigma E, an *Escherichia coli* heat-inducible sigma-factor, is modulated by expression of outer membrane proteins. Genes Dev 7:2618–2628. 10.1101/gad.7.12b.2618 8276244 10.1101/gad.7.12b.2618

[CR74] Mori K, Kamimura N, Masai E (2018) Identification of the protocatechuate transporter gene in *Sphingobium* sp. strain SYK-6 and effects of overexpression on production of a value-added metabolite. Appl Microbiol Biotechnol 102:4807–4816. 10.1007/s00253-018-8988-3 29675799 10.1007/s00253-018-8988-3

[CR75] Morris JJ, Rose AL, Lu Z (2022) Reactive oxygen species in the world ocean and their impacts on marine ecosystems. Redox Biol 52:102285. 10.1016/j.redox.2022.102285 35364435 10.1016/j.redox.2022.102285 PMC8972015

[CR76] Morya R, Kumar M, Kumar V, Thakur IS (2021) Biovalorization of lignin derived compounds with molasses as co-substrate for polyhydroxyalkanoate production. Environ Technol Innov 23:101695. 10.1016/j.eti.2021.101695

[CR77] Nakai S, Inoue Y, Hosomi M (2001) Algal growth inhibition effects and inducement modes by plant-producing phenols. Water Res 35:1855–1859. 10.1016/s0043-1354(00)00444-9 11329689 10.1016/s0043-1354(00)00444-9

[CR78] Neidhardt FC, Bloch PL, Smith DF (1974) Culture medium for enterobacteria. J Bacteriol 119:736–747. 10.1128/jb.119.3.736-747.1974 4604283 10.1128/jb.119.3.736-747.1974 PMC245675

[CR79] Ni B, Zhang Y, Chen D-W, Wang B-J, Liu S-J (2013) Assimilation of aromatic compounds by *Comamonas testosteroni*: characterization and spreadability of protocatechuate 4,5-cleavage pathway in bacteria. Appl Microbiol Biotechnol 97:6031–6041. 10.1007/s00253-012-4402-8 10.1007/s00253-012-4402-8 22996279

[CR80] Nichols NN, Harwood CS (1997) PcaK, a high-affinity permease for the aromatic compounds 4-hydroxybenzoate and protocatechuate from *Pseudomonas putida*. J Bacteriol 179:5056–5061. 10.1128/jb.179.16.5056-5061.1997 10.1128/jb.179.16.5056-5061.1997 PMC179362 9260946

[CR81] Nogales J, Canales Á, Jiménez-Barbero J, Serra B, Pingarrón JM, García JL, Díaz E (2011) Unravelling the gallic acid degradation pathway in bacteria: the gal cluster from *Pseudomonas putida*. Mol Microbiol 79:359–374. 10.1111/j.1365-2958.2010.07448.x 21219457 10.1111/j.1365-2958.2010.07448.x

[CR82] Noinaj N, Guillier M, Barnard TJ, Buchanan SK (2010) TonB-dependent transporters: regulation, structure, and function. Annu Rev Microbiol 64:43–60. 10.1146/annurev.micro.112408.134247 20420522 10.1146/annurev.micro.112408.134247 PMC3108441

[CR83] Opsahl S, Benner R (1997) Distribution and cycling of terrigenous dissolved organic matter in the ocean. Nature 386:480–482. 10.1038/386480a0

[CR84] Örn OE, Sacchetto S, van Niel EWJ, Hatti-Kaul R (2021) Enhanced protocatechuic acid production from glucose using *Pseudomonas putida* 3-dehydroshikimate dehydratase expressed in a phenylalanine-overproducing mutant of *Escherichia coli*. Front Bioeng Biotechnol. 10.3389/fbioe.2021.695704 34249890 10.3389/fbioe.2021.695704 PMC8264583

[CR85] Paudel A, Panthee S, Hamamoto H, Grunert T, Sekimizu K (2021) YjbH regulates virulence genes expression and oxidative stress resistance in *Staphylococcus aureus*. Virulence 12:470–480. 10.1080/21505594.2021.1875683 33487122 10.1080/21505594.2021.1875683 PMC7849776

[CR86] Payne WJ, Eagon RG, Williams AK (1961) Some observations on the physiology of *Pseudomonas natriegens* nov. spec. Antonie Van Leeuwenhoek 27:121–128. 10.1007/BF02538432 13733692 10.1007/BF02538432

[CR87] Pedre B, Young D, Charlier D, Mourenza Á, Rosado LA, Marcos-Pascual L, Wahni K, Martens E, G. de la Rubia A, Belousov VV, Mateos LM, Messens J (2018) Structural snapshots of OxyR reveal the peroxidatic mechanism of H_2_O_2_ sensing. Proc Natl Acad Sci USA 115:E11623–E11632. 10.1073/pnas.1807954115 30463959 10.1073/pnas.1807954115 PMC6294878

[CR88] Pernstich C, Senior L, MacInnes KA, Forsaith M, Curnow P (2014) Expression, purification and reconstitution of the 4-hydroxybenzoate transporter PcaK from *Acinetobacter* sp. ADP1. Protein Expr Purif 101:68–75. 10.1016/j.pep.2014.05.011 10.1016/j.pep.2014.05.011 PMC4148202 24907408

[CR89] Pfeifer E, Michniewski S, Gätgens C, Münch E, Müller F, Polen T, Millard A, Blombach B, Frunzke J (2019) Generation of a prophage-free variant of the fast-growing bacterium *Vibrio natriegens*. Appl Environ Microbiol 85:e00853–19. 10.1128/AEM.00853-19 10.1128/AEM.00853-19 PMC6696956 31253674

[CR90] Pfleger BF, Kim Y, Nusca TD, Maltseva N, Lee JY, Rath CM, Scaglione JB, Janes BK, anderson EC, Bergman NH, Hanna PC, Joachimiak A, Sherman DH (2008) Structural and functional analysis of AsbF: origin of the stealth 3,4-dihydroxybenzoic acid subunit for petrobactin biosynthesis. Proc Natl Acad Sci U S A 105:17133–17138. 10.1073/pnas.0808118105 18955706 10.1073/pnas.0808118105 PMC2579390

[CR91] Pham AN, Rose AL, Feitz AJ, Waite TD (2006) Kinetics of Fe(III) precipitation in aqueous solutions at pH 6.0–9.5 and 25 °C. Geochim Cosmochim Acta 70:640–650. 10.1016/j.gca.2005.10.018

[CR92] Pierrel F (2017) Impact of chemical analogs of 4-hydroxybenzoic acid on coenzyme Q biosynthesis: from inhibition to bypass of coenzyme Q deficiency. Front Physiol. 10.3389/fphys.2017.00436 28690551 10.3389/fphys.2017.00436 PMC5479927

[CR93] Politan RJ, Della Valle S, Pineda L, Joshi J, Euler C, Flematti G, Fritz G (2025) Establishing *Vibrio natriegens* as a high-performance host for acetate-based poly-3-hydroxybutyrate production. Metab Eng 92:22–38. 10.1016/j.ymben.2025.07.003 10.1016/j.ymben.2025.07.003 40653003

[CR94] Pryor JM, Potapov V, Kucera RB, Bilotti K, Cantor EJ, Lohman GJS (2020) Enabling one-pot Golden Gate assemblies of unprecedented complexity using data-optimized assembly design. PLoS One 15:e0238592. 10.1371/journal.pone.0238592 32877448 10.1371/journal.pone.0238592 PMC7467295

[CR95] Ramírez-Morales JE, Czichowski P, Besirlioglu V, Regestein L, Rabaey K, Blank LM, Rosenbaum MA (2021) Lignin aromatics to PHA polymers: nitrogen and oxygen are the key factors for *Pseudomonas*. ACS Sustain Chem Eng 9:10579–10590. 10.1021/acssuschemeng.1c02682

[CR96] Rosa LT, Bianconi ME, Thomas GH, Kelly DJ (2018) Tripartite ATP-independent periplasmic (TRAP) transporters and tripartite tricarboxylate transporters (TTT): from uptake to pathogenicity. Front Cell Infect Microbiol. 10.3389/fcimb.2018.00033 29479520 10.3389/fcimb.2018.00033 PMC5812351

[CR97] Rosini E, Battaglia C, Miani D, Molinari F, Arrigoni F, Piarulli U, Molla G, Pollegioni L (2025) Valuable compounds from pollutants: converting PET into enantiopure alanine. ACS Catal 15:17829–17843. 10.1021/acscatal.5c06530

[CR98] Sadler JC, Wallace S (2021) Microbial synthesis of vanillin from waste poly(ethylene terephthalate). Green Chem 23:4665–4672. 10.1039/D1GC00931A 34276250 10.1039/d1gc00931a PMC8256426

[CR99] Salmon RC, Cliff MJ, Rafferty JB, Kelly DJ (2013) The CouPSTU and TarPQM transporters in *Rhodopseudomonas palustris*: redundant, promiscuous uptake systems for lignin-derived aromatic substrates. PLoS One 8:e59844. 10.1371/journal.pone.0059844 23555803 10.1371/journal.pone.0059844 PMC3610893

[CR100] Salvachïa D, Prieto A, Martïnez ï, Martïnez MJ (2013) Characterization of a novel Dye-Decolorizing Peroxidase (DyP)-type enzyme from *Irpex lacteus* and its application in enzymatic hydrolysis of wheat straw. Appl Environ Microbiol 79:4316–4324. 10.1128/AEM.00699-13 23666335 10.1128/AEM.00699-13 PMC3697495

[CR101] Salvachúa D, Rydzak T, Auwae R, De Capite A, Black BA, Bouvier JT, Cleveland NS, Elmore JR, Furches A, Huenemann JD, Katahira R, Michener WE, Peterson DJ, Rohrer H, Vardon DR, Beckham GT, Guss AM (2020) Metabolic engineering of *Pseudomonas putida* for increased polyhydroxyalkanoate production from lignin. Microb Biotechnol 13:290–298. 10.1111/1751-7915.13481 31468725 10.1111/1751-7915.13481 PMC6922519

[CR102] Santos LDF, Lautru S, Pernodet J-L (2024) Genetic engineering approaches for the microbial production of vanillin. Biomolecules 14:1413. 10.3390/biom14111413 39595589 10.3390/biom14111413 PMC11591617

[CR103] Sasoh M, Masai E, Ishibashi S, Hara H, Kamimura N, Miyauchi K, Fukuda M (2006) Characterization of the terephthalate degradation genes of *Comamonas* sp. strain E6. Appl Environ Microbiol 72:1825–1832. 10.1128/AEM.72.3.1825-1832.2006 16517628 10.1128/AEM.72.3.1825-1832.2006 PMC1393238

[CR104] Sazykin IS, Sazykina MA (2023) The role of oxidative stress in genome destabilization and adaptive evolution of bacteria. Gene 857:147170. 10.1016/j.gene.2023.147170 36623672 10.1016/j.gene.2023.147170

[CR105] Scott MD, Meshnick SR, Eaton JW (1987) Superoxide dismutase-rich bacteria. Paradoxical increase in oxidant toxicity. J Biol Chem 262:3640–3645. 10.1016/S0021-9258(18)61401-2 3546314

[CR106] Seixas AF, Quendera AP, Sousa JP, Silva AFQ, Arraiano CM, andrade JM (2022) Bacterial response to oxidative stress and RNA oxidation. Front Genet. 10.3389/fgene.2021.821535 35082839 10.3389/fgene.2021.821535 PMC8784731

[CR107] Song G, Wu F, Peng Y, Jiang X, Wang Q (2022) High-level production of catechol from glucose by engineered *Escherichia coli*. Fermentation 8:344. 10.3390/fermentation8070344

[CR108] Sonoki T, Morooka M, Sakamoto K, Otsuka Y, Nakamura M, Jellison J, Goodell B (2014) Enhancement of protocatechuate decarboxylase activity for the effective production of muconate from lignin-related aromatic compounds. J Biotechnol 192:71–77. 10.1016/j.jbiotec.2014.10.027 25449108 10.1016/j.jbiotec.2014.10.027

[CR109] Spence EM, Scott HT, Dumond L, Calvo-Bado L, di Monaco S, Williamson JJ, Persinoti GF, Squina FM, Bugg TDH (2020) The hydroxyquinol degradation pathway in *Rhodococcus jostii* RHA1 and *Agrobacterium* species is an alternative pathway for degradation of protocatechuic acid and lignin fragments. Appl Environ Microbiol 86:e01561-20. 10.1128/AEM.01561-20 32737130 10.1128/AEM.01561-20 PMC7499046

[CR110] Storz G, Tartaglia LA, Ames BN (1990) The OxyR regulon. Antonie Van Leeuwenhoek 58:157–161. 10.1007/BF00548927 2256675 10.1007/BF00548927

[CR111] Stukenberg D, Hensel T, Hoff J, Daniel B, Inckemann R, Tedeschi JN, Nousch F, Fritz G (2021) The Marburg Collection: a Golden Gate DNA assembly framework for synthetic biology applications in *Vibrio natriegens*. ACS Synth Biol 10:1904–1919. 10.1021/ACSSYNBIO.1C00126/SUPPL_FILE/SB1C00126_SI_001.PDF 34255476 10.1021/acssynbio.1c00126

[CR112] Stukenberg D, Hoff J, Faber A, Becker A (2022) NT-CRISPR, combining natural transformation and CRISPR-Cas9 counterselection for markerless and scarless genome editing in *Vibrio natriegens*. Commun Biol 5:1–13. 10.1038/s42003-022-03150-0 35338236 10.1038/s42003-022-03150-0 PMC8956659

[CR113] Stumm W, Lee GF (1961) Oxygenation of ferrous iron. Ind Eng Chem 53:143–146. 10.1021/ie50614a030

[CR114] Su C, Cui H, Wang W, Liu Y, Cheng Z, Wang C, Yang M, Qu L, Li Y, Cai Y, He S, Zheng J, Zhao P, Xu P, Dai J, Tang H (2025) Bioremediation of complex organic pollutants by engineered *Vibrio natriegens*. Nature. 10.1038/s41586-025-08947-7 40335686 10.1038/s41586-025-08947-7

[CR115] Sugano Y, Yoshida T (2021) DyP-type peroxidases: recent advances and perspectives. Int J Mol Sci 22:5556. 10.3390/ijms22115556 34074047 10.3390/ijms22115556 PMC8197335

[CR116] Tan J, Li Y, Hou D-X, Wu S (2019) The effects and mechanisms of cyanidin-3-glucoside and its phenolic metabolites in maintaining intestinal integrity. Antioxidants (Basel) 8:479. 10.3390/antiox8100479 31614770 10.3390/antiox8100479 PMC6826635

[CR117] Tan X, Zhu J, Wakisaka M (2020) Effect of protocatechuic acid on *Euglena gracilis* growth and accumulation of metabolites. Sustainability 12:9158. 10.3390/su12219158

[CR118] Tang H, Wang M-J, Gan X-F, Li Y-Q (2022) Funneling lignin-derived compounds into polyhydroxyalkanoate by *Halomonas* sp. Y3. Bioresour Technol 362:127837. 10.1016/j.biortech.2022.127837 36031122 10.1016/j.biortech.2022.127837

[CR119] Thompson JR, Polz MF (2006) Dynamics of *Vibrio* populations and their role in environmental nutrient cycling. The biology of Vibrios. John Wiley & Sons, Ltd, pp 190–203. 10.1128/9781555815714.ch13

[CR120] Tian J, Deng W, Zhang Z, Xu J, Yang G, Zhao G, Yang S, Jiang W, Gu Y (2023) Discovery and remodeling of *Vibrio natriegens* as a microbial platform for efficient formic acid biorefinery. Nat Commun. 10.1038/s41467-023-43631-2 38012202 10.1038/s41467-023-43631-2 PMC10682008

[CR121] Toledano MB, Kullik I, Trinh F, Baird PT, Schneider TD, Storz G (1994) Redox-dependent shift of OxyR-DNA contacts along an extended DNA-binding site: a mechanism for differential promoter selection. Cell 78:897–909. 10.1016/S0092-8674(94)90702-1 8087856 10.1016/s0092-8674(94)90702-1

[CR122] Tomizawa S, Chuah J-A, Matsumoto K, Doi Y, Numata K (2014) Understanding the limitations in the biosynthesis of polyhydroxyalkanoate (PHA) from lignin derivatives. ACS Sustain Chem Eng 2:1106–1113. 10.1021/sc500066f

[CR123] Wang Y, Hu Y-S, Tang H, Li Y-Q, Luo C-B (2025) Highly efficient conversion of lignin-derived aromatic compounds into polyhydroxyalkanoate using *Halomonas hydrothermalis* LL1. Ind Crop Prod 236:122122. 10.1016/j.indcrop.2025.122122

[CR124] Weaver LM, Herrmann KM (1997) Dynamics of the shikimate pathway in plants. Trends Plant Sci 2:346–351. 10.1016/S1360-1385(97)84622-5

[CR125] Wei Q, Le Minh PN, Dötsch A, Hildebrand F, Panmanee W, Elfarash A, Schulz S, Plaisance S, Charlier D, Hassett D, Häussler S, Cornelis P (2012) Global regulation of gene expression by OxyR in an important human opportunistic pathogen. Nucleic Acids Res 40:4320–4333. 10.1093/nar/gks017 22275523 10.1093/nar/gks017 PMC3378865

[CR126] Weinstock MT, Hesek ED, Wilson CM, Gibson DG (2016) *Vibrio natriegens* as a fast-growing host for molecular biology. Nat Methods 13:849–851. 10.1038/nmeth.3970 27571549 10.1038/nmeth.3970

[CR127] Werner AZ, Clare R, Mand TD, Pardo I, Ramirez KJ, Haugen SJ, Bratti F, Dexter GN, Elmore JR, Huenemann JD, Peabody GL, Johnson CW, Rorrer NA, Salvachúa D, Guss AM, Beckham GT (2021) Tandem chemical deconstruction and biological upcycling of poly(ethylene terephthalate) to β-ketoadipic acid by *Pseudomonas putida* KT2440. Metab Eng 67:250–261. 10.1016/j.ymben.2021.07.005 34265401 10.1016/j.ymben.2021.07.005

[CR128] Whitaker WB, Parent MA, Boyd A, Richards GP, Boyd EF (2012) The *Vibrio parahaemolyticus* ToxRS regulator is required for stress tolerance and colonization in a novel orogastric streptomycin-induced adult murine model. Infect Immun 80:1834–1845. 10.1128/iai.06284-11 22392925 10.1128/IAI.06284-11 PMC3347455

[CR129] Wilkes RA, Waldbauer J, Caroll A, Nieto-Domínguez M, Parker DJ, Zhang L, Guss AM, Aristilde L (2023) Complex regulation in a *Comamonas* platform for diverse aromatic carbon metabolism. Nat Chem Biol. 10.1038/s41589-022-01237-7 36747056 10.1038/s41589-022-01237-7 PMC10154247

[CR130] Wu Y, Xiang W, Fu X, Yan S, Su J, Liu J, Bao Z (2016) Geochemical interactions between iron and phenolics originated from peatland in Hani, China: implications for effective transport of iron from terrestrial systems to marine. Environ Earth Sci. 10.1007/s12665-015-5189-6

[CR131] Yoshida S, Hiraga K, Takehana T, Taniguchi I, Yamaji H, Maeda Y, Toyohara K, Miyamoto K, Kimura Y, Oda K (2016) A bacterium that degrades and assimilates poly(ethylene terephthalate). Science 351:1196–1199. 10.1126/science.aad6359 26965627 10.1126/science.aad6359

[CR132] Zangrando R, Corami F, Barbaro E, Grosso A, Barbante C, Turetta C, Capodaglio G, Gambaro A (2019) Free phenolic compounds in waters of the Ross Sea. Sci Total Environ 650:2117–2128. 10.1016/j.scitotenv.2018.09.360 30290353 10.1016/j.scitotenv.2018.09.360

[CR133] Zhang H, Pereira B, Li Z, Stephanopoulos G (2015) Engineering *Escherichia coli* coculture systems for the production of biochemical products. Proc Natl Acad Sci USA 112:8266–8271. 10.1073/pnas.1506781112 26111796 10.1073/pnas.1506781112 PMC4500268

[CR134] Zheng S, Xu X, Gao T, Song H (2024) One-pot microbial cell factory strategy for the production of protocatechuic acid from polyethylene terephthalate waste. ACS Sustain Chem Eng 12:5632–5639. 10.1021/acssuschemeng.4c00363

