# Supplementary material for: Mitigating protocatechuic acid toxicity in Vibrio natriegens enables poly-3-hydroxybutyrate production from an aromatic carbon source
Source: Appl Microbiol Biotechnol. 2026 Apr 27;110(1):179. doi: 10.1007/s00253-026-13831-z (PMC13253662; doi:10.1007/s00253-026-13831-z)
Supplement: Supplementary file 1 — (PDF 1.59 MB) [file 253_2026_13831_MOESM1_ESM.pdf]

Supplementary Material to

Mitigating protocatechuic acid toxicity in *Vibrio natriegens* enables poly-3-hydroxybutyrate production from an aromatic carbon source

Anna Faber†[1,2,3], Roland Politan,[1] Angus Nicol[1], Simona Della Valle[1], Benjamin Jenkins[1], Gavin Flematti[1], and Georg Fritz*[1,2]

1 School of Molecular Sciences, The University of Western Australia, Perth, Australia

2 Oceans Institute, The University of Western Australia, Perth, Australia

3 Forrest Research Foundation, Perth, Australia

*For correspondence: georg.fritz@uwa.edu.au

Supplementary information on PHA production from aromatic or lignin-derived carbon sources in representative organisms

Supplementary Table 1. Reported PHA titres from aromatic or lignin-derived carbon sources in representative organisms. CDW – cell dry weight.

**Species**	**Substrate**	**PHA type**	**PHA titre [g/L]**	**Content [% CDW]**	**Reference**
Burkholderia sp. ISTR5 (R5)	Kraft lignin	polyhydroxy-butyrate-co-hydroxy-valerate (PHBV)	0.098	23	(Morya et al., 2021b)
Burkholderia sp. ISTR5	4-hydroxy-benzoic acid	polyhydroxy-butyrate-co-hydroxy-valerate (PHBV)	0.260	n.r.	(Morya et al., 2021a)
Burkholderia sp. ISTR5	*p*-couramic acid	polyhydroxy-butyrate-co-hydroxy-valerate (PHBV)	0.270	83	(Morya et al., 2021a)
Burkholderia sp. ISTR5	gallic acid	polyhydroxy-butyrate-co-hydroxy-valerate (PHBV)	0.256	82	(Morya et al., 2021a)
Cupriavidus basilensis B-8	Kraft lignin	polyhydroxyalkanoate (PHA)	0.319	n.r.	(Shi et al., 2017)
Halomonas sp. Y3	4-hydroxy-benzoic acid	polyhydroxyalkanoate (PHA)	0.535	n.r.	(Tang et al., 2022)
Halomonas sp. Y3	protocatechuic acid (PCA)	polyhydroxyalkanoate (PHA)	0.507	n.r.	(Tang et al., 2022)
Halomonas sp. Y3	catechol	polyhydroxyalkanoate (PHA)	0.436	n.r.	(Tang et al., 2022)
Halomonas sp. Y3	vanillic acid	polyhydroxyalkanoate (PHA)	0.441	n.r.	(Tang et al., 2022)
Halomonas hydrothermalis LL1	vanillic acid	polyhydroxyalkanoate (PHA)	1.950	64	(Wang et al., 2025)
Pandoraea sp. ISTKB	4-hydroxy-benzoic acid	polyhydroxyalkanoate (PHA)	0.246	47	(Kumar et al., 2017)
Pandoraea sp. ISTKB	*p*-coumaric acid	polyhydroxyalkanoate (PHA)	0.170	41	(Kumar et al., 2017)
Pandoraea sp. ISTKB	vanillic acid	polyhydroxyalkanoate (PHA)	0.072	33	(Kumar et al., 2017)
Pandoraea sp. ISTKB	2,6-di-methoxy-phenol	polyhydroxyalkanoate (PHA)	0.069	35	(Kumar et al., 2017)

Pandoraea sp. ISTKB	Kraft lignin	polyhydroxyalkanoate (PHA)	0.018	21	(Kumar et al., 2017)
Pseudomonas putida strain $A_{xyl\_alkKphaGC1}$	vanillic acid	polyhydroxyalkanoate (PHA)	0.246	34	(Wang et al., 2018)
Pseudomonas putida AG2162	p-coumaric acid	mcl-polyhydroxyalkanoate (mcl-PHA)	0. 953	54	(Salvachúa et al., 2020)
Pseudomonas putida KT2440	defined aromatic mixture of *p*-coumarate, ferulate, and benzoate	mcl-polyhydroxyalkanoate (mcl-PHA)	0. 582	43	(Ramírez-Morales et al., 2021)
Ralstonia eutropha H16	3-hydroxy-benzoic acid	poly-3-hydroxybutyrate (P3HB or PHB)	1.040	65	(Tomizawa et al., 2014)
Ralstonia eutropha H16	4-hydroxy-benzoic acid	poly-3-hydroxybutyrate (P3HB or PHB)	0.435	63	(Tomizawa et al., 2014)
Vibrio natriegens	PCA	poly-3-hydroxybutyrate (P3HB or PHB)	0.051	4.5	This study

Supplementary information on strains, oligonucleotides, media and methods used in this study

Supplementary Table 2. *V. natriegens* strains and lineages used in this study. KO – knockout.

	Internal strain name	Plasmid	Genotype	Remarks	Reference or source
**parental strain**	AF279-AF281 (=GFN22)	none	*V. natriegens* DSM 759 Δ*dns* ΔVNP1+2	Parental strain; Biological replicate 1-3 used for growth characterization on PCA	strain derived from DSM 759; (Politan et al., 2025)
	AF614-615 (=GFN22)	none	*V. natriegens* DSM 759 Δ*dns* ΔVNP1+2	Parental strain; Biological replicates 1-2 used for ALE on PCA	
**KO**	GFN320-GFN321	none	*V. natriegens* DSM 759 Δ*dns* ΔVNP1+2 Δ*mfs* Δ*tct* Δ*trap1* Δ*trap2* Δ*abc*	Biological replicate 1-2 of *V. natriegens* with all five transporter knockouts	This study
**PCA biosensor strains**	AF1082-AF1084	pAF1_72_lux-operon_pPcaU	*V. natriegens* DSM 759 Δ*dns* ΔVNP1+2 (AF614)	Biological replicate 1-3 of parental strain with PCA-biosensor plasmid	This study
	AF1088-AF1090	pAF1_72_lux-operon_pPcaU	*V. natriegens* DSM 759 Δ*dns* ΔVNP1+2 Δ*mfs*	Biological replicate 1-3 of MFS knockout with PCA-biosensor plasmid	This study
	AF1282-AF1284	pAF1_72_lux-operon_pPcaU	*V. natriegens* DSM 759 Δ*dns* ΔVNP1+2 Δ*ttt*	Biological replicate 1-3 of TTT knockout with PCA-biosensor plasmid	This study
	AF1118-AF1120	pAF1_72_lux-operon_pPcaU	*V. natriegens* DSM 759 Δ*dns* ΔVNP1+2 Δ*trap1*	Biological replicate 1-3 of TRAP1 knockout with PCA-biosensor plasmid	This study
	AF1122-AF1124	pAF1_72_lux-operon_pPcaU	*V. natriegens* DSM 759 Δ*dns* ΔVNP1+2 Δ*trap*2	Biological replicate 1-3 of TRAP2 knockout with PCA-biosensor plasmid	This study

	AF1126-AF1130	pAF1_72_lux-operon_pPcaU	*V. natriegens* DSM 759 Δ*dns* ΔVNP1+2 Δ*abc*	Biological replicate 1-3 of ABC transporter knockout with PCA-biosensor plasmid	This study
	AF1134-AF1138	pAF1_72_lux-operon_pPcaU	*V. natriegens* DSM 759 Δ*dns* ΔVNP1+2 Δ*trap1* Δ*trap2*	Biological replicate 1-3 of TRAP1-TRAP2 knockout with PCA-biosensor plasmid	This study
	AF1142-AF1146	pAF1_72_lux-operon_pPcaU	*V. natriegens* DSM 759 Δ*dns* ΔVNP1+2 Δ*trap1* Δ*trap2* Δ*abc*	Biological replicate 1-3 of TRAP1-TRAP2-ABC transporter knockout with PCA-biosensor plasmid	This study
	AF1150-AF1154	pAF1_72_lux-operon_pPcaU	*V. natriegens* DSM 759 Δ*dns* ΔVNP1+2 Δ*mfs* Δ*tct* Δ*trap1* Δ*trap2* Δ*abc*	Biological replicate 1-3 of all five transporter knockouts with PCA-biosensor plasmid	This study
**ALE lineages**	AF616-AF617	None	Adaptively evolved *V. natriegens* DSM 759 Δ*dns* ΔVNP1+2 lineage 1	Biological replicate 1-2 of adapted *V. natriegens* lineage 1	This study
	AF820-AF821	None	Adaptively evolved *V. natriegens* DSM 759 Δ*dns* ΔVNP1+2 lineage 2	Biological replicate 1-2 of adapted *V. natriegens* lineage 2	This study
	AF822-AF823	None	Adaptively evolved *V. natriegens* DSM 759 Δ*dns* ΔVNP1+2 lineage 3	Biological replicate 1-2 of adapted *V. natriegens* lineage 3	This study
	AF824-AF825	None	Adaptively evolved *V. natriegens* DSM 759 Δ*dns* ΔVNP1+2 cell line 4	Biological replicate 1-2 of adapted *V. natriegens* lineage 4	This study
	AF624-AF625	None	Adaptively evolved *V. natriegens* DSM 759 Δ*dns*	Biological replicate 1-2 of adapted *V. natriegens* lineage 5	This study

			ΔVNP1+2 lineage 5		
	AF0826-AF0827	None	Adaptively evolved *V. natriegens* DSM 759 Δ*dns* ΔVNP1+2 lineage 6	Biological replicate 1-2 of adapted *V. natriegens* lineage 6	This study
	AF828-AF829	None	Adaptively evolved *V. natriegens* DSM 759 Δ*dns* ΔVNP1+2 lineage 7	Biological replicate 1-2 of adapted *V. natriegens* lineage 7	This study
	AF0830-AF0831	None	Adaptively evolved *V. natriegens* DSM 759 Δ*dns* ΔVNP1+2 lineage 8	Biological replicate 1-2 of adapted *V. natriegens* lineage 8	This study
**PHB production strains**	AF908-AF909	P_{Tet}-*phaBAPC*	*V. natriegens* DSM 759 Δ*dns* ΔVNP1+2 (AF614)	Biological replicate 1-2 of parental *V. natriegens* with plasmid-based PHB system	Plasmid originally from (Politan et al., 2025), strain recreated in this study
	AF0910-AF911	P_{Tet}-*phaBAPC*	Adaptively evolved *V. natriegens* DSM 759 Δ*dns* ΔVNP1+2 lineage 1 (AF616)	Biological replicate 1-2 of adapted *V. natriegens* lineage 1 with plasmid-based PHB system	This study
	AF954-AF955	P_{Tet}-*phaBAPC*	Adaptively evolved *V. natriegens* DSM 759 Δ*dns* ΔVNP1+2 lineage 4 (AF824)	Biological replicate 1-2 of adapted *V. natriegens* lineage 4 with plasmid-based PHB system	This study
	AF912-AF913	P_{Tet}-*phaBAPC*	Adaptively evolved *V. natriegens* DSM 759 Δ*dns*	Biological replicate 1-2 of adapted *V. natriegens* lineage 5 with plasmid-based PHB system	This study

			ΔVNP1+2 lineage 5 (AF624)		
	AF914-AF915	P_{Tet}-*phaBAPC*	Adaptively evolved *V. natriegens* DSM 759 Δ*dns* ΔVNP1+2 lineage 7 (AF828)	Biological replicate 1-2 of adapted *V. natriegens* lineage 7 with plasmid-based PHB system	This study
	AF916-AF917	P_{Tet}-*phaBAPC*	Adaptively evolved *V. natriegens* DSM 759 Δ*dns* ΔVNP1+2 lineage 8 (AF830)	Biological replicate 1-2 of adapted *V. natriegens* lineage 8 with plasmid-based PHB system	This study

Supplementary Table 3. Precultures and culture conditions for all experiments. CGQ – Cell growth quantification with CGQ (Scientific Bio). kan – kanamycin, MM – minimal medium, NA – not applicable, * - optimized MOPS2 minimal medium.

inoculate inoculate inoculate

**Experi-ment**	**Figure**	**Strains**	**Over-night culture (OC)**	**Day culture 1 (DC1)**	**Day culture 2 (DC2)**	**Main experiment**
PCA uptake rates in shake flasks (with data from Supplementary Table **13**)	**Supplementary Figure 1B-D, Supplementary Figure 2C**	parental *V. natriegens* (AF614-615) and strain with 5 transporter deletions (GFN3210-GFN321)	LBv2, 30 °C, 220 rpm, 5 mL in glass tube	0.1 % PCA + 0.1 % Glc MOPS2 MM, 37 °C, 220 rpm, 2 mL in glass tube, 2 h, inoculated from OC dilution 1:100	0.1 % PCA MOPS2 MM, 37 °C, 220 rpm, 5 mL in glass tube, 3 h, inoculated from DC1 dilution 1:10	0.1 % PCA MOPS2 MM, 37 °C, 220 rpm, 20 mL in non-baffled 250-mL-flask, 6 h, inoculated from DC2 with starting OD_{600} of 0.05
growth rates in 96-well plates	Error! Reference source not found.**A-B**	parental *V. natriegens* (AF279-AF281)	0.1 % PCA + 0.1 % Glc MOPS2 MM, 30 °C, 220 rpm,	0.1 % PCA + 0.1 % Glc MOPS2 MM, 37 °C, 220 rpm, 2 mL in glass tube,	NA	different concentration of PCA in MOPS2 MM, 37 °C, orbital shaking, 100 µL in 96-well-

			2 mL in glass tube	5 h, inoculated from OC 1:200		plate, 24 h, inoculated from DC1 1:20
growth in shake flasks	Error! Reference source not found.**C**	parental *V. natriegens* (AF279-AF281)	0.1 % Glc MOPS2 MM, 30 °C, 220 rpm, 5 mL in glass tube	0.1 % Glc MOPS2 MM, 37 °C, 220 rpm, 5 mL in glass tube, inoculated from OC 1:100	0.1 % PCA + 0.1 % Glc MOPS2 MM, 37 °C, 220 rpm, 2 mL in glass tube, 2 h, inoculated from OC dilution 1:10	0.1-0.3 % PCA MOPS2 MM, 37 °C, 220 rpm, 20 mL in non-baffled 250-mL-flask, 6 h, inoculated from DC2 with starting OD_{600} of 0.05 (=inoculated from DC1 1:20 – 1:21)
intra-cellular PCA concentrations in 96-well plates	**Error! Reference source not found.C,** **Supplementary Figure 2A-B**	parental *V. natriegens* (AF614) and *V. natriegens* strains with PCA biosensor plasmids (AF1082-AF1084, AF1088-AF1090, AF1282, AF1284, AF1118, AF1120, AF1122, AF1124, AF1126, AF1130, AF1134, AF1138, AF1142, AF1146, AF1150, AF1154)	LBv2 + kan, 30 °C, 300 rpm, 1 mL in 2.2-mL-deep-well-plate	0.2 % Glc MOPS2 MM, 37 °C, 300 rpm, 1 mL in 2.2-mL-deep-well-plate, 5 h, inoculated from OC 1:200	NA	0.1 % PCA and/or 0.1 % Glc in MOPS2 MM, 37 °C, orbital shaking, 100 µL in 96-well-plate, 24 h, inoculated from DC1 1:100 when Glc present and 1:40 when only PCA as carbon source
ALE	**Error! Reference source not found.**, **Supplementary Figure 4A**	eight independent cell lines of *V. natriegens* parental strain (AF614-AF615)	0.1 % Glc MOPS2 MM, 30 °C, 300 rpm, 1 mL in 2.2-mL deep-well plate	0.2 % PCA + 0.1 % Glc MOPS2 MM, 37 °C, 300 rpm, 1 mL in 2.2-mL deep-well	NA	0.2 % PCA MOPS2 MM, 37 °C, 300 rpm, 0.4 mL in 2.2-mL deep-well plate, from

				plate, from OC dilution 1:100		DC1dilution 1:10 → continuous cultivation over 60 days
CGQ on different PCA concentra tions	Error! Reference source not found.**B**	*V. natriegens* parental strain (AF614) and ALE lineages (AF616, AF824, AF624, AF828, AF830)	LBv2, 30 °C, 220 rpm, 5 mL in glass tube	0.1 % PCA + 0.1 % Glc MOPS2 MM*, 37 °C, 220 rpm, 2 mL in glass tube, 2 h, inoculated from OC dilution 1:100	0.1 % PCA MOPS2 MM*, 37 °C, 220 rpm, 5 mL in glass tube, 3 h, inoculated from DC1 dilution 1:10	0.1-0.3 % PCA MOPS2 MM*, 37 °C, 220 rpm, 20 mL in non-baffled 250-mL-flask, 6 h, inoculated from DC2 with starting OD_{600} of 0.05
CGQ at different O_2-levels	Error! Reference source not found.**E**	*V. natriegens* parental strain (AF614) and ALE lineage 5 (AF624)	LBv2, 30 °C, 220 rpm, 5 mL in glass tube	0.1 % PCA + 0.1 % Glc MOPS2 MM*, 37 °C, 220 rpm, 2 mL in glass tube, 2 h, inoculated from OC dilution 1:100	0.1 % PCA MOPS2 MM*, 37 °C, 220 rpm, 5 mL in glass tube, 3 h, inoculated from DC1 dilution 1:10	0.2 % PCA MOPS2 MM*, 37 °C, 100 or 220 rpm, 20 or 100 mL in non-baffled 250-mL-flask, 6 h, inoculated from DC2 with starting OD_{600} of 0.05
PHB produc-tion	**Error! Referenc e source not found.**	*V. natriegens* parental strain (AF908-AF909) and ALE lineages (AF910-AF911, AF954-AF955, AF912-AF917) with plasmid-based PHB production system	0.1 % Glc MOPS2 MM* + kan, 30 °C, 300 rpm, 1 mL in 2.2-mL 96-deep-well-plate	0.1 % PCA + 0.1 % Glc MOPS2 MM* + kan, 37 °C, 300 rpm, 1 mL in 2.2-mL 96-deep-well-plate, 4 h, inoculated from OC	0.1 % PCA MOPS2 MM* + kan, 37 °C, 300 rpm, 1 mL in 2.2-mL 96-deep-well-plate, 3 h, inoculated from DC1 with	<u>Seed culture</u>: 0.1 % PCA MOPS2 MM* + kan, 37 °C, 220 rpm, 20 mL in non-baffled 250-mL-flask, until reaching late expo-nential phase, inoculated

				dilution 1:1000	starting OD_{600} 0.01	from DC2 with starting OD_{600} of 0.03<br><br><u>Production culture</u>:<br><br>0.1 % PCA MOPS2 MM* + kan + 200 µg/mL aTc, 37 °C, 220 rpm, 20 mL in non-baffled 250-mL-flask, up to 12 h, inoculated from seed culture dilution 1:5
cold storage survival	**Supplementary Figure** 3G	*V. natriegens* parental strain (AF614) and ALE lineages (AF616, AF824, AF624, AF828, AF830)	Streak out from cryostocks onto LBv2 gar plate, incubated at 7 °C overnight	NA	NA	Stored at 4 °C for up to 30 days, streaked out onto new LBv2 agar plate and incubated overnight at 37 °C
CFU count experi-ment	**Supplementary Figure 3H**	*V. natriegens* parental strain (AF614) and ALE lineage 5 (AF624)	LBv2, 30 °C, 220 rpm, 5 mL in glass tube	NA	NA	0.2 % PCA MOPS2 MM*, 37 °C, 220 rpm, 20 mL in non-baffled 250-mL-flask, up to 48 h, inoculated from OC dilution 1:1000<br><br>→ plated out 100 µL of 1:10^5 or 1:10^6

						dilution on agar plates for CFU count

Supplementary Table 4. Plasmids built in this study (excluding NT-CRISPR plasmids). Parts starting with pVC are from the Vnat Collection (Faber et al., 2025). Parts starting with pMC are from the Marburg Collection (Stukenberg et al., 2021).

Internal *E. coli* strain	Internal plasmid name	Description	Parts
AF0754	pAF1_72_lux-operon_pPcaU	Biosensor plasmid for intracellular PCA concentration	pVC0_1_02_5C1CLN, pVC0_2_50_P_{3B5}, pVC0_3_03_RB0030, pVC0_4_02_CDSlux-operon, pVC0_5_03_TB0015, pMC0_6_16_3C5CSN, pMC0_7_04_OpMB1-M, pMC0_8_18_Akan(Vn)_(sfgfp)(Vn)

Supplementary Table 5. Oligonucleotides used to assemble gRNA sequences for NT-CRISPR plasmids. The annealed oligonucleotides were cloned into pST_116 from Stukenberg et al., 2022. All sequences written in the 5' → 3' direction.

Target	Gene targeted by gRNA	Method	forward oligonucleotide		reverse oligonucleotide		Final plasmid
MFS	PN96_RS18620	Oligo annealing	GF2177_fwd	GTCCGTATTAGAACTATTTCGTGA	GF2178_rev	AAACTCACGAAATAGTTCTAATAC	pST116 + AF gDNA 3_MFS
TTT	PN96_RS05405	Oligo annealing	GF2185_fwd	GTCCAATTGTCGGCAATGTGCGAG	GF2186_rev	AAACCTCGCACATTGCCGACAATT	pST116 + AF gDNA 4_TTT
TRAP 1	PN96_RS18695	Oligo annealing	GF3068	GTCCCAGTGTTGCAAAATTCACCA	GF3069	AAACTGGTGAATTTTGCAACACTG	pST116 + TRAP1
TRAP 2	PN96_RS18750	Oligo annealing	GF3076	GTCCACAACTTCGAGCAAAAACAG	GF3077	AAACCTGTTTTTGCTCGAAGTTGT	pST116 + TRAP2
ABC transp.	PN96_RS18810	Oligo annealing	GF3084	GTCCTGGACGCAAGGAAATTACCG	GF3085	AAACCGGTAATTTCCTTGCGTCCA	pST116 + ABC

Supplementary Table 6. Oligonucleotides used to amplify individual homology flanks from *V. natriegens* ATCC14048 genome to build linear tDNA. All sequences are given in 5’ → 3’ direction. Abbreviations: HF - homology flank. Bolded sequences were overlapping sequences for fusion PCR.

**tDNA name**	**Genomic locus that HFs span across**	**5’ homology flank**				**3’ homology flank**			
		**Forward oligonucleotide**		**Reverse oligonucleotide**		**Forward oligonucleotide**		**Reverse oligonucleotide**	
AF tDNA 4_*mfs*	*mfs* (PN96_RS 18605 to PN96_RS 18630)	GF2179 fwd	GTACTTCTTTTTACACCTTAGATATCAG	GF2180 rev	**ATGTATTTCTAA**ACGCAGCTACCCCTTTTGAG	GF2181 rev	**GCGT**TTAGAAATACATTAACCCGGCACG	GF2182 rev	AATCGAAGGCTTCAGCCTGTG
AF tDNA 5_*ttt*	*ttt* (PN96_ RS05390 to PN96_ RS05425)	GF2187 fwd	TAATAAATTTGTTGTATACAAACATGTAAACG	GF2188 rev	**GTGAAC**AGTTTATACCCATGATTGACTCCTAAAAG	GF2189 rev	**GTATAAACT**GTTCACGTTCCTTATGTGAGTGAC	GF2190 rev	CATAATCACGAAACTTTCGTCGTTACG
AFtDNA_ TRAP1	*TRAP1* (PN96_RS 18695- 18700)	GF3062	CTCTTCCGCCATTTTCTTCGTG	GF3063	**CTATCCAGAAATGTCG**AATGTCCTCACGTTATTGATCTTATAAATAC	GF3064	**CAATAACGTGAGGACATTCGACATT**TCTGGATAGTATTGCC	GF3065	CCAGTCAATCGATCAAACTAAGCC
AFtDNA_ TRAP2	*TRAP2* (PN96_RS 18740- PN96_RS 18760)	GF3070	GGTTAAACGCGCCATTGACC	GF3071	**GAAATTCCGGAAGC**TCTGTGAACCTCCTGTTTCATGG	GF3072	**GGAGGTTCACAGA**GCTTCCGGAATTTCTCTGTCC	GF3073	GGTATCTTCAACTGATAAATTGGCG
AFtDNA_ABC	*ABC* (PN96_RS 18810- 18815)	GF3078	GCATTTCGCTCGAGTGCATC	GF3079	**GGGATTTCAGCTCTGC**CTATAACTCCTACTTGTGAATAATTCTC	GF3080	**GTAGGAGTTATAG**GCAGAGCTGAAATCCCGTTTTAC	GF3081	GCTGCTTTGGTAGATGCGC

Supplementary Table 7. Q5 PCR reaction mix.

**Reagent**	**Volume per reaction**
ddH2O	32.5 µL
5X Q5 Buffer	10 µL
10 µm Forward oligonucleotide	2.5 µL
10 µm Reverse oligonucleotide	2.5 µL
dNTPs	1 µL
Q5 Polymerase	0.5 µL
Template DNA	~10 ng (1 µL)

Supplementary Table 8. Standard Q5 PCR thermal cycler protocol.

Step	Temperature	Duration	Number of cycles
Initial denaturation step	98 °C	30 s	1
Denaturation	98 °C	10 s	25
Annealing	686 °C	10 s	
Extension	72 °C	30 s/kb	
Final Extension	72 °C	5 mins	1
Hold	16 °C	∞	1

Supplementary Table 9. **Splicing-by-overlap extension (SOE) thermal cycler protocol**. kB – kilo base pair. T_a – annealing temperature of primer.

Step	Temperature	Duration	Note	Cycles
initial denaturation step	98 °C	2 mins	30 s	1
denaturation	98 °C	10 s		15
annealing	70 °C	1 s	From this temperature, ramp down temperature at 0.1 °C/s to T_a	
annealing	68 °C	15 s	From this temperature, ramp up temperature at 0.2 °C/s to 72 °C	
extension	72 °C	30s/kb		
final Extension	72 °C	5 mins		1
hold	16 °C	∞		1

Supplementary Table 10. Oligonucleotides used to amplify edited locus in engineered strains after NT-CRISPR in colony PCR and for sequencing. All sequences written in the 5' → 3' direction.

Target	Locus tag (PN96_RS)	forward oligonucleotide		reverse oligonucleotide	
MFS	PN96_RS18605 to PN96_RS18625	GF2183 fwd	AACACTTCATCAGCGGGTTGG	GF2184 rev	CCGCTCCGTACTATCACG
TTT	PN96_RS05385 to PN96_RS05415)	GF2191 fwd	GTCGATCCACTAGGGTTCTTC	GF2192 rev	ATCTACCTTTTGCAGACATGTCG
TRAP1	PN96_RS18695-18700	GF3066	CGATCTGGTTGTATTTCGCTGG	GF3067	CATGCCTGAGACGAATAGTGC

TRAP2	PN96_RS18 740- PN96_RS18 760	GF3074	CGATCTTACGCTCCATCATCTTG	GF3075	CAATCTACGACAAACTGCTTCTC
ABC	PN96_RS18 810- 18815	GF3082	CGATCGTATGACTGACACTATTGAG	GF3083	CCTTGTTAAGCGGTCCAGATG

Supplementary Table 11. Parameters for plate reader protocol.

**Protocol**	**Luminescence measurement**	**OD600 measurement**
temperature	37 °C	37 °C
duration	24 hours	24 hours
interval of measurement	10 min	10 min
duration of shaking	8 min 20 s	8 min 20 s
duration of measurement	1 min 40 s	1 min 40 s
shaking mode	Continuous and/orbital	Continuous and/orbital
orbital speed	fast	fast
orbital Frequency	807 cpm (1 mm)	807 cpm (1 mm)
wavelength	Emission hole	Absorbance 600 nm
read speed	Normal	Normal
gain	255	NA
optics position	Top	NA
integration time	1 s	NA
read height	4.5 mm	NA

Supplementary Table 12. Gradient composition of the mobile phase for PCA flow cytometry.

**Time**	**100 % acetonitrile** [% of mobile phase]	**0.1 % trifluoroacetic acid** [% of mobile phase]
0 min	0	100
10 min	50	50
20 min	100	0

Supplementary data on culture conditions and growth on PCA

Supplementary Table 13. Growth data for *V. natriegens* parental strain and 5-transporter-knockout strain (d5) on 0.1 % PCA in MOPS2 minimal media with 2.29 % NaCl. 20 mL of liquid cultures were incubated in a non-baffled 250-mL flasks at 37 °C at 220 rpm. Data was used for metabolic model predictions (**Error! Reference source not found.**). D5 – *V. natriegens* strain with 5 parallel transporter knockouts.

time [h]	od600, parental, replicate a	od600, parental, replicate b	od600, parental, replicate c	od600, d5, replicate a	od600, d5, replicate b	od600, d5, replicate c
0.0	0.065	0.069	0.053	0.032	0.039	0.039
0.5	0.067	0.090	0.052	0.079	0.094	0.072
1.0	0.115	0.154	0.104	0.146	0.135	0.103
1.5	0.131	0.208	0.137	0.208	0.223	0.171
2.0	0.286	0.438	0.336	0.288	0.336	0.260
2.5	0.466	0.698	0.568	0.512	0.690	0.558
3.0	0.995	1.400	1.095	1.125	1.455	1.175
3.5	1.585	1.820	1.575	1.670	1.785	1.995
4.0	2.075	1.990	2.075	2.015	1.770	1.765
4.5	1.980	1.905	2.235	1.625	1.970	2.050
5.0	1.945	1.890	2.015	1.540	1.705	1.860
5.5	2.000	1.990	1.910	1.640	1.765	1.680

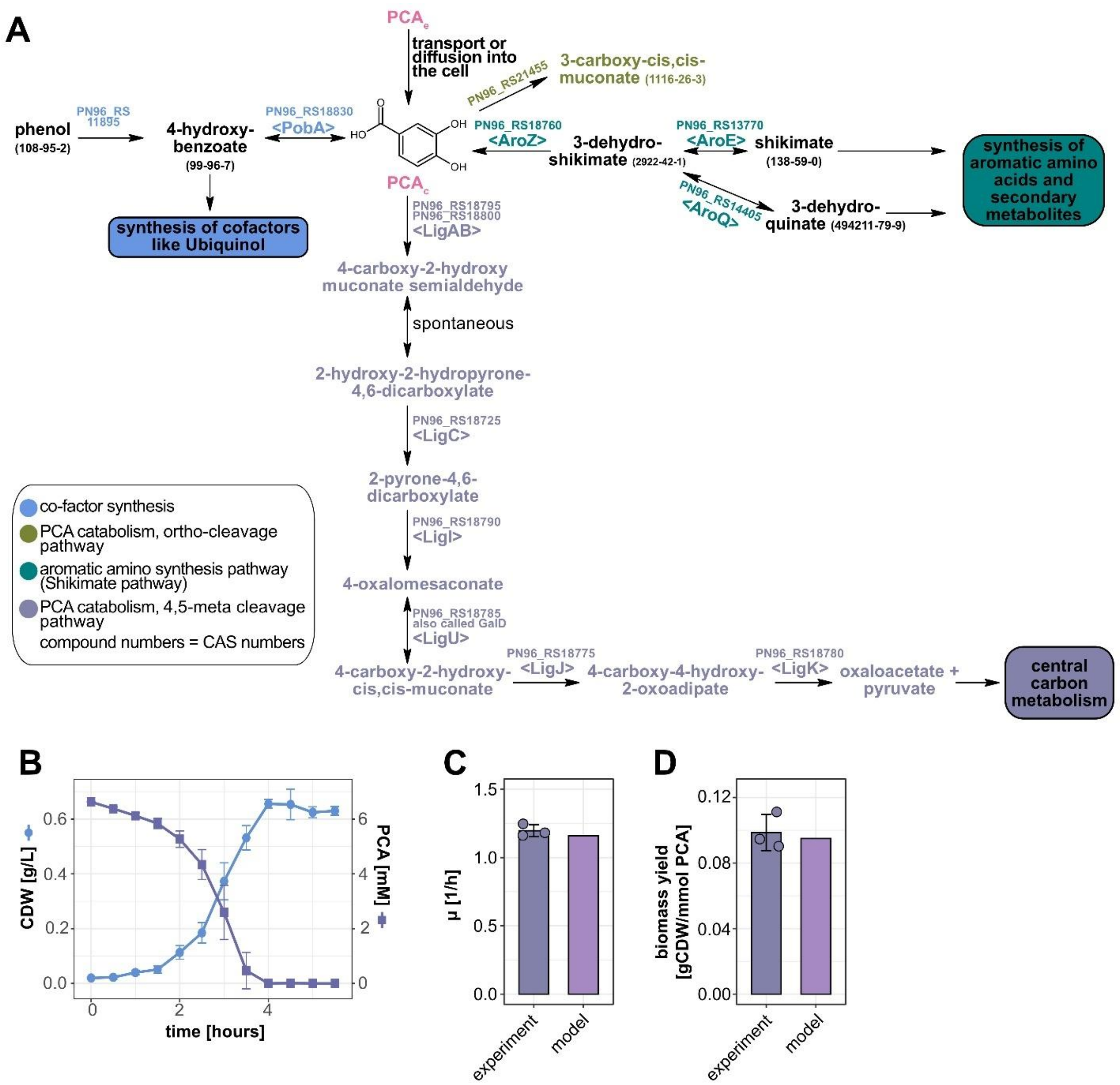

Supplementary Figure 1. Additional information on growth behaviour of different *V. natriegens* strains on PCA and glucose. A) Overview of predicted, extended PCA metabolism in *V. natriegens*, highlighting its integration into central carbon metabolism, amino acid biosynthesis, and cofactor production. The presence of enzymes in *V. natriegens* and the direction of reaction arrows are based on KEGG annotations. B) PCA consumption and cell dry weight (CDW) in 20 mL MOPS2 minimal media in 250-mL non-baffled flasks over 5.5 hours for *V. natriegens* parental strain. In contrast to Figure 2C, where cells were pre-cultured on PCA supplemented with glucose, cultures in panel B were pre-cultured on PCA as sole carbon source to match PHB production conditions. The shift from a PCA pre-culture to a PCA main culture likely generates less initial ROS stress, which may explain the shorter lag phase compared to Figure 2C. C) Growth rate and D) biomass yield for measured and modelled *V. natriegens* shake flasks cultures on 0.1 % PCA in MOPS2 minimal media with 2.29 % NaCl at pH 7.2. Raw data in

Supplementary Table 13.

Supplementary data on transporter knockouts

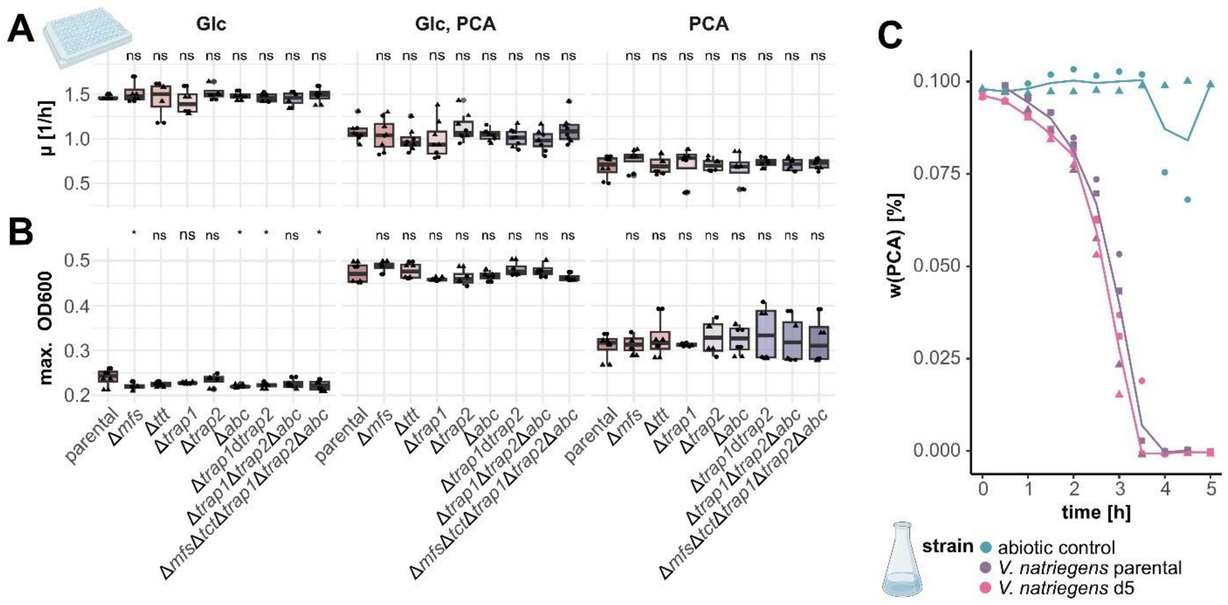

Supplementary Figure 2. Additional information on growth behaviour of transporter deletion strains. Detailed description of deleted transporters can be found in

Supplementary Table 14. A) Growth rate and B) maximal optical density (OD_{600}) from different *V. natriegens* strains carrying the plasmid-based PCA-biosensor, parental strain and strains with knockouts of different endogenous PCA transporter candidates. OD_{600} was measured in 96-well plates. Shapes represent the two biological replicates, measured on two independent days. Statistical analysis was performed with an unpaired two-way t-test (*p < 0.05, **p < 0.01, ***p < 0.001, ****p < 0.0001). C) PCA uptake for *V. natriegens* parental strain and 5-transporter knockout strain (d5) grown in shake flasks. Data represents three technical replicates, represented by different shapes of data points. Raw data can be found in

Supplementary Table 13.

Supplementary Table 14. **Endogenous transporter candidates for PCA uptake in *V. natriegens*.**

Abbrev.	Name	Genetic locus	Genetic environment	Reason for investigation
MFS	major facilitator superfamily transporter	PN96_RS18620, chromosome 2	genes for catabolism of aromatic compounds (benzoate to 3-oxoadipate enol-lactone), PN96_RS18585-PN96_RS18615	relatively high sequence identity to known PCA and TPA transporters (34% id. to PcaK of *Pseudomonas putida*,(Nichols and Harwood, 1997; Pernstich et al., 2014) 34 % id. to PmdK of *Comomonas testosteroni*,(Kamimura et al., 2010) 31 % id. to TpaK of *Rhodococcus jostii*(Patrauchan et al., 2005))
TTT	tripartite tricarboxy-late transporter	PN96_RS05395 to PN96_RS05410, chromosome 1	not related to catabolism of aromatic compounds	24 % sequence identity to known TPA transporter (24% id. to TphC of *Comomonas testosteroni*(Hosaka et al., 2013))
Trap trans-porter 1	TAXI family TRAP transporter	PN96_RS18695 to PN96_RS18700, chromosome 2	upstream of PCA 4,5-meta cleavage pathway operon	involvement in the degradation of several benzoate derivatives and lignin-derived aromatic compounds(Rosa et al., 2018; Salmon et al., 2013)
Trap trans-porter 2	DctP family TRAP transporter	PN96_RS18740 to PN96_RS18755, chromosome 2	upstream of PCA 4,5-meta cleavage pathway operon	involvement in the degradation of several benzoate derivatives and lignin-derived aromatic compounds(Rosa et al., 2018; Salmon et al., 2013)
TonB-depen-dent trans-porter	TonB-dependent siderophore ABC transporter	PN96_RS18805 to PN96_RS18810, chromosome 2	downstream of PCA 4,5-meta cleavage pathway operon	described in literature as being involved in the uptake and catabolism of aromatic compounds(Fujita et al., 2019)

Supplementary data on ALE lineages and media optimization

Improvements in ROS-damage repair

PCA-induced ROS can damage bacterial cells by oxidising proteins, lipids, DNA, and RNA, making a diverse array of repair systems indispensable (Ajiboye et al., 2017; Galano and Pérez-González, 2012; Imlay et al., 1988; Li et al., 2011; Lushchak, 2001; Seixas et al., 2022; Tan et al., 2019). ROS dismantle and destabilize the peptidoglycan layer, increasing the need for enhanced cell wall repair and maintenance in the presence of PCA (Cui et al., 2018; Tang et al., 2025; Yusupov et al., 2013, 2012). Against this background, we hypothesize that mutations in genes related to peptidoglycan synthesis and regulation, such as RodA (Emami et al., 2017), D-amino acid transaminase (Pucci et al., 1995; Shilova et al., 2023), penicillin-binding protein 2 (Pinho et al., 2001), MltC (Dik et al., 2018), MurA (Brown et al., 1995), and MreD (Wachi et al., 1989), could have accumulated in our ALE lineages as an adaptive response to elevated ROS-induced cell wall damage. Hence, adaptive mutations in *V. natriegens* during growth on PCA may extend beyond direct ROS mitigation and include broader cellular damage repair mechanisms such as those on peptidoglycan.

We also identified mutations potentially linked to enhanced repair of ROS-induced protein damage. ROS can cause proteotoxic stress, leading to the aggregation of misfolded proteins in the cytosol (Schramm et al., 2020). In ALE replicate 6.2, we detected a silent mutation in the adaptor protein YjbH (PN96_RS12650), introducing a more frequently used codon (GTC→GTT) (Lee et al., 2016). Under low-stress conditions, YjbH directs the ClpXP protease to the transcriptional regulator Spx, facilitating its degradation (Engman and von Wachenfeldt, 2015; Martinez, 2023). However, during proteotoxic stress, the YjbH protein itself aggregates, preventing Spx degradation and resulting in Spx accumulation (Engman and von Wachenfeldt, 2015; Martinez, 2023). In turn, Spx accumulation activates genes involved in proteotoxic and oxidative stress responses (Engman and von Wachenfeldt, 2015; Martinez, 2023). As part of this activated proteotoxic stress response, chaperones assist in protein refolding and degradation (Hartl and Hayer-Hartl, 2002; Martinez, 2023). In ALE lineage 5, we observed an amino acid substitution (T209M) in an FKBP-type peptidyl-prolyl cis-trans isomerase (PN96_RS14905), a chaperone that promotes the refolding of partially denatured proteins (Jo et al., 2015). Together, the mutations identified in YjbH and the FKBP-type chaperone suggest that adaptive evolution under PCA-induced oxidative stress could also enhance mechanisms mitigating proteotoxic stress in *V. natriegens*. To our knowledge, functionally similar orthologs of YjbH have been associated with oxidative stress responses in Firmicutes including *Bacillus subtilis* (Martinez, 2023; Nakano et al., 2003) and *Staphylococcus aureus* (Engman et al., 2012; Paudel et al., 2021), but have not been reported in Proteobacteria. In contrast, FKBP-type peptidyl-prolyl isomerase homologues have been described in *Vibrio anguillarum* (Jo et al., 2015). Together, these findings suggest that both proteins might serve conserved roles in oxidative stress management in *V. natriegens*, although further investigation is needed to confirm this hypothesis.

We also examined a third aspect of improved ROS damage repair by looking at adaptive mutations associated with DNA repair mechanisms. Among these was a silent mutation in the excinuclease ABC subunit UvrA (PN96_RS00290), introducing a less frequently used codon (CCA→CCG) in ALE replicate 6.2. This mutation might influence the translation and/or folding efficiency of UvrA, which is involved in DNA lesion recognition under ROS-induced stress (Pakotiprapha et al., 2012). Taken together, our findings hint that adaptation in *V. natriegens* to oxidative stress may

extend beyond direct ROS mitigation to include pathways supporting cellular repair and maintenance. However, since the mutations related to DNA and protein repair were identified only in single ALE lineages, their occurrence could also be coincidental and not be attributable to PCA-specific selection pressures in every case.

Adaptation to hyperosmotic laboratory conditions in *V. natriegens*

Selection pressures during adaptive laboratory evolution are multifactorial and extend beyond carbon source utilization or compound toxicity. In the context of non-PCA-related selection pressures, we identified *bccT1* (PN96_RS06115) in ALE lineage 5 as a target of adaptive mutations associated with osmotic stress. Bacteria mitigate osmotic stress in hyperosmotic conditions by accumulating compatible solutes that help regulate cellular hydration and turgor pressure (Ziegler et al., 2010). Betaine/choline/carnitine (BCCT) family transporters are bidirectional symporters that mediate the uptake of these compatible solutes in symport with two H^+ or Na^+ ions (Gregory et al., 2020; Ongagna-Yhombi et al., 2014; Ziegler et al., 2010). *V. natriegens* encodes two *bccT* genes on chromosome 1 and five on chromosome 2, which mitigate osmotic stress through the uptake of environmental osmolytes such as ectoine (Politan et al., 2025; Thomas et al., 2025). (Politan et al., 2025) reported that disrupted BCCT1 function can serve as an adaptive strategy to osmotic stress in MOPS2 minimal medium with no external osmolytes, leading to enhanced sodium tolerance independent of carbon metabolism. Further, ectoine has stabilizing properties against protein oxidation in the presence of H_2O_2 (Andersson et al., 2000). Similarly, we hypothesize that the *bccT1* disruption observed in ALE lineage 5 might reduce sodium influx and osmolyte efflux, increasing the intracellular concentration of ectoine as osmo- and redox-protectant. This confers improved sodium tolerance and osmotic stress resilience at sodium concentrations exceeding *V. natriegens*' optimum (1.5 % NaCl, (Forsten et al., 2024) – independent of PCA metabolism.

Further convergent mutations in independent *V. natriegens* cell lines

Two additional genomic loci were repeatedly targeted in multiple independent ALE lineages, suggesting potential adaptive advantages or functional relevance across distinct evolutionary trajectories. One of these loci corresponded to the chromosomal replication initiator protein DnaA (PN96_RS13495) (Zakrzewska-Czerwińska et al., 2007), which carried an amino acid substitution in both ALE lineages 2 and 5. The role of this reoccurring mutations remains unclear, as we could not directly link them to PCA-induced selection pressures or observed phenotypes without further investigation.

A second recurrent mutation was identified in the gene encoding a four-helix bundle protein (PN96_RS12325), located within an operon associated with Vi polysaccharide biosynthesis (Hu et al., 2016). This gene was disrupted by the introduction of a premature stop codon in ALE replicate 2.1 and strain 5, likely impairing its function. Overall, genes potentially involved in quorum sensing (PN96_RS03335) (Politan et al., 2025), biofilm formation (PN96_RS03335, 08205; compare NCBI entry for "DUF1190 domain-containing protein YgiB") (Beloin et al., 2004; Politan et al., 2025), and polysaccharide biosynthesis (PN96_RS12325)(Hu et al., 2016) were mutated in three independent cell lines. Notably, the σ^{54}-dependent transcriptional regulator (PN96_RS03335) accumulated several amino acid substitutions in ALE replicate 4.2, which

probably impaired its regulatory function. The σ^{54}-dependent regulator (homologous to *luxO*) is encoded upstream of *luxU (*PN96_RS03340) on chromosome 1, a key component in controlling biofilm formation at low cell density and planktonic growth at high cell density (Politan et al., 2025). Hence, serial dilutions, as performed during ALE, force cells to enter the low cell density state, causing transcriptomic changes and prolonged lag phases. Interestingly, loss of *luxU* function has also been reported as a common adaptive mutation in *V. natriegens* evolved on MOPS2 minimal medium with sodium acetate as the sole carbon source (Politan et al., 2025). In conclusion, mutations affecting *luxU*-related regulatory pathways could represent favourable adaptations to ALE in liquid media, where planktonic cells would have growth advantage, potentially leading to shorter lag phases after dilution.

Supplementary figure 3 on observations and phenotypes around ALE

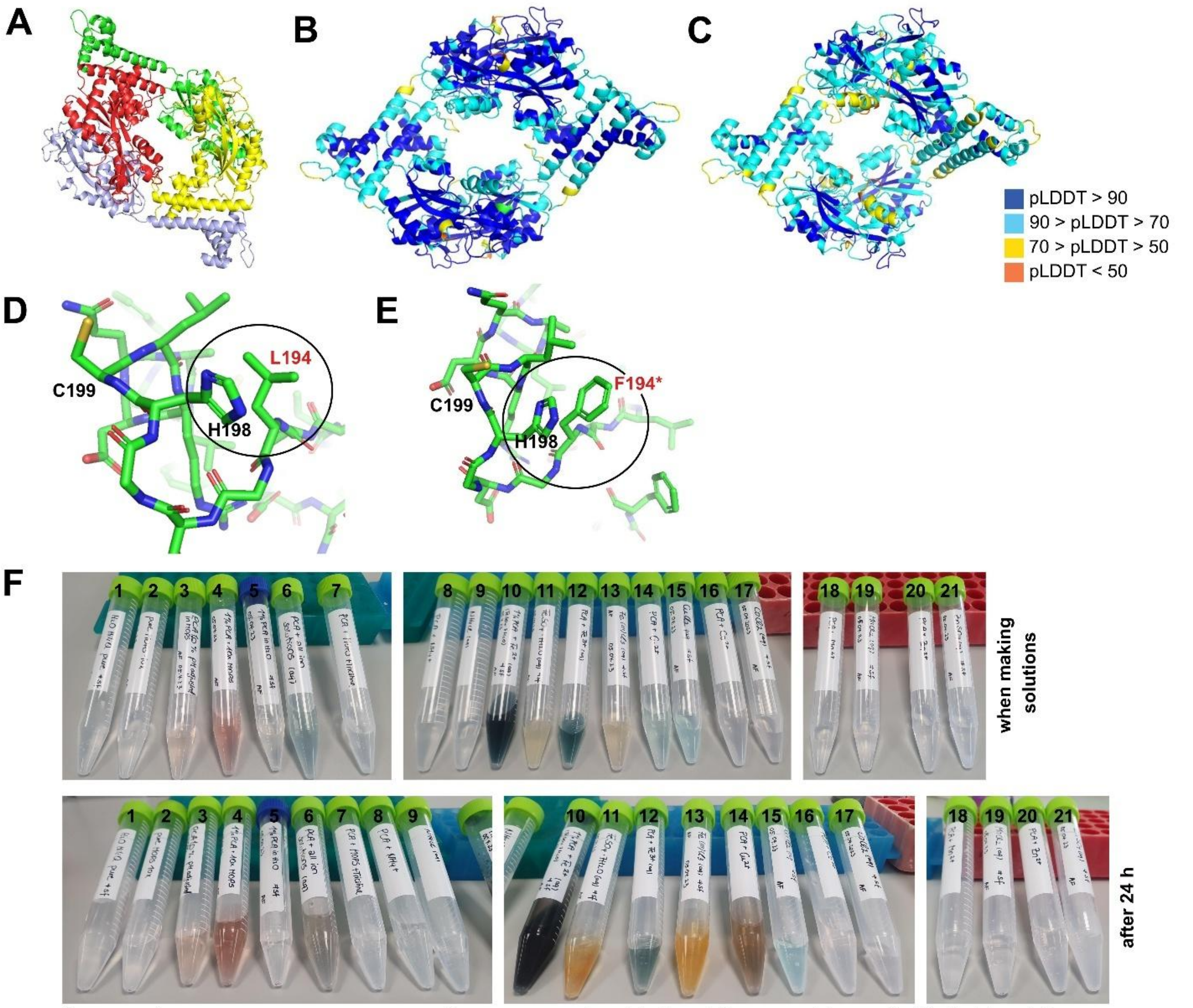

1 - pure water
2 - pure MOPS minimal media
3 - 1 % PCA in MOPS minimal media
4 - 1 % in MOPS 10x stock solution
5 - 1 % PCA in water
6 - PCA in mix of all MOPS ion solutions
7 - PCA in tricine solution
8 - PCA in ammonium chloride solution
9 - pure ammonium chloride solution
10 - PCA in Fe(II) sulphate solution
11 - pure Fe(II) sulphate solution
12 - PCA in Fe(III) chloride solution
13 - pure Fe(III) chloride solution
14 - PCA in Cu(II) chloride solution
15 - pure Cu(II) chloride solution
16 - PCA in Co(II) chloride solution
17 - pure Co(II) chloride solution
18 - PCA in Mn(II) chloride solution
19 - pure Mn(II) chloride solution
20 - PCA in Zn(II) sulphate solution
21 - pure Zn(II) sulphate solution

all solutions are aqueous solutions (water = solvent)

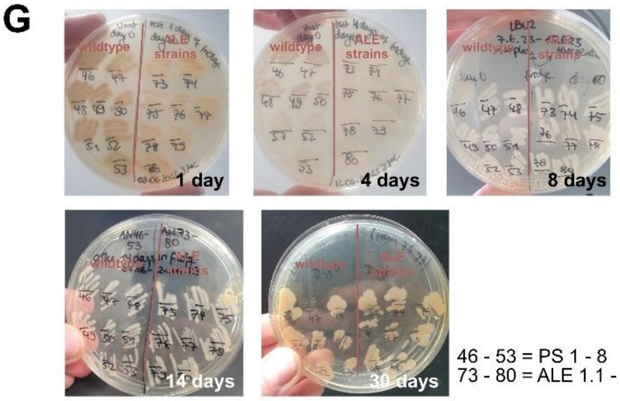

46 - 53 = PS 1 - 8
73 - 80 = ALE 1.1 - 8.1

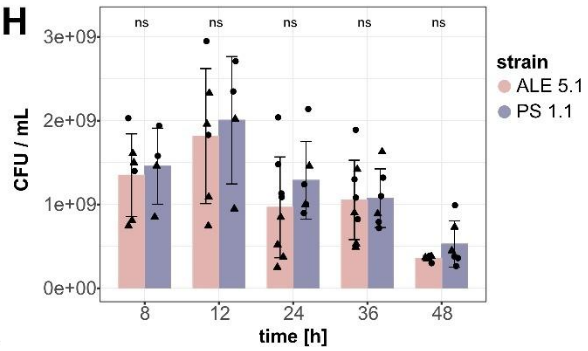

Supplementary Figure 3. Additional comparison of phenotypes between *V. natriegens* parental strain and ALE lineages. A) Parental OxyR with different colouring of all 4 constituent chains of the tetrameric structure. B) Structural prediction for *V. natriegens*' parental OxyR with pLDDT scores colouring. C) Structural prediction for *V. natriegens*' OxyR mutant with pLDDT scores colouring. pLDDT scores colouring applies to B and C. D) Binding pocket for H_2O_2 in parental OxyR, with C199 and neighbouring residues highlighted. E) Binding pocket for H_2O_2 in mutant OxyR, with C199 and neighbouring residues highlighted. The predicted structure shows the proximity of the aromatic L194F mutation. F) Comparing colour of different salt solutions with and without addition of PCA, directly after mixing and after incubation at room temperature for 24 h. All chosen salts and tricine are trace elements and ingredients of the MOPS2 minimal media used for cultivation of *V. natriegens* in this study, in various concentrations. G) Comparison of survival at cold temperature (4 °C) for up to 30 days between biological replicates of *V. natriegens* parental strain (PS, number 46-53) and all eight ALE lineages (number 73-80). The pictures were taken after transfer of cells from the cold storage plate at 4 °C onto fresh LBv2 plates, incubated at 37 °C overnight. H) Comparison of colony forming units (CFU) between parental strain (PS) and best-performing ALE lineage (ALE 5.1) after survival in liquid MOPS2 minimal media with 0.2 % PCA for up to 48 h at 37 °C (220 rpm). Colonies were plated out on LBv2 complex media to count CFU. Data from two biological replicates was taken on two individual days. Statistical analysis in panel was performed with an unpaired two-way t-test (*p < 0.05, **p < 0.01, ***p < 0.001, ****p < 0.0001).

Detailed list of all mutations of ALE lineages

Supplementary Table 15. Accumulated mutations in eight independent strains of *V. natriegens* during adaptive laboratory evolution (ALE) for 60 days on 0.2 % PCA in MOPS2 minimal media (NaCl = 2.29 %). (u) or (d) in the locus tag means that the mutation is upstream or downstream of the given locus tag, respectively. str. – strain, chr – chromosome.

str	locus tag	gene	product	bp change	codon change	aa change	protein effect	CDS position	chr
**WT 1.1**	PN96_RS00005 (u)			-A			mutation not in CDS	none	1
**ALE 1.1**	PN96_RS02575		Dyp-type peroxidase	C -> T	TCT -> TTT	S -> F	substitution	167	1
**ALE 1.1**	PN96_RS03780		circularly permuted type 2 ATP-grasp protein	G -> T	AAC -> AAA	N -> K	substitution	42	1
**ALE 1.1**	PN96_RS09070 (u)		upstream of HU family DNA−binding protein (histone-like protein)	(A)8 -> (A)7			mutation not in CDS	none	1
**ALE 1.1**	PN96_RS12305		hypothetical protein	T -> A	CAA -> CAT	Q -> H	substitution	195	1
**ALE 1.1**	PN96_RS15030	*oxyR*	DNA-binding transcriptional regulator OxyR	G -> C	ATG -> ATC	M -> I	substitution	690	1
**ALE 1.1**	PN96_RS15335	*fhuB*	Fe^{3+}-hydroxamate ABC transporter permease FhuB for uptake of Fe^{3+}	C -> T			truncation	499	2
**ALE 1.1**	PN96_RS18220		MarR family transcriptional regulator, upstream of HlyD family secretion protein for secretion of the RTX hemolytic toxin HlyA	G -> T			truncation	199	2
**ALE 1.2**	PN96_RS02575		Dyp-type peroxidase	C -> T	TCT -> TTT	S -> F	substitution	167	1
**ALE 1.2**	PN96_RS03780		circularly permuted type 2 ATP-grasp protein	G -> T	AAC -> AAA	N -> K	substitution	42	1
**ALE 1.2**	PN96_RS09070 (u)		upstream of HU family DNA−binding protein (histone-like protein)	(A)8 -> (A)7			mutation not in CDS	none	1

**ALE 1.2**	PN96_RS1 2305		hypothetical protein	T -> A	CAA -> CAT	Q -> H	substitution	195	1
**ALE 1.2**	PN96_RS1 5030	*oxyR*	DNA-binding transcriptional regulator OxyR	G -> C	ATG -> ATC	M -> I	substitution	690	1
**ALE 1.2**	PN96_RS1 5335	*fhuB*	Fe^{3+}-hydroxamate ABC transporter permease FhuB for uptake of Fe^{3+}	C -> T			truncation	499	2
**ALE 2.1**	PN96_RS0 0005 (u)			minus aa			mutation not in CDS	none	1
**ALE 2.1**	PN96_RS0 5225		S8 family serine peptidase, serine protease	G -> T	GTC -> TTC	V -> F	substitution	1651	1
**ALE 2.1**	PN96_RS0 9525		transcriptional regulator, upstream of regulatory protein ToxS	A -> G	ACA -> GCA	T -> A	substitution	271	1
**ALE 2.1**	PN96_RS1 2325		four helix bundle protein	(G)2 -> (G)3			frame shift through insertion	370	1
**ALE 2.1**	PN96_RS1 3495	*dnaA*	chromosomal replication initiator protein DnaA	G -> A	GCA -> GTA	A -> V	substitution	695	1
**ALE 2.1**	PN96_RS1 5030	*oxyR*	DNA-binding transcriptional regulator OxyR	C -> A	CCT -> ACT	P -> T	substitution	307	1
**ALE 2.2**	PN96_RS0 0005 (u)			minus aa			mutation not in CDS	none	1
**ALE 2.2**	PN96_RS0 0785(d)		downstream of lytic murein transglycosy-lase MltC (peptidoglycan cleavage)	C -> T			mutation not in CDS	none	1
**ALE 2.2**	PN96_RS0 5225		S8 family serine peptidase, serine protease	G -> T	GTC -> TTC	V -> F	substitution	1651	1
**ALE 2.2**	PN96_RS0 9525		transcriptional regulator, upstream of regulatory protein ToxS	A -> G	ACA -> GCA	T -> A	substitution	271	1
**ALE 2.2**	PN96_RS1 3495	*dnaA*	chromosomal replication initiator protein DnaA	G -> A	GCA -> GTA	A -> V	substitution	695	1
**ALE 2.2**	PN96_RS1 5030	*oxyR*	DNA-binding transcriptional regulator OxyR	C -> A	CCT -> ACT	P -> T	substitution	307	1

**ALE 3.1**	PN96_RS0 0005 (u)			minus aa			mutation not in CDS	none	1
**ALE 3.1**	PN96_RS1 0055	*mrdA*	penicillin-binding protein 2, involved in cell wall cross-linking	T -> G	ATC -> AGC	I -> S	substitution	1355	1
**ALE 3.1**	PN96_RS1 5030	*oxyR*	DNA-binding transcriptional regulator OxyR	C -> T	GCA -> GTA	A -> V	substitution	698	1
**ALE 3.1**	PN96_RS1 8760		sugar phosphate isomerase/epimerase and 4-hydroxyphenylpyruvate domain-containing protein	G -> T	GGG -> TGG	G -> W	substitution	202	2
**ALE 3.1**	PN96_RS1 8760		sugar phosphate isomerase/epimerase and 4-hydroxyphenylpyruvate domain-containing protein	A -> G	CTA -> CTG		silent	207	2
**ALE 3.1**	PN96_RS1 8760		sugar phosphate isomerase/epimerase and 4-hydroxyphenylpyruvate domain-containing protein	CA -> TG	CCA -> CTG	P -> L	substitution	209	2
**ALE 3.1**	PN96_RS1 8760		sugar phosphate isomerase/epimerase and 4-hydroxyphenylpyruvate domain-containing protein	A -> T	TTA -> TTT	L -> F	substitution	219	2
**ALE 3.1**	PN96_RS2 3035		YjiH family protein, putative arginine transporter (argw) family	C -> A	ATC -> ATA		silent	72	2
**ALE 3.2**	PN96_RS0 8945	*vmeF*	multidrug efflux RND transporter permease subunit VmeF	minus ATCAC CAGA			deletion without frame shift	1029	1
**ALE 3.2**	PN96_RS1 0060	*rodA*	rod shape-determining protein RodA, involved in peptidoglycan synthesis as glycosyltransferase	C -> A	CGT -> AGT	R -> S	substitution	619	1
**ALE 3.2**	PN96_RS1 5030	*oxyR*	DNA-binding transcriptional regulator OxyR	C -> T	GCA -> GTA	A -> V	substitution	698	1

**ALE 3.2**	PN96_RS23035		YjiH family protein, putative arginine transporter (argw) family	C -> A	ATC -> ATA		silent	72	2
**ALE 4.1**	PN96_RS00005 (u)			minus aa			mutation not in CDS	none	1
**ALE 4.1**	PN96_RS00405	*mreD*	rod shape-determining protein MreD	G -> T	GTT -> TTT	V -> F	substitution	31	1
**ALE 4.1**	PN96_RS08205		DUF1190 domain-containing protein, YgiB induced upon biofilm formation compared to planktonic growth	C -> A	GGT -> GTT	G -> V	substitution	512	1
**ALE 4.1**	PN96_RS09525 (u)		upstream of transcriptional regulator regulatory upstream of protein ToxS	(T)7 -> (T)8			mutation not in CDS	none	1
**ALE 4.1**	PN96_RS15030	*oxyR*	DNA-binding transcriptional regulator OxyR	G -> A	GCA -> ACA	A -> T	substitution	631	1
**ALE 4.1**	PN96_RS18735 (u)			G -> A			mutation not in CDS	none	2
**ALE 4.2**	PN96_RS03335		sigma-54-dependent Fis family transcriptional regulator	T -> A	CAT -> CAA	H -> Q	substitution	114	1
**ALE 4.2**	PN96_RS03335		sigma-54-dependent Fis family transcriptional regulator	C -> A	CGT -> AGT	R -> S	substitution	115	1
**ALE 4.2**	PN96_RS03335		sigma-54-dependent Fis family transcriptional regulator	AT -> CA	ATT -> CAT	I -> H	substitution	118	1
**ALE 4.2**	PN96_RS03335		sigma-54-dependent Fis family transcriptional regulator	C -> A	CCC -> CCA		silent	123	1
**ALE 4.2**	PN96_RS03335		sigma-54-dependent Fis family transcriptional regulator	C -> G	CTT -> GTT	L -> V	substitution	127	1
**ALE 4.2**	PN96_RS03335		sigma-54-dependent Fis family transcriptional regulator	AT -> CC	ATT -> CCT	I -> P	substitution	130	1
**ALE 4.2**	PN96_RS03335		sigma-54-dependent Fis family transcriptional regulator	A -> T	GAT -> GTT	D -> V	substitution	140	1
**ALE 4.2**	PN96_RS03335		sigma-54-dependent Fis family transcriptional regulator	C -> A	CGT -> AGT	R -> S	substitution	145	1
**ALE 4.2**	PN96_RS03335		sigma-54-dependent Fis family transcriptional regulator	G -> T	ATG -> ATT	M -> I	substitution	159	1

**ALE 4.2**	PN96_RS0 3335		sigma-54-dependent Fis family transcriptional regulator	G -> C	ACG -> ACC		silent	162	1
**ALE 4.2**	PN96_RS0 3335		sigma-54-dependent Fis family transcriptional regulator	G -> T	ATG -> ATT	M -> I	substitution	168	1
**ALE 4.2**	PN96_RS0 3335		sigma-54-dependent Fis family transcriptional regulator	CAT -> GAA	CAT -> GAA	H -> E	substitution	178	1
**ALE 4.2**	PN96_RS0 3335		sigma-54-dependent Fis family transcriptional regulator	G -> A	GCA -> ACA	A -> T	substitution	181	1
**ALE 4.2**	PN96_RS0 3335		sigma-54-dependent Fis family transcriptional regulator	G -> C	GTG -> CTG	V -> L	substitution	184	1
**ALE 4.2**	PN96_RS1 5030	*oxyR*	DNA-binding transcriptional regulator OxyR	G -> A	GCA -> ACA	A -> T	substitution	631	1
**ALE 4.2**	PN96_RS1 8735 (u)			G -> A			mutation not in CDS	none	2
**ALE 5.1**	PN96_RS0 0005 (u)			minus aa			mutation not in CDS	none	1
**ALE 5.1**	PN96_RS0 1030	*rseB*	sigma-E factor regulatory protein RseB	G -> C	GCC -> CCC	A -> P	substitution	208	1
**ALE 5.1**	PN96_RS0 6115		BCCT family transporter, osmosensory and osmoregulatory properties	C -> T			truncation	1013	1
**ALE 5.1**	PN96_RS1 2325		four helix bundle protein	(G)2 -> (G)3			frame shift through insertion	370	1
**ALE 5.1**	PN96_RS1 3495	*dnaA*	chromosomal replication initiator protein DnaA	G -> C	CAC -> CAG	H -> Q	substitution	942	1
**ALE 5.1**	PN96_RS1 4905	*fkpA*	FKBP-type peptidyl-prolyl cis-trans isomerase, chaperon for protein folding	C -> T	ACG -> ATG	T -> M	substitution	626	1
**ALE 5.1**	PN96_RS1 5030	*oxyR*	DNA-binding transcriptional regulator OxyR	G -> T	GGT -> TGT	G -> C	substitution	304	1
**ALE 5.2**	PN96_RS0 1030	*rseB*	sigma-E factor regulatory protein RseB	G -> C	GCC -> CCC	A -> P	substitution	208	1
**ALE 5.2**	PN96_RS0 6115		BCCT family transporter, osmosensory and osmoregulatory properties	C -> T			truncation	1013	1

**ALE 5.2**	PN96_RS1 2325		four helix bundle protein	(G)2 -> (G)3			frame shift through insertion	370	1
**ALE 5.2**	PN96_RS1 3495	*dnaA*	chromosomal replication initiator protein DnaA	G -> C	CAC -> CAG	H -> Q	substitution	942	1
**ALE 5.2**	PN96_RS1 4905	*fkpA*	FKBP-type peptidyl-prolyl cis-trans isomerase, chaperon for protein folding	C -> T	ACG -> ATG	T -> M	substitution	626	1
**ALE 5.2**	PN96_RS1 5030	*oxyR*	DNA-binding transcriptional regulator OxyR	G -> T	GGT -> TGT	G -> C	substitution	304	1
**ALE 6.1**	PN96_RS0 0555	*murA*	UDP-N-acetylglucosamine 1-carboxyvinyltransferase, catalyses the first step in peptidoglycan biosynthesis	A -> G	AAC -> AGC	N -> S	substitution	944	1
**ALE 6.1**	PN96_RS1 5030	*oxyR*	DNA-binding transcriptional regulator OxyR	G -> A	GCA -> ACA	A -> T	substitution	631	1
ALE 6.1	PN96_RS1 8760		sugar phosphate isomerase/epimerase and 4-hydroxyphenylpyruvate domain-containing protein	G -> T	GAA -> TAA	E -> *	truncation	88	2
**ALE 6.2**	PN96_RS0 0005 (u)			minus aa			mutation not in CDS	none	1
**ALE 6.2**	PN96_RS0 0290	*uvrA*	excinuclease ABC subunit UvrA	T -> C	CCA -> CCG		silent	567	1
**ALE 6.1**	PN96_RS0 0555	*murA*	UDP-N-acetylglucosamine 1-carboxyvinyltransferase, catalyses the first step in peptidoglycan biosynthesis	A -> G	AAC -> AGC	N -> S	Substitution	944	1
**ALE 6.2**	PN96_RS1 2650		YjbH domain-containing protein, adapter in regulated protein degradation	G -> A	GTC -> GTT		silent	462	1
**ALE 6.1**	PN96_RS1 5030	*oxyR*	DNA-binding transcriptional regulator OxyR	G -> A	GCA -> ACA	A -> T	Substitution	631	1
**ALE 6.2**	PN96_RS1 8760		sugar phosphate isomerase/epimerase and 4-	G -> T	GAA -> TAA	E -> *	truncation	88	2

			hydroxyphenylpyruvate domain-containing protein						
**ALE 7.1**	PN96_RS0 1145	*rpoS*	RNA polymerase sigma factor RpoS, regulating oxidative stress response	T -> A	ATC -> AAC	I -> N	substitution	368	1
**ALE 7.1**	PN96_RS0 9525		transcriptional regulator, upstream of regulatory protein ToxS	C -> T	TCT -> TTT	S -> F	substitution	842	1
**ALE 7.1**	PN96_RS1 5030	*oxyR*	DNA-binding transcriptional regulator OxyR	A -> G	ATA -> ATG	I -> M	substitution	294	1
**ALE 7.2**	PN96_RS0 1145	*rpoS*	RNA polymerase sigma factor RpoS, regulating oxidative stress response	T -> A	ATC -> AAC	I -> N	substitution	368	1
**ALE 7.2**	PN96_RS0 6505		hypothetical protein	+C			frame shift through insertion	925	1
**ALE 7.2**	PN96_RS0 9525		transcriptional regulator, upstream of regulatory protein ToxS	C -> T	TCT -> TTT	S -> F	substitution	842	1
**ALE 7.2**	PN96_RS1 5030	*oxyR*	DNA-binding transcriptional regulator OxyR	A -> G	ATA -> ATG	I -> M	substitution	294	1
**ALE 7.2**	PN96_RS1 8760		sugar phosphate isomerase/epimerase and 4-hydroxyphenylpyruvate domain-containing protein	(C)2 -> (C)3			frame shift through insertion	959	2
**ALE 8.1**	PN96_RS0 0005 (u)			minus aa			mutation not in CDS	none	1
**ALE 8.1**	PN96_RS0 9530		regulatory protein ToxS, membrane regulatory proteins active in periplasm	G -> A	GGT -> GAT	G -> D	substitution	491	1
**ALE 8.1**	PN96_RS1 5030	*oxyR*	DNA-binding transcriptional regulator OxyR	C -> T	CTC -> TTC	L -> F	substitution	580	1
**ALE 8.1**	PN96_RS1 8700		TRAP transporter fused permease subunit	G -> C	GGC -> GCC	G -> A	substitution	1718	2

**ALE 8.1**	PN96_RS18735		LysR family transcriptional regulator	G -> C	CTT -> GTT	L -> V	substitution	997	2
**ALE 8.1**	PN96_RS18740 (u)			C -> T			mutation not in CDS	none	2
**ALE 8.2**	PN96_RS09530		regulatory protein ToxS, membrane regulatory proteins active in periplasm	G -> A	GGT -> GAT	G -> D	substitution	491	1
**ALE 8.2**	PN96_RS15030	*oxyR*	DNA-binding transcriptional regulator OxyR	C -> T	CTC -> TTC	L -> F	substitution	580	1
**ALE 8.2**	PN96_RS18700		TRAP transporter fused permease subunit	G -> C	GGC -> GCC	G -> A	substitution	1718	2
**ALE 8.2**	PN96_RS18735		LysR family transcriptional regulator	G -> C	CTT -> GTT	L -> V	substitution	997	2
**ALE 8.2**	PN96_RS18740 (u)			C -> T			mutation not in CDS	none	2
**ALE 8.2**	PN96_RS23275		D-amino-acid transaminase, amino acid metabolism and peptidoglycan biosynthesis	G -> T	CCG -> CCT		silent	597	2

Supplementary data on PHB production from PCA

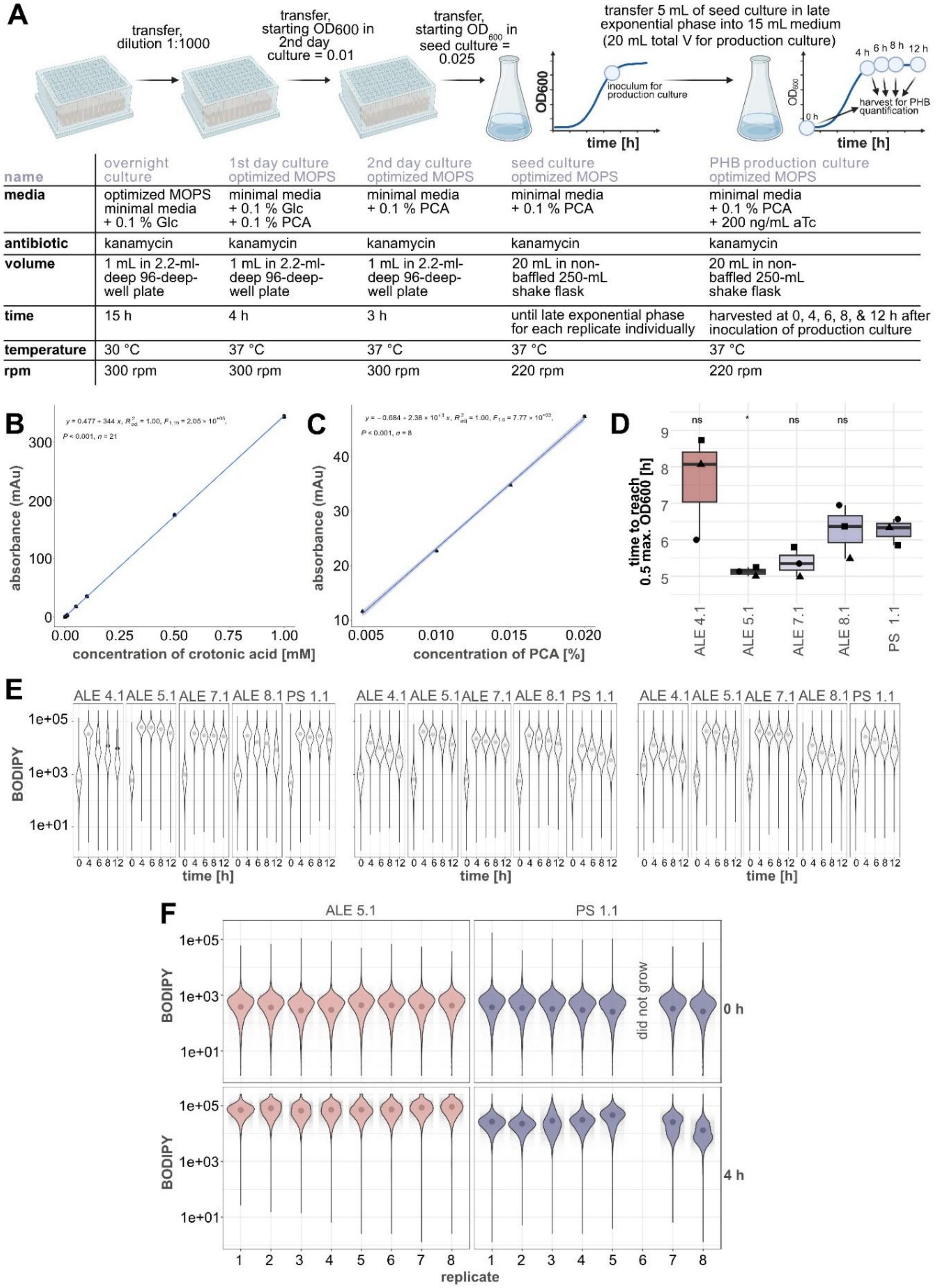

Supplementary Figure 4. Details for PHB production from PCA. A) Workflow for the production of PHB from 0.1 % PCA with *V. natriegens,* carrying the aTc-inducible *phaBAPC* plasmid. Strains were sampled for PHB quantification 4, 6, 8, and 12 hours after induction of PHB production. The 4-hour time point for PHB quantification was selected because it corresponds to approximately mid-exponential phase for the fastest-growing strain under production conditions, a period of active PHB accumulation before carbon becomes limiting. This is a standard and widely used approach in PHB production studies, when the primary aim is to compare relative production capacity across strains rather than to optimize endpoint yield. The 4-hour comparison point is therefore intentional and appropriate for the study objectives. B) Absorbance calibration curve for different concentration of crotonic acid (CA) from three technical replicates. C) Absorbance calibration curve for different concentration of PCA from two technical replicates. Different shapes show technical replicates in panel B-C. D) Time until reaching mid exponential phase (0.5 maximum OD_{600}), growth rate, and maximum OD_{600} for *V. natriegens* parental strain (PS) and four different ALE lineages in seed cultures for PHB production. Data from three technical replicates. E) BODIPY signal at different time points after induction of PHB production for *V. natriegens* parental strain (PS) and different ALE lineages. Flow cytometry data for all three technical replicates from independent days is shown in individual graphs. F) BODIPY signal at 4 h after induction of PHB production for *V. natriegens* parental strain (PS) and ALE lineage 5.1. Flow cytometry data for all eight replicates is depicted.

Supplementary information on PHA production from aromatic or lignin-derived carbon sources in representative organisms

Supplementary Table 16. Reported PCA concentrations and growth conditions for PCA catabolizing microorganisms.

Species	Description	PCA (w/v)	pH	Reference
Aspergillus nidulans	Ascomycete	0.154 %	6.5	(Kuswandi and Roberts, 1992)
Azotobacter chroococcum	Gamma proteobacterium	≤ 0.300 %	7.3	(Juarez et al., 2005)
Corynebacterium glutamicum	Actinomycete	8.27 %	7.0	(Kogure et al., 2021)
Euglena gracilis	Freshwater microalga	0.080 %	6–7	(Tan et al., 2020)
Pseudomonas putida	Gamma proteobacterium	2.17 %	7.0	(Li and Ye, 2021)
Rhodococcus jostii	Actinomycete	0.015 %	~ 7	(Spence et al., 2020)
Vibrio natriegens	Gamma proteobacterium	≤ 0.300 %	7.2	This study

Supplementary References

Ajiboye, T.O., Habibu, R.S., Saidu, K., Haliru, F.Z., Ajiboye, H.O., Aliyu, N.O., Ibitoye, O.B., Uwazie, J.N., Muritala, H.F., Bello, S.A., Yusuf, I.I., Mohammed, A.O., 2017. Involvement of oxidative stress in protocatechuic acid-mediated bacterial lethality. Microbiologyopen 6, e00472. https://doi.org/10.1002/mbo3.472

Andersson, M.M., Breccia, J.D., Hatti-Kaul, R., 2000. Stabilizing effect of chemical additives against oxidation of lactate dehydrogenase. Biotechnology and Applied Biochemistry 32, 145–153. https://doi.org/10.1042/BA20000014

Beloin, C., Valle, J., Latour-Lambert, P., Faure, P., Kzreminski, M., Balestrino, D., Haagensen, J.A.J., Molin, S., Prensier, G., Arbeille, B., Ghigo, J.-M., 2004. Global impact of mature biofilm lifestyle on *Escherichia coli* K-12 gene expression. Molecular Microbiology 51, 659–674. https://doi.org/10.1046/j.1365-2958.2003.03865.x

Brown, E.D., Vivas, E.I., Walsh, C.T., Kolter, R., 1995. MurA (MurZ), the enzyme that catalyzes the first committed step in peptidoglycan biosynthesis, is essential in *Escherichia coli*. Journal of Bacteriology 177, 4194–4197. https://doi.org/10.1128/jb.177.14.4194-4197.1995

Cui, J., Zhao, T., Zou, L., Wang, X., Zhang, Y., 2018. Molecular dynamics simulation of *S. cerevisiae* glucan destruction by plasma ROS based on ReaxFF. J. Phys. D: Appl. Phys. 51, 355401. https://doi.org/10.1088/1361-6463/aad4ec

Dik, D.A., Fisher, J.F., Mobashery, S., 2018. Cell-wall recycling of the Gram-negative bacteria and the nexus to antibiotic resistance. Chem. Rev. 118, 5952–5984. https://doi.org/10.1021/acs.chemrev.8b00277

Emami, K., Guyet, A., Kawai, Y., Devi, J., Wu, L.J., Allenby, N., Daniel, R.A., Errington, J., 2017. RodA as the missing glycosyltransferase in *Bacillus subtilis* and antibiotic discovery for the peptidoglycan polymerase pathway. Nat Microbiol 2, 16253. https://doi.org/10.1038/nmicrobiol.2016.253

Engman, J., Rogstam, A., Frees, D., Ingmer, H., von Wachenfeldt, C., 2012. The YjbH Adaptor Protein Enhances Proteolysis of the Transcriptional Regulator Spx in *Staphylococcus aureus*. Journal of Bacteriology 194, 1186–1194. https://doi.org/10.1128/jb.06414-11

Engman, J., von Wachenfeldt, C., 2015. Regulated protein aggregation: a mechanism to control the activity of the ClpXP adaptor protein YjbH. Mol Microbiol 95, 51–63. https://doi.org/10.1111/mmi.12842

Faber, A., Politan, R.J., Stukenberg, D., Morris, K.M., Kim, R., Jeon, E., Inckemann, R., Becker, A., Thuronyi, B., Fritz, G., 2025. Expanding genetic engineering capabilities in *Vibrio natriegens* with the Vnat Collection. Nucleic Acids Res 53, gkaf580. https://doi.org/10.1093/nar/gkaf580

Forsten, E., Gerdes, S., Petri, R., Büchs, J., Magnus, J., 2024. Unraveling the impact of pH, sodium concentration, and medium osmolality on *Vibrio natriegens* in batch processes. BMC Biotechnol 24. https://doi.org/10.1186/s12896-024-00897-8

Fuchs, G., Boll, M., Heider, J., 2011. Microbial degradation of aromatic compounds — from one strategy to four. Nat Rev Microbiol 9, 803–816. https://doi.org/10.1038/nrmicro2652

Fujita, M., Mori, K., Hara, H., Hishiyama, S., Kamimura, N., Masai, E., 2019. A TonB-dependent receptor constitutes the outer membrane transport system for a lignin-derived aromatic compound. Commun Biol 2. https://doi.org/10.1038/s42003-019-0676-z

Galano, A., Pérez-González, A., 2012. On the free radical scavenging mechanism of protocatechuic acid, regeneration of the catechol group in aqueous solution. Theor Chem Acc 131. https://doi.org/10.1007/s00214-012-1265-0

Gregory, G.J., Dutta, A., Parashar, V., Boyd, E.F., 2020. Investigations of dimethylglycine, glycine betaine, and ectoine uptake by a betaine-carnitine-choline transporter family transporter with diverse substrate specificity in *Vibrio* species. J Bacteriol 202, e00314-20. https://doi.org/10.1128/JB.00314-20

Hartl, F.U., Hayer-Hartl, M., 2002. Molecular chaperones in the cytosol: from nascent chain to folded protein. Science 295, 1852–1858. https://doi.org/10.1126/science.1068408

Hosaka, M., Kamimura, N., Toribami, S., Mori, K., Kasai, D., Fukuda, M., Masai, E., 2013. Novel tripartite aromatic acid transporter essential for terephthalate uptake in *Comamonas* sp. strain E6. Applied and Environmental Microbiology 79, 6148–6155. https://doi.org/10.1128/AEM.01600-13

Hu, X., Chen, Z., Xiong, K., Wang, J., Rao, X., Cong, Y., 2016. Vi capsular polysaccharide: Synthesis, virulence, and application. Critical Reviews in Microbiology 43, 440–452. https://doi.org/10.1080/1040841X.2016.1249335

Imlay, J.A., Chin, S.M., Linn, S., 1988. Toxic DNA damage by hydrogen peroxide through the Fenton Reaction in vivo and in vitro. Science 240, 640–642. https://doi.org/10.1126/science.2834821

Jo, G.-A., Lee, J.M., No, G., Kang, D.S., Kim, S.-H., Ahn, S.-H., Kong, I.-S., 2015. Isolation and characterization of a 17-kDa FKBP-type peptidyl-prolyl cis/trans isomerase from *Vibrio anguillarum*. Protein Expression and Purification 110, 130–137. https://doi.org/10.1016/j.pep.2015.02.019

Juarez, B., Martinez-Toledo, M.V., Gonzalez-Lopez, J., 2005. Growth of *Azotobacter chroococcum* in chemically defined media containing p-hydroxybenzoic acid and protocatechuic acid. Chemosphere 59, 1361–1365. https://doi.org/10.1016/j.chemosphere.2004.11.037

Kamimura, N., Aoyama, T., Yoshida, R., Takahashi, K., Kasai, D., Abe, T., Mase, K., Katayama, Y., Fukuda, M., Masai, E., 2010. Characterization of the protocatechuate 4,5-cleavage pathway operon in *Comamonas* sp. Strain E6 and discovery of a novel pathway gene. Appl Environ Microbiol 76, 8093–8101. https://doi.org/10.1128/AEM.01863-10

Kogure, T., Suda, M., Hiraga, K., Inui, M., 2021. Protocatechuate overproduction by *Corynebacterium glutamicum* via simultaneous engineering of native and heterologous biosynthetic pathways. Metabolic Engineering 65, 232–242. https://doi.org/10.1016/j.ymben.2020.11.007

Kumar, M., Singhal, A., Verma, P.K., Thakur, I.S., 2017. Production and characterization of polyhydroxyalkanoate from lignin derivatives by *Pandoraea* sp. ISTKB. ACS Omega 2, 9156–9163. https://doi.org/10.1021/acsomega.7b01615

Kuswandi, K., Roberts, C.F., 1992. Genetic control of the protocatechuic acid pathway in *Aspergillus nidulans*. Microbiology 138, 817–823. https://doi.org/10.1099/00221287-138-4-817

Lee, H.H., Ostrov, N., Wong, B.G., Gold, M.A., Khalil, A.S., Church, G.M., 2016. *Vibrio natriegens*, a new genomic powerhouse. bioRxiv. https://doi.org/10.1101/058487

Li, J., Ye, B.-C., 2021. Metabolic engineering of Pseudomonas putida KT2440 for high-yield production of protocatechuic acid. Bioresource Technology 319, 124239. https://doi.org/10.1016/j.biortech.2020.124239

Lushchak, V.I., 2001. Oxidative stress and mechanisms of protection against it in bacteria. Biochemistry (Moscow) 66, 476–489. https://doi.org/10.1023/A:1010294415625

Morya, R., Kumar, M., Kumar, V., Thakur, I.S., 2021a. Biovalorization of lignin derived compounds with molasses as co-substrate for polyhydroxyalkanoate production. Environmental Technology & Innovation 23, 101695. https://doi.org/10.1016/j.eti.2021.101695

Martinez, J.M., 2023. Proteostasis in *Bacillus subtilis* (Doctorial Dissertation). Lund University, Lund, Sweden.

Nakano, S., Küster-Schöck, E., Grossman, A.D., Zuber, P., 2003. Spx-dependent global transcriptional control is induced by thiol-specific oxidative stress in *Bacillus subtilis*. Proc Natl Acad Sci U S A 100, 13603–13608. https://doi.org/10.1073/pnas.2235180100

Nichols, N.N., Harwood, C.S., 1997. PcaK, a high-affinity permease for the aromatic compounds 4-hydroxybenzoate and protocatechuate from *Pseudomonas putida*. Journal of Bacteriology 179, 5056–5061. https://doi.org/10.1128/jb.179.16.5056-5061.1997

Ongagna-Yhombi, S.Y., McDonald, N.D., Boyd, E.F., 2014. Deciphering the role of multiple betaine-carnitine-choline transporters in the halophile *Vibrio parahaemolyticus*. Appl Environ Microbiol 81, 351–363. https://doi.org/10.1128/AEM.02402-14

Pakotiprapha, D., Samuels, M., Shen, K., Hu, J.H., Jeruzalmi, D., 2012. Structure and mechanism of the UvrA–UvrB DNA damage sensor. Nat Struct Mol Biol 19, 291–298. https://doi.org/10.1038/nsmb.2240

Patrauchan, M.A., Florizone, C., Dosanjh, M., Mohn, W.W., Davies, J., Eltis, L.D., 2005. Catabolism of benzoate and phthalate in *Rhodococcus* sp. strain RHA1: Redundancies and Convergence. Journal of Bacteriology 187, 4050–4063. https://doi.org/10.1128/jb.187.12.4050-4063.2005

Paudel, A., Panthee, S., Hamamoto, H., Grunert, T., Sekimizu, K., 2021. YjbH regulates virulence genes expression and oxidative stress resistance in *Staphylococcus aureus*. Virulence 12, 470–480. https://doi.org/10.1080/21505594.2021.1875683

Pernstich, C., Senior, L., MacInnes, K.A., Forsaith, M., Curnow, P., 2014. Expression, purification and reconstitution of the 4-hydroxybenzoate transporter PcaK from *Acinetobacter* sp. ADP1. Protein Expression and Purification 101, 68–75. https://doi.org/10.1016/j.pep.2014.05.011

Pinho, M.G., Filipe, S.R., de Lencastre, H., Tomasz, A., 2001. Complementation of the essential peptidoglycan transpeptidase function of penicillin-binding protein 2 (PBP2) by the drug resistance protein PBP2A in *Staphylococcus aureus*. Journal of Bacteriology 183, 6525–6531. https://doi.org/10.1128/jb.183.22.6525-6531.2001

Politan, R.J., Della Valle, S., Pineda, L., Joshi, J., Euler, C., Flematti, G., Fritz, G., 2025. Establishing *Vibrio natriegens* as a high-performance host for acetate-based poly-3-hydroxybutyrate production. Metabolic Engineering 92, 22–38. https://doi.org/10.1016/j.ymben.2025.07.003

Pucci, M.J., Thanassi, J.A., Ho, H.T., Falk, P.J., Dougherty, T.J., 1995. *Staphylococcus haemolyticus* contains two *D*-glutamic acid biosynthetic activities, a glutamate racemase and a D-amino acid transaminase. Journal of Bacteriology 177, 336–342. https://doi.org/10.1128/jb.177.2.336-342.1995

Ramírez-Morales, J.E., Czichowski, P., Besirlioglu, V., Regestein, L., Rabaey, K., Blank, L.M., Rosenbaum, M.A., 2021. Lignin aromatics to PHA polymers: Nitrogen and oxygen are the key factors for *Pseudomonas*. ACS Sustainable Chem. Eng. 9, 10579–10590. https://doi.org/10.1021/acssuschemeng.1c02682

Rosa, L.T., Bianconi, M.E., Thomas, G.H., Kelly, D.J., 2018. Tripartite ATP-independent periplasmic (TRAP) transporters and Tripartite Tricarboxylate Transporters (TTT): From uptake to pathogenicity. Frontiers in Cellular and Infection Microbiology 8. https://doi.org/10.3389/fcimb.2018.00033

Salmon, R.C., Cliff, M.J., Rafferty, J.B., Kelly, D.J., 2013. The CouPSTU and TarPQM transporters in *Rhodopseudomonas palustris*: redundant, promiscuous uptake systems for lignin-derived aromatic substrates. PLoS One 8, e59844. https://doi.org/10.1371/journal.pone.0059844

Salvachúa, D., Rydzak, T., Auwae, R., De Capite, A., Black, B.A., Bouvier, J.T., Cleveland, N.S., Elmore, J.R., Furches, A., Huenemann, J.D., Katahira, R., Michener, W.E., Peterson, D.J., Rohrer, H., Vardon, D.R., Beckham, G.T., Guss, A.M., 2020. Metabolic engineering of *Pseudomonas putida* for increased polyhydroxyalkanoate production from lignin. Microbial Biotechnology 13, 290–298. https://doi.org/10.1111/1751-7915.13481

Schramm, F.D., Schroeder, K., Jonas, K., 2020. Protein aggregation in bacteria. FEMS Microbiol Rev 44, 54–72. https://doi.org/10.1093/femsre/fuz026

Seixas, A.F., Quendera, A.P., Sousa, J.P., Silva, A.F.Q., Arraiano, C.M., Andrade, J.M., 2022. Bacterial Response to oxidative stress and RNA oxidation. Front. Genet. 12. https://doi.org/10.3389/fgene.2021.821535

Shi, Y., Yan, X., Li, Q., Wang, X., Liu, M., Xie, S., Chai, L., Yuan, J., 2017. Directed bioconversion of Kraft lignin to polyhydroxyalkanoate by *Cupriavidus basilensis* B-8 without any pretreatment. Process Biochemistry 52, 238–242. https://doi.org/10.1016/j.procbio.2016.10.004

Shilova, S.A., Khrenova, M.G., Matyuta, I.O., Nikolaeva, A.Y., Rakitina, T.V., Klyachko, N.L., Minyaev, M.E., Boyko, K.M., Popov, V.O., Bezsudnova, E.Y., 2023. To the understanding of catalysis by *D*-amino acid transaminases: A case study of the enzyme from *Aminobacterium colombiense*. Molecules 28, 2109. https://doi.org/10.3390/molecules28052109

Spence, E.M., Scott, H.T., Dumond, L., Calvo-Bado, L., di Monaco, S., Williamson, J.J., Persinoti, G.F., Squina, F.M., Bugg, T.D.H., 2020. The hydroxyquinol degradation pathway in *Rhodococcus jostii* RHA1 and *Agrobacterium* species is an alternative pathway for degradation of protocatechuic acid and lignin fragments. Applied and Environmental Microbiology 86, e01561-20. https://doi.org/10.1128/AEM.01561-20

Stukenberg, D., Hensel, T., Hoff, J., Daniel, B., Inckemann, R., Tedeschi, J.N., Nousch, F., Fritz, G., 2021. The Marburg Collection: A Golden Gate DNA Assembly framework for synthetic biology applications in *Vibrio natriegens*. ACS Synthetic Biology 10, 1904–1919. https://doi.org/10.1021/ACSSYNBIO.1C00126/SUPPL_FILE/SB1C00126_SI_001.PDF

Stukenberg, D., Hoff, J., Faber, A., Becker, A., 2022. NT-CRISPR, combining natural transformation and CRISPR-Cas9 counterselection for markerless and scarless genome editing in *Vibrio natriegens*. Communications Biology 5, 1–13. https://doi.org/10.1038/s42003-022-03150-0

Tan, J., Li, Y., Hou, D.-X., Wu, S., 2019. The effects and mechanisms of cyanidin-3-glucoside and its phenolic metabolites in maintaining intestinal integrity. Antioxidants (Basel) 8, 479. https://doi.org/10.3390/antiox8100479

Tan, X., Zhu, J., Wakisaka, M., 2020. Effect of protocatechuic acid on *Euglena gracilis* growth and accumulation of metabolites. Sustainability 12, 9158. https://doi.org/10.3390/su12219158

Tang, H., Wang, M.-J., Gan, X.-F., Li, Y.-Q., 2022. Funneling lignin-derived compounds into polyhydroxyalkanoate by *Halomonas* sp. Y3. Bioresource Technology 362, 127837. https://doi.org/10.1016/j.biortech.2022.127837

Tang, W., Chen, Q., Liu, D., Zhang, L., Ding, M., 2025. Damage to the peptidoglycan in the cell wall caused by reactive oxygen species: A molecular dynamics simulation. J. Phys. Chem. B 129, 6363–6371. https://doi.org/10.1021/acs.jpcb.5c01612

Thomas, H.E., Boas Lichty, K.E., Richards, G.P., Boyd, E.F., 2025. Dual roles of glycine betaine, dimethylglycine, and sarcosine as osmoprotectants and nutrient sources for *Vibrio natriegens*. Applied and Environmental Microbiology 91, e00619-25. https://doi.org/10.1128/aem.00619-25

Tomizawa, S., Chuah, J.-A., Matsumoto, K., Doi, Y., Numata, K., 2014. Understanding the limitations in the biosynthesis of polyhydroxyalkanoate (PHA) from lignin derivatives. ACS Sustainable Chem. Eng. 2, 1106–1113. https://doi.org/10.1021/sc500066f

Wachi, M., Doi, M., Okada, Y., Matsuhashi, M., 1989. New *mre* genes *mreC* and *mreD*, responsible for formation of the rod shape of *Escherichia coli* cells. Journal of Bacteriology 171, 6511–6516. https://doi.org/10.1128/jb.171.12.6511-6516.1989

Wang, X., Lin, L., Dong, J., Ling, J., Wang, W., Wang, H., Zhang, Z., Yu, X., 2018. Simultaneous improvements of *Pseudomonas* cell growth and polyhydroxyalkanoate production from a lignin derivative for lignin-consolidated bioprocessing. Applied and Environmental Microbiology 84, e01469-18. https://doi.org/10.1128/AEM.01469-18

Wang, Y., Hu, Y.-S., Tang, H., Li, Y.-Q., Luo, C.-B., 2025. Highly efficient conversion of lignin-derived aromatic compounds into polyhydroxyalkanoate using *Halomonas hydrothermalis* LL1. Industrial Crops and Products 236, 122122. https://doi.org/10.1016/j.indcrop.2025.122122

Yusupov, M., Bogaerts, A., Huygh, S., Snoeckx, R., van Duin, A.C.T., Neyts, E.C., 2013. Plasma-induced destruction of bacterial cell wall components: A reactive molecular dynamics simulation. J. Phys. Chem. C 117, 5993–5998. https://doi.org/10.1021/jp3128516

Yusupov, M., Neyts, E.C., Khalilov, U., Snoeckx, R., van Duin, A.C.T., Bogaerts, A., 2012. Atomic-scale simulations of reactive oxygen plasma species interacting with bacterial cell walls. New J. Phys. 14, 093043. https://doi.org/10.1088/1367-2630/14/9/093043

Zakrzewska-Czerwińska, J., Jakimowicz, D., Zawilak-Pawlik, A., Messer, W., 2007. Regulation of the initiation of chromosomal replication in bacteria. FEMS Microbiol Rev 31, 378–387. https://doi.org/10.1111/j.1574-6976.2007.00070.x

Ziegler, C., Bremer, E., Krämer, R., 2010. The BCCT family of carriers: from physiology to crystal structure. Mol Microbiol 78, 13–34. https://doi.org/10.1111/j.1365-2958.2010.07332.x
